# Effectiveness of elastic therapeutic taping in pain management after median sternotomy: A randomized controlled trial

**DOI:** 10.1371/journal.pone.0359282

**Published:** 2026-09-28

**Authors:** Alp Özel, Umut Ata Uğraş, Muhammet Fatih Uysal, Erhan Renan Uçaroğlu

**Affiliations:** 1 Department of Physiotherapy and Rehabilitation, Faculty of Health Sciences, Bolu Abant Izzet Baysal University, Bolu, Turkey; 2 Department of Cardiovascular Surgery, Faculty of Medicine, Bolu Abant Izzet Baysal University, Bolu, Turkey; Ufuk University Faculty of Medicine: Ufuk Universitesi Tip Fakultesi, TÜRKIYE

## Abstract

To evaluate the effectiveness of elastic therapeutic taping on postoperative pain, functional mobility, sleep quality, analgesic consumption, and length of hospital stay in patients undergoing coronary artery bypass grafting (CABG) via median sternotomy. In this prospective, randomized, double-blind, placebo- and sham-controlled trial, 195 patients undergoing elective CABG were randomly assigned to elastic therapeutic taping (n = 65), placebo taping without tension (n = 65), or sham taping to a non-related region (n = 65). Elastic therapeutic taping was applied after patients were transferred from the intensive care unit to the cardiovascular surgery ward. Pain at rest and during coughing was assessed using the Visual Analog Scale (VAS) at baseline and 12, 24, and 48 hours. Secondary outcomes were Timed Up and Go (TUG), sleep quality, total analgesic use, and length of hospital stay. Data were analyzed using a prespecified two-way mixed ANOVA with Bonferroni-adjusted post hoc comparisons for the primary pain outcome. Nonparametric secondary outcomes were analyzed using Kruskal–Wallis or Friedman tests, as appropriate. At 48 hours, resting pain was lower in the elastic therapeutic taping group (19.0 ± 10.2) than placebo taping without tension group (37.5 ± 13.7) and sham taping group (41.2 ± 16.2; p = 0.001). The observed between-group reduction in pain exceeded the reported minimal clinically important difference (MCID) of 9.9 mm for acute postoperative pain, indicating a clinically meaningful treatment effect. Coughing pain was also reduced (39.2 ± 17.0 vs. 56.3 ± 17.2 and 59.4 ± 16.8; p = 0.001). Tramadol use was lower with elastic therapeutic taping (50 ± 50 mg vs. 100 ± 50 mg; p = 0.038). Elastic therapeutic taping was associated with a shorter length of hospital stay (4 [3–5] vs. 5 [4–7] days; p = 0.042). Functional mobility (TUG, p = 0.002) and sleep quality (p = 0.001) were also significantly improved in the elastic therapeutic taping group compared with the placebo and sham groups. Elastic therapeutic taping was associated with reduced pain, lower opioid use, improved early recovery, and a shorter length of hospital stay after median sternotomy.

**Trial registration:**

ClinicalTrials.gov NCT06910215

## Introduction

Despite advances in surgical and anesthetic techniques, acute postoperative pain remains a common and clinically relevant problem following coronary artery bypass grafting (CABG) via median sternotomy, affecting approximately 77–85% of patients [1]. Sternotomy-related pain delays early mobilization, impairs respiratory function, increases pulmonary complication risk, and contributes to prolonged hospitalization [2]. In addition, increased analgesic use, including opioids, can further complicate recovery by causing adverse effects such as respiratory depression, nausea, and constipation [3].

For this reason, there is increasing interest in multimodal, opioid-sparing pain management strategies after sternotomy. Elastic therapeutic taping (ETT) is a non-pharmacological intervention involving the application of elastic adhesive tape to the skin with controlled tension. It is thought to modulate pain through continuous cutaneous and proprioceptive stimulation while providing gentle biomechanical support that may reduce tissue stress during movement. Although the precise mechanisms remain uncertain, these neurophysiological and biomechanical effects have been proposed to contribute to pain reduction and improved functional recovery [4]. ETT has been shown to have beneficial effects on musculoskeletal conditions, shoulder dysfunction, and management of lymphedema [5].

Although recent studies suggest that ETT may reduce post-sternotomy pain, most have been limited by small sample sizes and single-center designs [4,6]. Moreover, in physical interventions such as taping, inadequate blinding may allow placebo and sham procedures to be distinguishable from the active treatment, increasing the risk of bias [7]. This challenge has been highlighted as a major methodological concern in non-pharmacological trials by the CONSORT statement and related guidelines [8,9].

To the best of the investigators’ knowledge, no adequately powered, double-blind randomized trial has comprehensively evaluated the opioid-sparing and functional recovery effects of sternum-targeted ETT following CABG. The aim of this randomized, double-blind, placebo- and sham-controlled trial was to evaluate the effects of ETT on pain, analgesic use, and functional recovery after CABG via median sternotomy. We hypothesized that participants receiving ETT would report lower pain scores and require less pharmacological support than those in the placebo and sham groups.

## Materials and methods

### Study design

This single-center, randomized, double-blind, placebo- and sham-controlled, parallel-group clinical trial evaluated the effects of ETT on postoperative pain and functional recovery after elective CABG via median sternotomy. The study was conducted at the Department of Cardiovascular Surgery, Izzet Baysal Training and Research Hospital, Bolu, Turkey. Participants were recruited between January 22 and December 24, 2025. No changes were made to the trial design, eligibility criteria, interventions, or outcome measures after trial commencement.

### Randomization and blinding

Participants were randomized (1:1:1) to the ETT, placebo, or sham groups using a computer-generated block randomization sequence generated with the Blockrand package in R (v4.5.2) before participant recruitment, using fixed blocks of six participants. The randomization sequence was generated and retained by the corresponding author before participant recruitment and was not accessible to the cardiovascular surgery team responsible for participant enrollment. Eligible participants were enrolled after screening and informed consent procedures. Following enrollment, the corresponding author identified the next assignment in the pre-generated randomization sequence and communicated it to the physiotherapist responsible for tape application immediately before the intervention. Because of the nature of the intervention, the physiotherapist responsible for tape application could not be blinded, but had no role in participant enrollment, outcome assessment, or statistical analysis.

### Ethical approval and informed consent

The study was approved by the Bolu Abant Izzet Baysal University Non-Interventional Clinical Research Ethics Committee (Approval No: 2025/07; Date: January 21, 2025) and conducted in accordance with the principles of the Declaration of Helsinki. Written informed consent was obtained from all participants prior to enrollment and before any study-related procedures were performed.

The trial was registered retrospectively at ClinicalTrials.gov (NCT06910215; https://clinicaltrials.gov/study/NCT06910215). Participant recruitment began on January 22, 2025, whereas the trial record was first released on March 25, 2025, and publicly posted on April 2, 2025. The delayed registration resulted from administrative procedures during trial setup. Importantly, no changes were made to the study design, eligibility criteria, interventions, outcomes, sample size calculation, or statistical analysis plan after participant enrollment began or after trial registration. The registry record reflects the original study protocol approved by the institutional ethics committee prior to recruitment. The authors confirm that all ongoing and related trials for this intervention are registered. The elastic therapeutic tapes used during the intervention were CE-certified for medical use.

### Participants

A total of 238 individuals scheduled for elective CABG via median sternotomy were assessed for eligibility. Of these, 203 were randomized, and 195 were included in the final analysis. Baseline assessments were performed 6–8 hours after transfer from the intensive care unit (ICU) to the ward following clinical stabilization. Before tape application, pain at rest, pain during coughing, oxygen saturation, sleep quality, anxiety, and fatigue were assessed.

### Inclusion and exclusion criteria

Male and female patients aged 18–75 years who were scheduled for elective CABG via median sternotomy, were able to understand and communicate effectively, and provided written informed consent were eligible for inclusion. Exclusion criteria were active skin disease or open wounds at the sternotomy site, known tape hypersensitivity, neurological or psychiatric disorders affecting cognition, and use of medications influencing pain perception. Additional exclusions included acute renal or hepatic failure, preoperative mechanical ventilation, planned reoperation, participation in another trial within 30 days, or revision surgery. Participant flow is presented in Fig 1.

**Fig 1:**
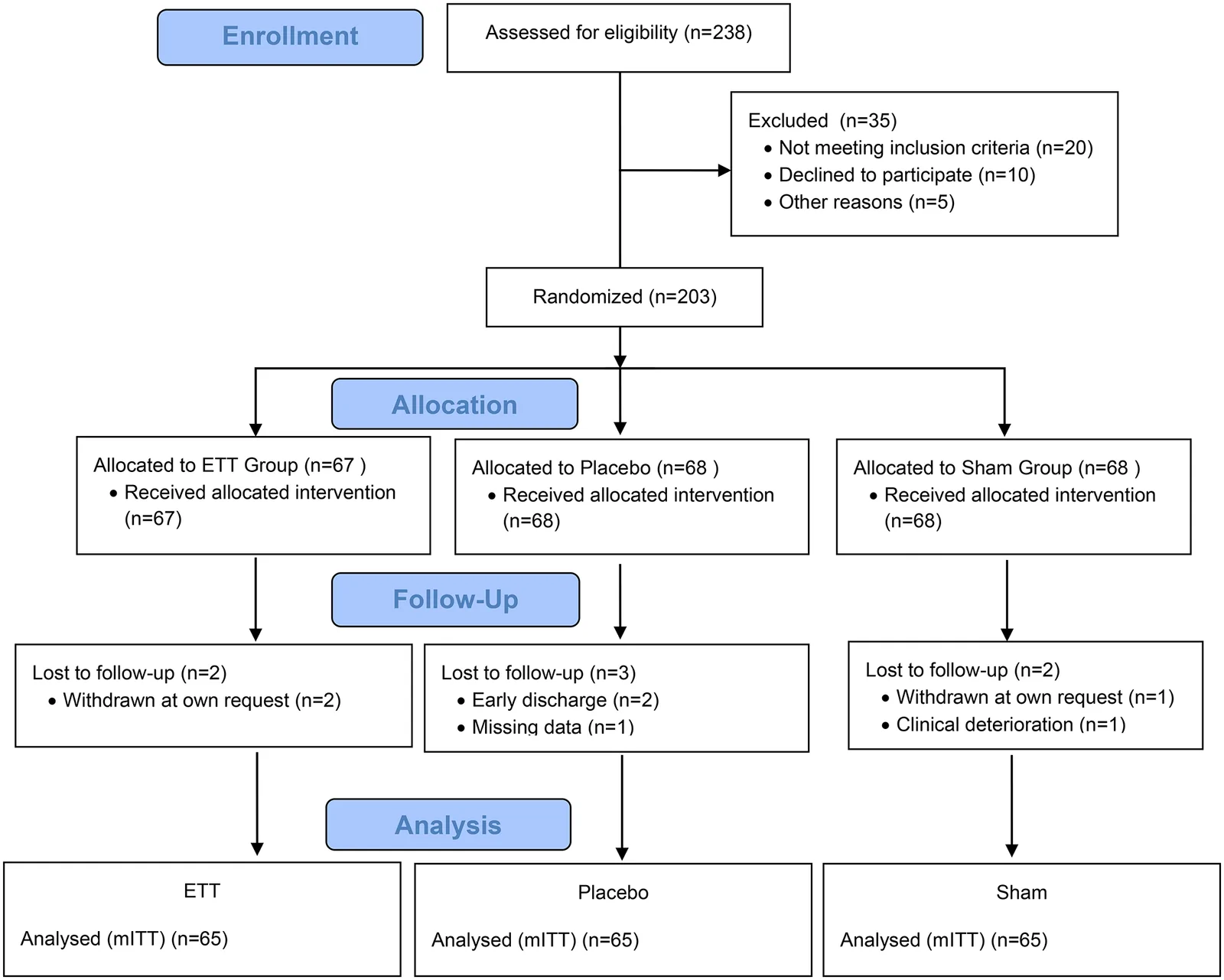
Flow diagram of participant screening, enrollment, randomization, and follow-up.

#### Interventions.

To ensure standardization, all taping procedures were performed by a certified physiotherapist with more than 10 years of experience, within 6–8 hours after the participant’s transfer to the cardiovascular surgery ward. In addition to the allocated taping intervention, all participants received the institution’s standard postoperative care following CABG. This included routine medical management, respiratory physiotherapy (diaphragmatic breathing exercises, coughing exercises, incentive spirometry, and chest percussion), early mobilization, nursing care, and standard postoperative analgesia. Postoperative care was continued until hospital discharge according to routine institutional practice. To ensure intervention fidelity, all taping procedures were performed by the same certified physiotherapist using predefined anatomical landmarks and a standardized application protocol.

#### Elastic therapeutic taping group.

Elastic therapeutic tape (Kinesio® Tex Gold, Kinesio Holding Corporation, USA) was applied bilaterally approximately 3 cm lateral to the sternotomy incision in a craniocaudal direction along the anterior thoracic wall. Five-centimeter tapes were applied with 25–35% tension, with the ends placed without tension and the incision avoided. All applications followed a standardized anatomical template to enhance mechanoreceptive and proprioceptive input.

#### Placebo taping group.

The same tape was applied to the same anatomical regions without tension and without therapeutic principles.

#### Sham taping group.

Tape was applied without tension to anatomically unrelated regions (scapular or paraspinal areas) to control therapist contact. All sham applications were performed by the same practitioner, using a standardized contact duration and a predefined template. All groups received the same standard postoperative analgesia protocol.

In all three groups, the tape was applied once and remained in place until completion of the 48-hour follow-up period unless early removal was required because of skin irritation or tape detachment. Representative photographs illustrating the ETT, placebo, and sham taping procedures are presented in Fig 2.

**Fig 2:**
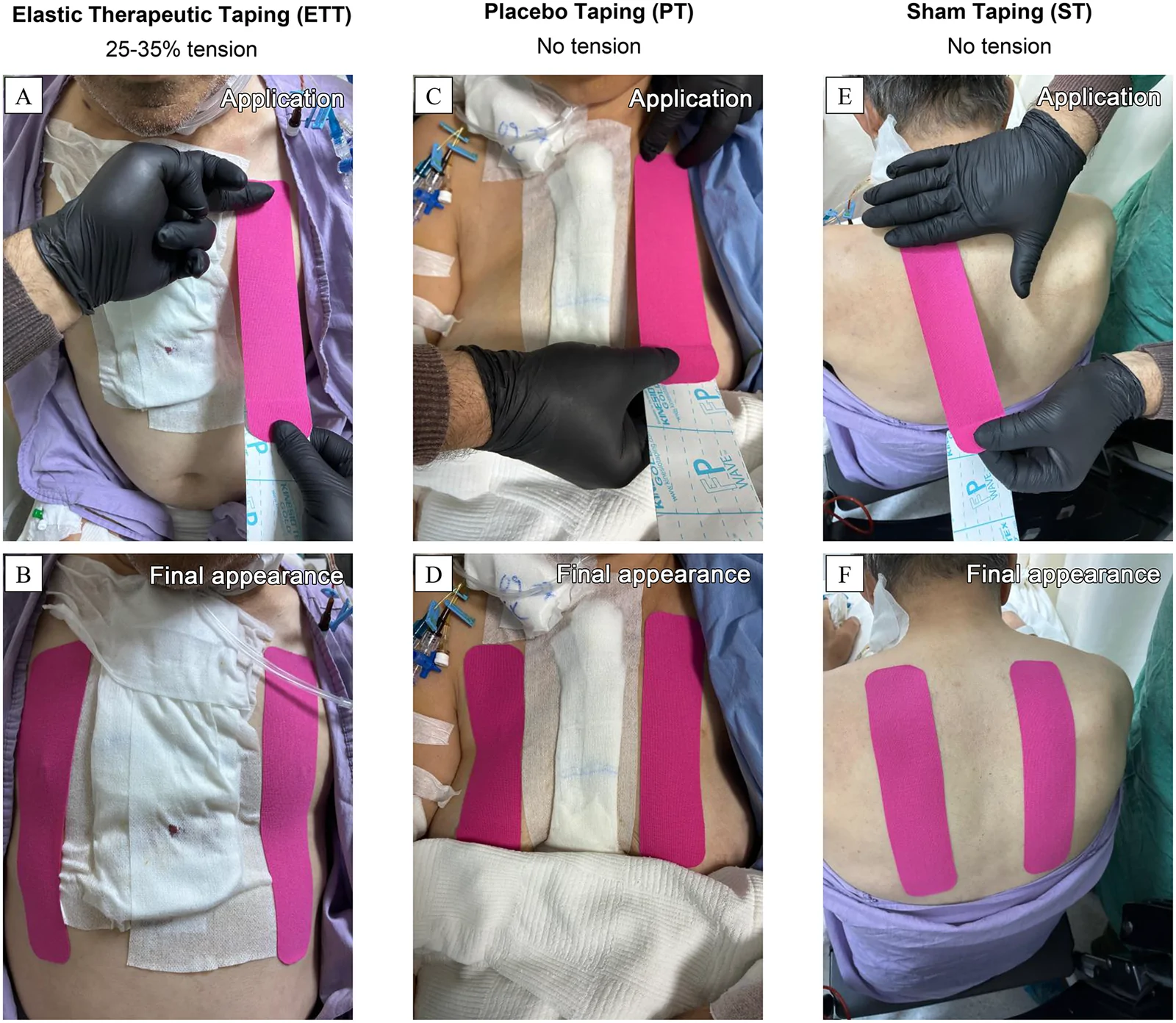
Representative photographs of the taping procedures. (A–B) Elastic therapeutic taping (ETT). (C–D) Placebo taping (PT). (E–F) Sham taping (ST). Clinical photographs are published with written informed consent from the participants.

### Data collection and measurements

Data collection began 6–8 hours after transfer from the ICU to the ward. Pain at rest, pain during coughing, and oxygen saturation were assessed at baseline and at 12, 24, and 48 hours post-intervention. Sleep quality was assessed at baseline and again at 48 hours, whereas functional mobility (Timed Up and Go test) was evaluated only at 48 hours. Anxiety and fatigue were assessed only at baseline. The intervention was administered by one physiotherapist who was not involved in outcome assessment. All outcome assessments were performed by a second physiotherapist throughout the study. Because all measurements were obtained by the same assessor, inter-rater reliability was not applicable. Only the outcome measures and assessment time points relevant to the predefined objectives of the present report are presented in this manuscript.

### Primary outcome

#### Pain intensity.

Pain intensity was assessed using a 100-mm Visual Analog Scale (VAS), a valid and reliable instrument for measuring acute postoperative pain [10,11]. Scores were recorded at rest and during coughing at each time point. The sensitivity of this scale for dynamically monitoring postoperative pain has been highlighted in recent studies [4].

### Secondary outcomes

#### Oxygen saturation (SpO_2_).

Peripheral SpO_2_ was measured at each time point using a portable pulse oximeter (H100B, Edan Instruments, Shenzhen, China), with values averaged from three consecutive readings [12]. Pulse oximetry is a valid and reliable method for non-invasive assessment of peripheral oxygen saturation [13]

#### Sleep quality, fatigue, and anxiety.

VAS-based assessments have demonstrated acceptable validity and reliability for evaluating acute postoperative symptoms, including anxiety and fatigue, and have been used to monitor changes in perceived sleep quality [14–16].

#### Functional mobility.

Functional mobility was assessed using standardized single-task Timed Up and Go (TUG) test [17]. Participants stood up from a chair, walked 3 meters, turned around, and returned to a seated position, with completion time recorded in seconds. A research assistant accompanied participants throughout the test to ensure safety. TUG test is a valid and reliable measure of functional mobility in cardiovascular populations [17].

**Analgesic use and length of hospital stay (LOS):** Analgesic use during the first 48 postoperative hours was categorized as intravenous paracetamol, non-steroidal anti-inflammatory drugs (NSAIDs), and weak opioids (tramadol). Intravenous paracetamol (1000 mg) was administered as part of the standard postoperative analgesic protocol; however, total paracetamol consumption could vary according to individual pain levels and clinical requirements. Rescue tramadol (50 mg) was administered when VAS ≥ 40 mm and repeated as needed. Total paracetamol and tramadol doses were calculated cumulatively (mg), and NSAID use was recorded as yes/no. NSAID administration was not protocolized and was left to the discretion of the treating physician according to routine clinical practice. Analgesic data were verified using nursing records and the hospital information system. LOS was defined as the number of days from surgery to hospital discharge, including both ICU and ward stays. Hospital discharge was determined by the cardiovascular surgery team according to routine institutional clinical criteria and was independent of the study investigators.

#### Safety monitoring.

Skin reactions (redness, irritation, itching) and other adverse events were monitored and documented. No serious adverse events occurred during the study. Two participants in the ETT group experienced mild skin redness, and one participant in the placebo group reported mild itching. No adverse events were observed in the sham group. None of these events required medical treatment, resulted in premature tape removal, or led to study withdrawal. No patients required ICU readmission or reoperation during the study period, and no major postoperative complications were observed.

### Sample size calculation

A priori sample size calculation was performed using G*Power software (v3.1.9.7). Based on the pooled standardized mean difference for postoperative pain after median sternotomy reported by King et al., an effect size of 0.49 was selected as a conservative estimate of the expected between-group difference in the primary outcome for the a priori sample size calculation [18]. With a two-sided α = 0.05 and 80% power (1 − β = 0.80), the required sample size was calculated as 65 participants per group (195 in total). Recruitment continued until the target number of 195 evaluable participants was achieved. Consequently, 203 participants were randomized, and 195 completed the study and were included in the final analysis.

### Statistics

Statistical analyses were performed using SPSS (v20.0) and R Studio (v4.5.2). Normality and variance homogeneity were assessed with the Shapiro–Wilk and Levene’s tests. Continuous data are presented as mean ± standard deviation (SD) or median (interquartile range-IQR), and categorical data as frequencies and percentages.

Baseline characteristics were compared using one-way ANOVA, Kruskal–Wallis, χ², or Fisher’s exact tests as appropriate. The primary outcome (VAS) was analyzed using a two-way mixed ANOVA (group × time), which was selected a priori to evaluate between-group, within-group, and group-by-time interaction effects across repeated measurements. Given the balanced study design and equal group sizes, mixed ANOVA was considered appropriate because of its robustness to moderate departures from normality. Bonferroni-adjusted post-hoc comparisons were performed when appropriate. Effect sizes were reported as partial η² and Cohen’s d. SpO_2_, was analyzed using Friedman test. Because sleep quality, fatigue, TUG, and length of hospital stay data did not satisfy the assumption of normality according to the Shapiro-Wilk test, these variables were summarized as median (interquartile range) and analyzed using non-parametric methods. Sleep quality, fatigue, and TUG were compared using Kruskal–Wallis tests. All analyses were two-tailed (α = 0.05). Analyses were conducted using a modified intention-to-treat (mITT) approach, including all randomized participants who received the allocated intervention and had at least one evaluable post-intervention outcome assessment. Participants with no post-intervention outcome data were excluded because no post-intervention data were available for analysis. No imputation of missing outcome data was performed because only seven participants (3.4%) were lost to follow-up. Therefore, the primary analysis represents a complete-case mITT approach. The final sample (n = 195) met the a priori power requirement.

## Results and discussion

A total of 238 patients were assessed for eligibility. Thirty-five patients were excluded (20 did not meet the inclusion criteria, 10 declined to participate, and 5 were excluded for other reasons). Consequently, 203 participants were randomized to the ETT group (n = 67), placebo group (n = 68), or sham group (n = 68).

All randomized participants received their allocated intervention. During follow-up, 2 participants in the ETT group (withdrawal at own request), 3 in the placebo group (2 early discharge, 1 missing data), and 2 in the sham group (1 withdrawal at own request, 1 clinical deterioration) were lost to follow-up. A total of 195 participants (65 per group) were included in the final analysis.

### Demographic and clinical characteristics

Baseline demographic and clinical characteristics of the 195 participants are shown in Table 1. Groups were similar in baseline demographics and clinical characteristics. Likewise, baseline resting and coughing VAS scores were comparable.

**Table 1:** Baseline demographic, surgical, and clinical characteristics of the participants.

Variable	ETT Group (n = 65)	PT Group (n = 65)	ST Group (n = 65)	p
**Demographic Data**				
**Age (years) Mean ± SD [Min–Max])**	61.2 ± 8.4 [45–74]	60.8 ± 7.9 [48–75]	61.5 ± 8.1 [44–75]	0.878^a^
**BMI (kg/m2) Mean ± SD [Min-Max])**	27.8 ± 4.2 [20.5–35.1]	28.1 ± 3.8 [21.0–34.5]	27.6 ± 4.1 [19.8–36.0]	0.781^a^
**Male sex, n (%)**	48 (73.8%)	50 (76.9%)	47 (72.3%)	0.828^c^
**Smoking status (yes), n (%)**	22 (33.8%)	20 (30.8%)	24 (36.9%)	0.760^c^
**Surgical Data**				
**Operation time (min) Mean ± SD [Min–Max])**	215 ± 42 [160–310]	210 ± 38 [155–300]	218 ± 45 [165–320]	0.574^a^
**Number of grafts Median, (IQR) [Min–Max])**	3 (2–4) [1–5]	3 (2–4) [1–5]	3 (2–3) [1–4]	0.147^b^
**Extubation time (hours) Median, (IQR) [Min–Max])**	7.4 (6.2–8.8) [5–14]	7.2 (6.0–8.5) [5–12]	7.5 (6.3–9.1) [5–15]	0.497^b^
**Baseline Clinical Scores**				
**VAS Pain – Resting Median, (IQR) [Min–Max])**	65 (55–78) [30–95]	62 (52–75) [25–90]	64 (54–80) [35–95]	0.454^b^
**VAS Pain – Coughing Median, (IQR) [Min–Max])**	80 (70–90) [60–100]	78 (68–88) [55–98]	81 (72–92) [65–100]	0.158^b^
**Sleep Quality – Baseline Median, (IQR) [Min–Max])**	45 (35–58) [10–80]	48 (38–60) [15–85]	44 (32–55) [10–75]	0.260^b^
**Anxiety – Baseline Median, (IQR) [Min–Max])**	52 (40–65) [15–90]	50 (38–62) [10–85]	54 (42–68) [20–92]	0.200^b^
**Fatigue – Baseline Median, (IQR) [Min–Max])**	58 (45–72) [20–95]	56 (42–70) [18–90]	60 (48–75) [25–98]	0.631^b^

Continuous variables are presented as mean ± standard deviation (minimum–maximum) or median (interquartile range [IQR], minimum–maximum), depending on data distribution. Categorical variables are presented as n (%). **^a^** One-way ANOVA. **^b^** Kruskal–Wallis test. **^c^** Pearson’s χ² test (Fisher’s exact test was used when expected cell counts were <5).

**Abbreviations:** ETT, Elastic Therapeutic Taping; PT, Placebo Taping; ST, Sham Taping; SD, Standard Deviation; BMI, Body Mass Index; VAS, Visual Analog Scale; IQR, Interquartile Range; min, minutes.

### Comparison of pain scores (primary outcome)

At 12, 24, and 48 hours, the ETT group showed lower pain scores at rest and during coughing than the placebo and sham groups (Fig 3).

**Fig 3:**
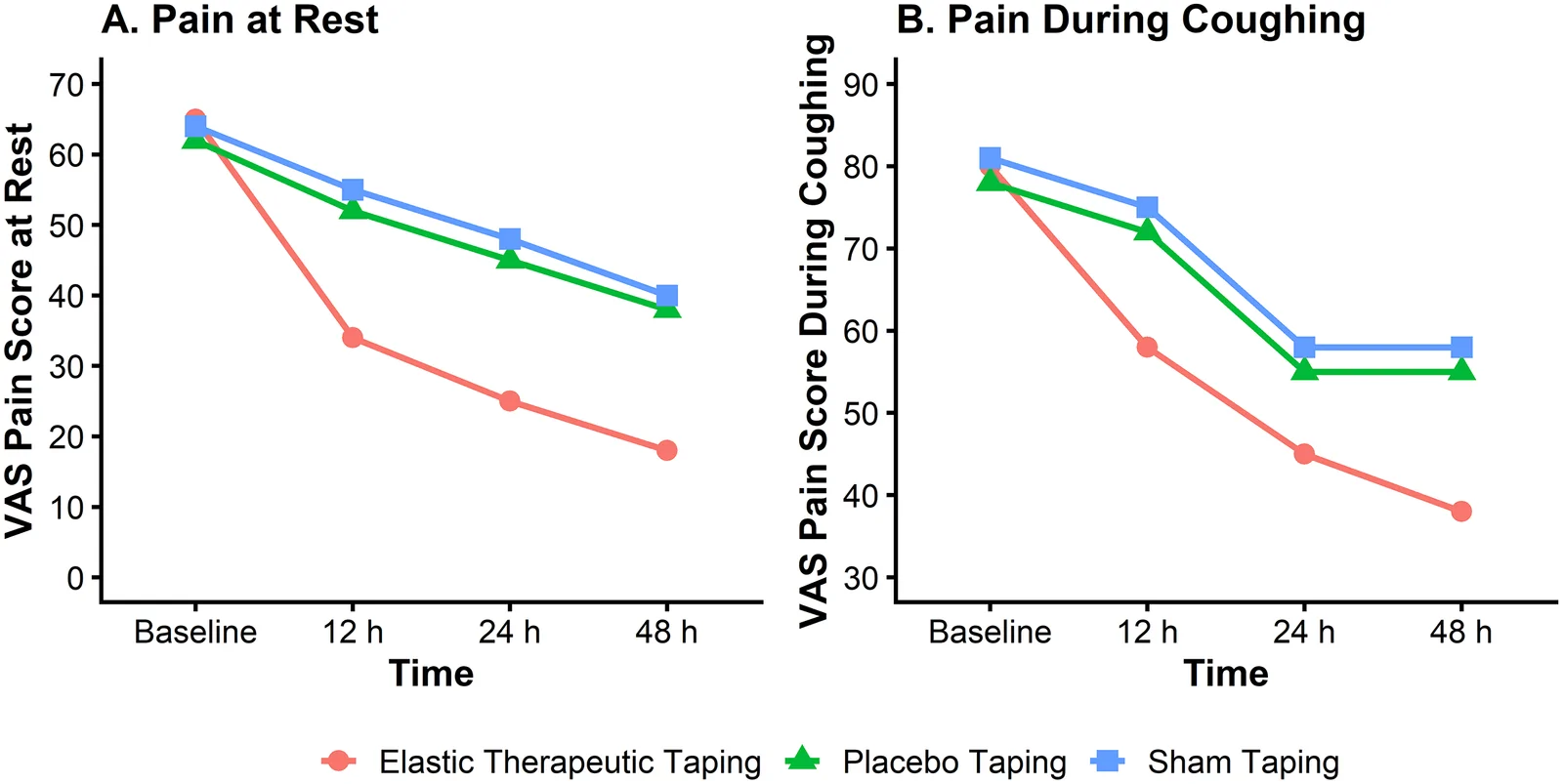
Changes in postoperative pain scores over time. Panel A shows VAS pain scores at rest, and Panel B shows VAS pain scores during coughing at baseline and at 12, 24, and 48 hours after surgery.

Post-hoc analyses showed that pain reduction was driven by the ETT group, with no significant differences between the placebo and sham groups. For pain during coughing, a marked improvement from baseline was observed in the ETT group at 48 hours (Table 2). A significant group × time interaction was found (F(6,384) = 4.12, p = 0.001, partial η² = 0.089).

**Table 2:** Comparison of post-intervention clinical outcomes between the groups.

Clinical Parameters	ETT Group (n = 65)	PT Group (n = 65)	ST Group (n = 65)	p
**Resting Pain (VAS)**				
**Baseline**	65.1 ± 17.6	61.4 ± 17.4	65.2 ± 17.4	0.642
**12 h**	33.9 ± 12.4^a^	51.6 ± 15.6^b^	56.5 ± 15.8^b^	**0.001**
**24 h**	27.1 ± 11.6^a^	44.6 ± 14.0^b^	49.3 ± 16.3^b^	**0.001**
**48 h**	19.0 ± 10.2^a^	37.5 ± 13.7^b^	41.2 ± 16.2^b^	**0.001**
**Pain During Coughing (VAS)**				
**Baseline**	79.7 ± 12.2	76.8 ± 13.6	81.8 ± 11.9	0.585
**12 h**	58.9 ± 17.6^a^	71.4 ± 13.7^b^	74.5 ± 15.0^b^	**0.001**
**24 h**	44.6 ± 14.0^a^	59.8 ± 15.6^b^	65.1 ± 16.8^b^	**0.002**
**48 h**	39.2 ± 17.0^a^	56.3 ± 17.2^b^	59.4 ± 16.8^b^	**0.001**
**SpO_2_ (%)**				
**Baseline**	95 (94–96)	96 (94–97)	95 (94–96)	0.620
**12 h**	96 (95–97)	96 (95–97)	96 (95–97)	0.880
**24 h**	97 (96–99)	96 (95–97)	96 (95–97)	0.710
**48 h**	96 (95–97)	95 (94–98)	97 (96–98)	0.650
**Other Clinical Outcomes**				
**Sleep Quality (48 h, VAS)**	65 (52–78)^a^	45 (35–55)^b^	42 (30–52)^b^	**0.001**
**TUG Test (48 h, s)**	11.2 (9–13)^a^	14.5 (12–17)^b^	15.2 (13–18)^b^	**0.002**
**Length of Stay (days)**	4 (3–5)^a^	5 (4–6)^a,b^	5 (4–7)^b^	**0.042**

Resting pain and pain during coughing are presented as mean ± standard deviation (SD). SpO_2_, sleep quality, TUG, and length of stay are presented as median (interquartile range [IQR]). P values for resting pain and pain during coughing were obtained from the two-way mixed ANOVA. P values for SpO_2_, sleep quality, TUG, and length of stay were obtained using the Kruskal–Wallis test. Different superscript letters (a, b) indicate statistically significant pairwise differences between groups (adjusted p < 0.05). Bonferroni correction was applied following mixed ANOVA, and Dunn–Bonferroni correction following Kruskal–Wallis tests. Bold values indicate statistical significance (p < 0.05).

**Abbreviations:** ETT, Elastic Therapeutic Taping; PT, Placebo Taping; ST, Sham Taping; VAS, Visual Analog Scale; SpO_2_, Oxygen Saturation; TUG, Timed Up and Go; IQR, Interquartile Range.

Between-group comparisons demonstrated clinically meaningful reductions in resting pain in the ETT group compared with both control groups at all postoperative time points. At 12 h, the mean difference was −17.8 mm (95% CI: −22.6 to −12.9) versus placebo and −22.6 mm (95% CI: −27.5 to −17.7) versus sham. At 24 h, the corresponding differences were −17.5 mm (95% CI: −21.9 to −13.0) and −22.1 mm (95% CI: −27.1 to −17.2), respectively. At 48 h, the mean differences were −18.5 mm (95% CI: −22.7 to −14.3) versus placebo and −22.2 mm (95% CI: −26.9 to −17.5) versus sham. All observed between-group differences exceeded the reported minimal clinically important difference for acute postoperative pain.

### Mobility, sleep, and other clinical outcomes

Functional mobility, psychometric scores, and recovery-related outcomes are summarized in Table 2. The ETT group demonstrated the fastest performance on the TUG test at 48 hours (11.2 s), which was significantly better than that of the other two groups (p = 0.002). Compared with the PT group, the median TUG time was 2.2 seconds shorter (95% CI: −2.8 to −0.7), and compared with the ST group, it was 2.6 seconds shorter (95% CI: −3.1 to −1.2). Sleep quality scores were also higher in the ETT group (65/100) compared with the control groups (p = 0.001).

As shown in Table 3, total paracetamol (p = 0.004) and tramadol (p = 0.038) consumption during the first 48 postoperative hours were significantly lower in the ETT group than in the placebo and sham groups. Compared with both control groups, the median difference in tramadol consumption was −50 mg (95% CI: −50 to −50). NSAID use did not differ between groups (p = 0.112).

**Table 3:** Total analgesic consumption during the first 48 postoperative hours.

Analgesic Parameters	ETT Group (n = 65)	PT Group (n = 65)	ST Group (n = 65)	p
**Paracetamol (total mg, mean ± SD)**	2000 ± 500^a^	3000 ± 500^b^	3000 ± 500^b^	**0.004**
**Tramadol (total mg, mean ± SD)**	50 ± 50^a^	100 ± 50^b^	100.0 ± 50.0^b^	**0.038**
**NSAID use, n (%)**	11 (16.9%)	13 (20.0%)	15 (23.0%)	**0.112**

Values are presented as mean ± standard deviation (SD) or n (%), as appropriate. Different superscript letters (a, b) within the same row indicate statistically significant differences between groups according to Bonferroni-adjusted post hoc comparisons (adjusted p < 0.05). Tramadol was administered only as rescue analgesia. Mean tramadol consumption was calculated across all randomized participants, including those who did not require rescue analgesia (recorded as 0 mg). Bold values indicate statistical significance (p < 0.05).

**Abbreviations:** ETT, Elastic Therapeutic Taping; PT, Placebo Taping; ST, Sham Taping; SD, Standard Deviation; NSAID, Nonsteroidal Anti-inflammatory Drug.

Total analgesic consumption was lowest in the ETT group (Fig 4). This was also reflected in a shorter median length of hospital stay in the ETT group (median, 4 days; p = 0.042).

**Fig 4:**
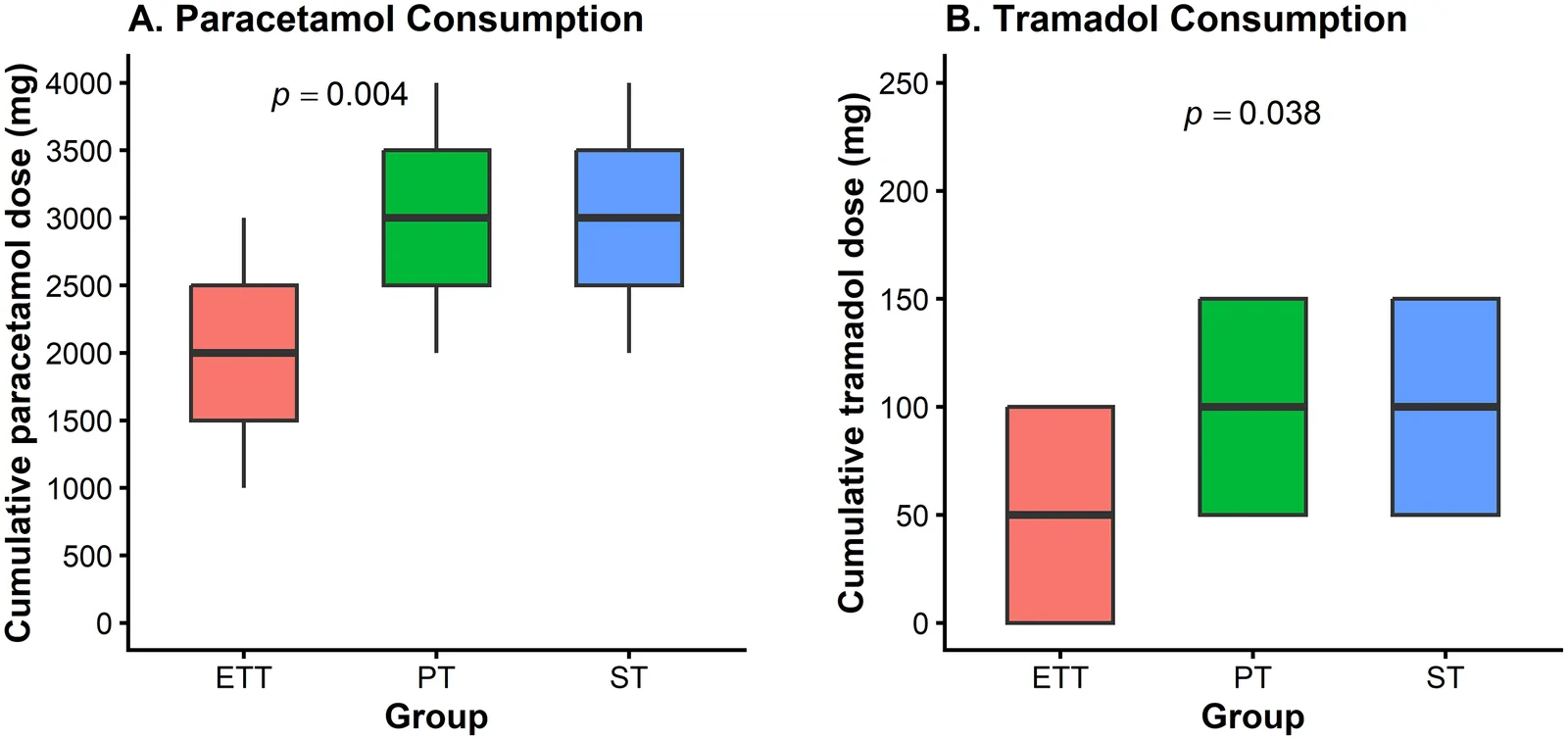
Cumulative analgesic consumption during the first 48 postoperative hours. Panel A shows cumulative paracetamol consumption, and Panel B shows cumulative tramadol consumption. Boxplots represent the median, interquartile range, and whiskers. ETT: Elastic Therapeutic Taping; PT: Placebo Taping; ST: Sham Taping.

### Correlation analysis results

Exploratory correlation analyses were conducted to examine associations between clinical outcomes. Spearman correlation analysis (Table 4) showed a strong positive association between 48-hour VAS pain scores and TUG times (r_s_ = 0.684, p = 0.001), and a strong negative association between pain and sleep quality (r_s_=−0.712, p = 0.001). LOS was positively correlated with both pain intensity (r_s_ = 0.545, p = 0.003) and mobilization speed (r_s_ = 0.612, p < 0.001).

**Table 4:** Correlation between Pain, Mobility, and Clinical Recovery (n = 195).

Variables	VAS Pain (48 h)	TUG Test (48 h)	Sleep Quality	Length of Stay
**VAS Pain (48 h)**	—	0.684 (p < 0.001)	−0.712 (p < 0.001)	0.545 (p = 0.003)
**TUG Test (48 h)**		—	−0.590 (p < 0.001)	0.612 (p < 0.001)
**Sleep Quality**			—	−0.420 (p = 0.012)

Values represent Spearman correlation coefficients. Corresponding p values are shown in parentheses. Bold p values indicate statistical significance (p < 0.05).

**Abbreviations:** VAS, Visual Analog Scale; TUG, Timed Up and Go.

### Overall findings

This 3-arm randomized controlled study demonstrates that ETT significantly reduces post-sternotomy pain at rest and during coughing. The magnitude of pain reduction exceeded the minimal clinically important difference, indicating a clinically meaningful effect. Moreover, reduced opioid requirements in the ETT group support its role as an opioid-sparing adjuvant in the early postoperative period.

#### Pain control and underlying mechanisms.

The analgesic effect of ETT may be explained by both neurophysiological and biomechanical mechanisms [19]. Continuous cutaneous stimulation may enhance mechanoreceptor activation and inhibit nociceptive input [20]. In the present study, the approximately 20–22 mm reduction in VAS scores observed in the ETT group exceeded the reported minimal clinically important difference of 9.9 mm for acute postoperative pain measured on a 100-mm visual analog scale, indicating a clinically meaningful effect [21].

The significant reduction in movement-evoked pain during coughing suggests that ETT may exert effects beyond sensory modulation. After median sternotomy, the soft tissue undergoes micro-movements continuously stimulating nociceptors [1,6]. ETT uses gentle elevation of the skin and low level of tension applied to alter the mechanical properties of the underlying tissue, thus enhancing proprioception and musculofascial stability [22]. It has been proposed that ETT limits micro-movements and reduces mechanically induced nociceptor activation [23]. ETT enhances proprioceptive input, improving neuromuscular control and suppressing functional pain [24]. Although these neurophysiological and biomechanical mechanisms are biologically plausible and supported by previous studies, they were not directly evaluated in the present study. Therefore, they should be considered as potential explanations for the observed clinical outcomes rather than confirmed mechanisms of action.

The lack of significant differences between the placebo and sham groups suggests that the analgesic effects of ETT were not attributable to expectancy alone [25]. This is consistent with previous studies of patients who have undergone sternotomy [4,6]. In contrast, the inconsistent results of other trials may have been attributable to inadequate taping techniques or suboptimal tape placement that limited tape recoil in the surgical setting [26]. Our findings suggest that biomechanical support provided under appropriate tension may contribute to the observed analgesic effect.

Due to the numerous factors contributing to anxiety post-CABG surgery, it is not surprising that only doing a local intervention is not enough to change this systemic stress response. Even if expectations and psychological coping may alter how someone perceives pain (for example, that idea that someone expects to be able to identify a trigger), the fact that there is no difference in anxiety among the groups indicates that the analgesic effect of ETT is more due to neurophysiological and biomechanical mechanisms [6,27]. However, previous work has shown that, despite reductions in physical pain, improvements in quality of life and psychological outcomes after sternotomy may be slower or more limited, indicating that psychological and trust-related processes should still be considered alongside pain recovery [28]. Pain reduction became evident at 12 hours and progressed up to 48 hours, suggesting a cumulative time-dependent effect. This finding is consistent with biomechanical models proposing that ETT redistributes mechanical stress around the surgical site [1,22,23].

Effective early pain control may limit central sensitization and reduce the risk of chronic postoperative pain, although the transition from acute to chronic pain is multifactorial and influenced by several patient- and surgery-related factors [29,30]. While Parreira et al. argued that ETT is not more effective than placebo, the absence of analgesic effects in the sham and tension-free placebo groups in our study contradicts this view and supports a role for mechanoreceptive stimulation and biomechanical support [25]. ETT after sternotomy has also been associated with improved respiratory comfort [6]. Our findings suggest that ETT may preferentially reduce functional pain components triggered by movement rather than pain at rest.

The marked reduction in total analgesic and tramadol consumption in the ETT group suggests that the intervention may not only improve subjective pain perception but also reduce postoperative analgesic requirements [6]. However, in that study, analgesic consumption reached a plateau in the early postoperative period, whereas our findings demonstrate a continued reduction in analgesic requirements up to 48 hours. This progressive effect more strongly supports the role of ETT as an opioid-sparing adjuvant during early recovery.

In clinical conditions with lower mechanical stress, such as musculoskeletal pain, the analgesic effects of taping are often limited or inconsistent. In contrast, the marked mechanical instability and inflammatory load of the chest wall after sternotomy increase the need for external support, which may explain the more pronounced clinical benefit observed in this population [25]. Especially in older individuals undergoing CABG with multiple comorbidities, reduced tramadol requirements represent a clinically meaningful benefit by lowering the risk of opioid-related complications, including delirium, respiratory depression, and gastrointestinal side effects.

Similar NSAID use across groups suggests that ETT preferentially reduces severe, movement-evoked pain requiring opioids rather than baseline inflammatory pain. Although benefits have been reported in musculoskeletal conditions, our findings indicate that this effect is most evident in reduced rescue opioid use [31,32]. Comparable reductions in postoperative pain have also been observed after abdominal surgery [33].

In contrast, in surgical populations with different biomechanical demands, such as total knee arthroplasty, ETT has not shown a clear advantage in reducing analgesic use [34]. This may reflect the mechanically sensitive nature of post-sternotomy pain, which is continuously stressed by breathing and thoracic expansion. These findings align with previous sternotomy studies, while inconsistencies reported in other surgical populations and meta-analyses likely reflect differences in biomechanical demands and taping techniques [6,25,35]. Thus, the observed effect cannot be explained by expectancy alone.

#### Functional improvements and proprioceptive input.

The rapid mobilization and improved TUG performance after ETT suggest that its effects extend beyond nociceptive modulation. This is consistent with evidence showing strong associations between kinesiophobia, pain, disability, and reduced quality of life [36].

Our findings align with this perspective; although kinesiophobia was not directly measured. Objective functional measures such as the TUG capture not only muscular performance but also movement-related fear and self-confidence and are therefore key indicators in postoperative rehabilitation [37]. This is consistent with rehabilitation models showing that physical support enhancing postoperative trunk stability can facilitate mobility [38]. However, subjective confidence does not always correlate with objective walking speed, suggesting that functional improvements may reflect not only perceptual factors but also mechanical and proprioceptive inputs [39]. These correlations should be considered exploratory and hypothesis-generating and require confirmation in future studies.

#### Respiratory patterns and sleep quality: Secondary effect.

Reduced pain with ETT may lessen guarded breathing and promote more effective ventilation, providing a physiological basis for the observed changes in oxygen saturation. After abdominal surgery, Manzano et al. reported only transient SpO_2_ improvements without sustained between-group differences, whereas structural and ventilatory differences after sternotomy may explain the relatively greater changes observed in our study [40]. In addition, routine postoperative oxygen therapy may create a ceiling effect, limiting the detection of true between-group differences in SpO_2_ [41].

Postoperative pain is negatively associated with sleep quality, with higher pain strongly disrupting sleep [42]. Consistent with this relationship, reduced pain and improved sleep quality were observed in our study. ETT may indirectly improve sleep by reducing postoperative pain, which is closely associated with postoperative sleep quality [43,44]. Further studies are needed to determine whether this effect is direct or secondary to pain reduction.

### Implications for practice

#### Clinical implications and safety.

The approximately 1-day reduction in LOS observed in the ETT group suggests potential benefits for clinical recovery and healthcare resource utilization. Because LOS is influenced by multiple factors, including postoperative complications, hemodynamic stability, discharge practices, and functional recovery, this finding should be interpreted with caution. Similar LOS reductions have been demonstrated with early mobilization protocols across surgical populations [45]. Although patients in the ETT group experienced shorter hospital stays, the present study was not designed to determine the independent contribution of ETT to discharge timing. Larger studies with adjustment for relevant clinical covariates are needed to confirm this association.

ETT is a noninvasive intervention with no serious adverse events, supporting its safety as part of multimodal analgesia and rehabilitation [46]. Together, these findings suggest that ETT may provide symptomatic and operational benefits during early postoperative recovery after sternotomy. Larger multicenter studies are needed to confirm these effects.

### Strengths and limitations

Major strengths of this study include its three-arm randomized design with both placebo and sham control groups, a sample size determined by an a priori power analysis, and the comprehensive assessment of pain, mobility, analgesic consumption, and recovery-related outcomes. Nevertheless, several limitations should be acknowledged. The single-center design and short follow-up limit generalizability and long-term inference. In addition, the trial was registered retrospectively, although no changes were made to the study design, outcomes, or analysis plan after participant enrollment began. Functional mobility was measured with the TUG, whereas kinesiophobia and pain-related avoidance were not evaluated with validated scales; thus, inferences regarding fear of movement remain indirect and hypothesis-generating. All taping was performed by a single physiotherapist aware of group allocation, which may introduce provider bias. In addition, use of a single taping material and tension protocol may limit generalizability to other techniques. Despite these limitations, randomization and the use of placebo and sham control groups were implemented to minimize potential bias. The outcome assessments were performed by a physiotherapist who did not administer the intervention. However, the success of participant and assessor blinding was not formally evaluated. In addition, because ETT and sham taping were applied to different anatomical regions, some participants may have inferred their group allocation. Therefore, the possibility of partial unblinding, particularly for subjective outcome measures, cannot be excluded.

## Conclusions

In conclusion, ETT provides clinically meaningful pain reduction after CABG via median sternotomy. The intervention was particularly effective for movement-evoked pain and was associated with reduced analgesic use, improved early mobilization, and a shorter length of hospital stay. Given its noninvasive, low-cost, and safe profile, ETT represents practical adjunct to multimodal postoperative pain management after cardiac surgery. Further multicenter studies with longer follow-up are warranted to confirm long-term clinical and economic benefits.

## Supporting information

S1 Checklist CONSORT 2010 checklist. (PDF)

S2 Checklist PLOS human participants research checklist. (PDF)

S1 Protocol Study protocol (English version). (PDF)

S2 Protocol Original study protocol (Turkish version). (PDF)

## Abbreviations

ANOVA: Analysis of variance
BMI: Body mass index
CABG: Coronary artery bypass grafting
CE: Conformité Européenne (European Conformity)
CONSORT: Consolidated Standards of Reporting Trials
ETT: Elastic therapeutic taping
ICMJE: International Committee of Medical Journal Editors
ICU: Intensive care unit
IQR: Interquartile range
LOCF: Last observation carried forward
LOS: Length of hospital stay
Min–Max: Minimum–Maximum
NSAIDs: Non-steroidal anti-inflammatory drugs
PT: Placebo taping
SD: Standard deviation
SpO_2_: Peripheral oxygen saturation
ST: Sham taping
TUG: Timed Up and Go
VAS: Visual Analog Scale
