# Supplementary material for: Effectiveness of elastic therapeutic taping in pain management after median sternotomy: A randomized controlled trial
Source: PLoS One. 2026 Sep 28;21(9):e0359282. doi: 10.1371/journal.pone.0359282 (PMC13618946; doi:10.1371/journal.pone.0359282)
Supplement: Supplementary file 1 [file pone.0359282.s001.pdf]

The CONSORT reporting checklist

For checking that reports of randomised trials can be understood and used by everyone

> ⓘ Note
>
> If you have not used a reporting guideline before, read about how and why to use them and check whether CONSORT is the most applicable reporting guideline for your work.
>
> Reporting guidelines are most useful when used early in research. When writing a manuscript or application, consider using the Full Guidance where you'll see explanations and examples for each item.
>
> After writing, demonstrate adherence by completing this checklist:
>
> 1. Specify where each item is described (see Note 1).
> 2. Cite this checklist (See Note 2).
> 3. Include your completed checklist as a supplement when submitting to a journal so that future readers can use it to find information.

	Item Description	Location (or reason for not reporting)
**Title and Abstract**		
1a. Title	Identification as a randomised trial.	Title: "Effectiveness of Elastic Therapeutic Taping in Pain Management After Median Sternotomy: A Randomized Controlled Trial" (Page 1)
1b. Structured Abstract	Structured summary of the trial design, methods, results, and conclusions.	Abstract section (Page 2)
**Open Science**		
2. Trial Registration	Name of trial registry, identifying number (with URL) and date of registration.	Methods: Trial registration at ClinicalTrials.gov (NCT06910215); first posted on April 2, 2025 (Page 5).
Protocol and statistical analysis plan	Where the trial protocol and statistical analysis plan can be accessed.	Trial Protocol: Available as Supplementary Material. The Statistical Analysis Plan is described in the Statistics section (Pages 9–10).
4. Data sharing	Where and how the individual de-identified participant data (including data dictionary), statistical code and any other materials can be accessed.	All relevant data underlying the findings of this study are publicly available in the Zenodo
		repository: https://doi.org/10.5281/zenodo.20922725 (Page 24)

5. Funding and Conflicts of Interest		
5a. Funding	Sources of funding and other support (eg, supply of drugs), and role of funders in the design, conduct, analysis, and reporting of the trial.	This study was supported by the Scientific Research Projects Coordination Unit of Bolu Abant Izzet Baysal University (Project No: 2025-BDP-01) (Page 25)
5b. Conflicts of interest	Financial and other conflicts of interest of the manuscript authors.	Conflicts of Interest: All authors declare no conflicts (Page 24)
**Introduction**		
6. Background and rationale	Scientific background and rationale.	Introduction: Paragraphs 1-3 (Page 3-4)
7. Objectives	Specific objectives related to benefits and harms.	Introduction: Study objectives and hypothesis (Page 4).
**Methods**		
8. Patient and public involvement	Details of patient or public involvement in the design, conduct and reporting of the trial.	Not reported
9. Trial Design	Description of trial design including type of trial (eg, parallel group, crossover), allocation ratio, and framework (eg, superiority, equivalence, non-inferiority, exploratory).	Methods: Single-center, randomized, double-blind, placebo- and sham-controlled, 1:1:1 parallel-group superiority trial (Page 5).
10. Changes to trial protocol	Important changes to the trial after it commenced including any outcomes or analyses that were not pre-specified, with reason.	None. The trial was conducted and analyzed as originally planned in the protocol.
11. Trial Setting	Settings (eg, community, hospital) and locations (eg, countries, sites) where the trial was conducted.	Methods: Single-center trial conducted at the Department of Cardiovascular Surgery, Izzet Baysal Training and Research Hospital, Bolu, Turkey (Page 5)
12. Eligibility Criteria		
12a. Participants	Eligibility criteria for participants.	Inclusion/Exclusion Criteria section (Page 5)
12b. Other	If applicable, eligibility criteria for sites and for individuals delivering the interventions (eg, surgeons, physiotherapists).	The interventions were delivered by a certified physiotherapist with more than 10 years of clinical experience. The study was conducted in a specialized cardiovascular surgery department of a
		university hospital.

13. Intervention and comparator	Intervention and comparator with sufficient details to allow replication. If relevant, where additional materials describing the intervention and comparator (eg, intervention manual) can be accessed.	Interventions: Detailed for ETT, PT, and ST groups (Page 6-7)
14. Outcomes	Prespecified primary and secondary outcomes, including the specific measurement variable (eg, systolic blood pressure), analysis metric (eg, change from baseline, final value, time to event), method of aggregation (eg, median, proportion), and time point for each outcome.	Outcome Measures: Primary (VAS pain), Secondary (SpO2, Sleep, Fatigue, TUG, Analgesics) (Page 7-8)
15. Harms	How harms were defined and assessed (eg, systematically, non-systematically).	Methods: Safety monitoring (Page 9). Skin reactions (redness, irritation, itching) and other adverse events were systematically monitored and documented throughout the study.
16. Sample Size		
16a. How sample size was determined	How sample size was determined, including all assumptions supporting the sample size calculation.	Methods: Sample size calculation using G*Power (v3.1.9.7) based on Cohen's d = 0.49, two-sided $\alpha = 0.05$, 80% power, yielding a required sample size of 195 participants (65 per group) (Page 9)
16b. Interim analyses and stopping criteria	Explanation of any interim analyses and stopping guidelines.	Not applicable. No interim analyses or formal stopping guidelines were planned.
17. Randomisation		
17a. Sequence Generation	Who generated the random allocation sequence and the method used.	Methods: Before participant recruitment, the random allocation sequence was generated by the corresponding author using computer-generated block randomization (Blockrand package in R v4.5.2; fixed block size of six) (Page 4).
17b. Type of Randomisation	Type of randomisation and details of any restriction (eg, stratification, blocking, and block size).	Block randomization was used to ensure an equal distribution of participants across the three groups (1:1:1 ratio). The randomization sequence was generated using the 'blockrand' package in R software (version
		4.5.2).

18. Allocation concealment mechanism	Mechanism used to implement the random allocation sequence (eg, central computer/telephone; sequentially numbered, opaque, sealed containers), describing any steps to conceal the sequence until interventions were assigned.	Methods: Allocation concealment was ensured using sequentially numbered, opaque, sealed envelopes containing the pre-generated random allocation sequence. The envelopes were opened by the physiotherapist only after completion of the baseline assessments and immediately before intervention assignment (Page 4).
19. Implementation	Whether the personnel who enrolled and those who assigned participants to the interventions had access to the random allocation sequence.	Methods: Eligible participants were enrolled by the cardiovascular surgery team, who did not have access to the random allocation sequence. The corresponding author generated the random allocation sequence before recruitment, and group assignment was implemented by the physiotherapist using sequentially numbered, opaque, sealed envelopes after completion of the baseline assessments (Page 4).
20. Blinding		
20a. Who was blinded	Who was blinded after assignment to interventions (eg, participants, care providers, outcome assessors, data analysts).	Methods: Participants and outcome assessors were blinded to group allocation. The physiotherapist delivering the intervention was not blinded because of the nature of the intervention and had no role in participant recruitment, outcome assessment, data collection, or statistical analysis (Pages 4 and 7).
20b. How blinding was achieved	If blinded, how blinding was achieved and description of the similarity of interventions.	Methods: Blinding was achieved by using identical elastic therapeutic tapes in all groups. Participants and outcome assessors were blinded to treatment allocation. The physiotherapist

		who applied the interventions was necessarily aware of group assignment because of the nature of the intervention but was not involved in participant recruitment, outcome assessment, data collection, or statistical analysis (Pages 4 and 6–7).
21. Statistical methods		
21a. Comparing groups	Statistical methods used to compare groups for primary and secondary outcomes, including harms.	Methods: Statistical analyses are described in the Statistics section, including one-way ANOVA, Kruskal–Wallis, χ^2/Fisher's exact tests, two-way mixed ANOVA, Friedman test, and Bonferroni-adjusted post hoc analyses. (Pages 9–10)
21b. Definition of who is included in each analysis	Definition of who is included in each analysis (e.g., all randomised participants), and in which group.	Methods/Results: All analyses were conducted using a modified intention-to-treat (mITT) approach. Participants who received the allocated intervention and had evaluable post-intervention outcome data were included in the analysis. A total of 195 participants (65 per group) were included in the modified intention-to-treat analysis according to their original randomized group allocation (Pages 10–11).
21c. Missing Data	How missing data were handled in the analysis.	Methods/Results: Missing outcome data were handled using a modified intention-to-treat (mITT) approach. Participants without evaluable post-intervention outcome data (n = 7) were excluded from the final analysis because outcome data were unavailable (Pages 10–11).
21d. Additional Analyses	Methods for any additional analyses (eg, subgroup and sensitivity analyses), distinguishing pre-specified from post hoc.	Post hoc Bonferroni-adjusted pairwise comparisons and Spearman correlation analyses

		were performed as additional analyses. (Pages 9–10 and 16–17.
22. Participant flow, including flow diagram		
22a. Participant Numbers	For each group, the numbers of participants who were randomly assigned, received intended intervention, and were analysed for the primary outcome.	Results: A total of 203 participants were randomly assigned to the ETT (n = 67), PT (n = 68), or ST (n = 68) groups. All randomized participants received their allocated intervention. A total of 195 participants (65 per group) were included in the primary outcome analysis using the modified intention-to-treat (mITT) approach (Pages 10–11).
22b. Losses and exclusions	For each group, losses and exclusions after randomisation, together with reasons.	Results: Seven participants were lost to follow-up after randomization: 2 in the ETT group (withdrawal at own request), 3 in the PT group (2 early discharge and 1 missing data), and 2 in the ST group (1 withdrawal at own request and 1 clinical deterioration). Details are provided in the CONSORT flow diagram (Figure 1) and the Results section (Pages 10–11).
23. Recruitment		
23a. Dates	Dates defining the periods of recruitment and follow-up for outcomes of benefits and harms.	Methods: Participants were recruited between January 22, 2025, and December 24, 2025. Follow-up for the primary and secondary outcomes (pain, oxygen saturation, sleep quality, functional mobility, analgesic use, and adverse events) was conducted for up to 48 hours post-intervention, and length of hospital stay was monitored until discharge (Pages 5–9).
23b. Reasons for stopping	If relevant, why the trial ended or was stopped.	ethods: The trial ended as planned after the

recruitment		predetermined sample size of 195 participants who completed the study was achieved. No premature termination or stopping occurred (Page 9).
24. Intervention and comparator delivery		
24a. As Administered	Intervention and comparator as they were actually administered (eg, where appropriate, who delivered the intervention/comparator, whether participants adhered, whether they were delivered as intended (fidelity)).	Methods: The interventions and comparators were administered as planned by a certified physiotherapist with more than 10 years of experience. Standardized taping protocols were used for all three groups (ETT, PT, and ST), and all interventions were delivered according to predefined protocols (Pages 6–7).
24b. Concomitant Care	Concomitant care received during the trial for each group.	Methods: All participants received the same standard postoperative care, including the routine analgesia protocol. Intravenous paracetamol was administered according to the standard postoperative protocol, rescue tramadol (50 mg) was administered when VAS ≥40 mm, and NSAID use was at the discretion of the treating physician (Page 8).
25. Baseline Data	A table showing baseline demographic and clinical characteristics for each group.	Results: Baseline demographic, surgical, and clinical characteristics of the participants are presented in Table 1, including age, BMI, sex, smoking status, operative variables, and baseline clinical outcome measures (Table 1, Pages 11–12).
26. Numbers analysed, outcomes, and estimation	For each primary and secondary outcome, by group:<br>• the number of participants included in the analysis.<br>• the number of participants with available data	Results: The number of participants included in each analysis (n = 65 per group) and the results for all primary and secondary outcomes are

	at the outcome time point.<br>• result for each group, and the estimated effect size and its precision (such as 95% confidence interval).<br>• for binary outcomes, presentation of both absolute and relative effect size.	presented in Tables 2 and 3. Continuous outcomes are reported as median (IQR) or mean ± SD, as appropriate. Between-group effect estimates with 95% confidence intervals are reported for the primary outcome and selected secondary outcomes, together with corresponding p values (Tables 2–3, Pages 13–17).
27. Harms	All harms or unintended events in each group.	Results: No serious adverse events occurred during the study. Two participants in the ETT group experienced mild skin redness, and one participant in the PT group reported mild itching. No adverse events were observed in the ST group. None of these events required medical treatment, resulted in premature tape removal, or led to study withdrawal (Page 9).
28. Ancillary Analyses	Any other analyses performed, including subgroup and sensitivity analyses, distinguishing pre-specified from post hoc.	Results: Additional analyses included Spearman correlation analyses to evaluate the relationships between pain intensity, functional mobility, sleep quality, and length of hospital stay. Bonferroni-adjusted post hoc comparisons were performed, where appropriate, to identify pairwise differences between treatment groups (Pages 10 and 17).
**Discussion**		
29. Interpretation	Interpretation consistent with results, balancing benefits and harms, and considering other relevant evidence.	Discussion: The findings indicate that elastic therapeutic taping reduced postoperative pain, improved functional recovery, and decreased analgesic requirements compared with the control groups. No serious adverse events occurred,

		although mild transient skin reactions were observed in a small number of participants. These findings are interpreted by balancing the observed benefits and safety profile of the intervention and are discussed in the context of previous evidence (Pages 18–23).
30. Limitations	Trial limitations, addressing sources of potential bias, imprecision, generalisability, and, if relevant, multiplicity of analyses.	Discussion: The study has several limitations. First, follow-up was limited to the first 48 hours postoperatively; therefore, long-term effects of taping could not be evaluated. Second, this was a single-center study, which may limit the generalizability of the findings. Third, the physiotherapist delivering the intervention could not be blinded because of the nature of the intervention. In addition, hospital discharge criteria were not fully standardized, which may have influenced the length-of-stay outcome (Pages 22–23).

1 How to specify where content is

Tell the reader where they can find information. E.g.,

- Results; paragraph 2
- Methods, Participants; paragraphs 1 & 2.
- Table 3
- Supplement B, para. 4

If you have chosen not to describe an item, explain why. You can do this in the checklist, or as a note below it.

You can describe items in the article body, or in tables, figures, or supplementary materials, and should prioritize items you feel are most important to your intended audience. The order of items in your manuscript does not need to match the order of items in this checklist. You can decide how best to structure your work.

2 How to cite

Describe how you used CONSORT at the end of your Methods section, referencing the resources you used e.g.,

‘We used the CONSORT reporting guideline(1) to draft this manuscript, and the CONSORT reporting checklist(2) when editing, included in supplement A’

If you use a reporting checklist, remember to include it as a supplement when publishing so that readers can easily find information and see how you have interpreted the guidance.

1. Hopewell S, Chan AW, Collins GS, Hróbjartsson A, Moher D, Schulz KF, et al. CONSORT 2025 statement: Updated guideline for reporting randomised trials. The BMJ [Internet]. 2025 Apr;389:e081123. Available from: https://www.ncbi.nlm.nih.gov/pmc/articles/PMC11995449/

2. Hopewell S, Chan AW, Collins GS, Hróbjartsson A, Moher D, Schulz KF, et al. The CONSORT reporting checklist. In: Harwood J, Albury C, Beyer J de, Schlüssel M, Collins G, editors. The EQUATOR network reporting guideline platform [Internet]. The UK EQUATOR Centre; 2025. Available from: https:/resources.equator-network.org/reporting-guidelines/consort/consort-checklist.docx
