# Supplementary material for: Effectiveness of elastic therapeutic taping in pain management after median sternotomy: A randomized controlled trial
Source: PLoS One. 2026 Sep 28;21(9):e0359282. doi: 10.1371/journal.pone.0359282 (PMC13618946; doi:10.1371/journal.pone.0359282)
Supplement: Supplementary file 3 [file pone.0359282.s003.pdf]

Title

The Effectiveness of Elastic Therapeutic Taping in Pain Management After Sternotomy: A Randomized Controlled Double-Blind Study in Patients Undergoing Artery Bypass Grafting Surgery

1. Background

Median sternotomy, commonly performed during coronary artery bypass grafting (CABG), is associated with significant postoperative pain that can adversely affect respiratory function, early mobilization, and overall recovery. Inadequate pain control may lead to shallow breathing, impaired cough effectiveness, increased risk of pulmonary complications, prolonged hospital stay, and delayed functional recovery.

Effective postoperative pain management is therefore essential to enhance patient outcomes and accelerate rehabilitation. Although pharmacological analgesia remains the standard approach, it is often associated with adverse effects such as nausea, vomiting, sedation, and respiratory depression, which may further complicate recovery in cardiac surgery patients. These limitations highlight the need for safe, non-pharmacological adjuncts in postoperative pain management.

Elastic therapeutic taping is a non-invasive intervention increasingly used in musculoskeletal and rehabilitation settings. It is proposed to reduce pain through stimulation of cutaneous mechanoreceptors, improvement of local circulation, reduction of edema, and enhancement of proprioceptive input. While previous studies have suggested potential benefits of elastic therapeutic taping in postoperative settings, existing evidence is limited by small sample sizes, insufficient blinding, and lack of appropriate placebo or sham-controlled designs.

Therefore, there remains a need for well-designed, adequately powered randomized controlled trials to clarify the clinical effectiveness of elastic therapeutic taping following median sternotomy. The present study aims to evaluate the effectiveness of elastic therapeutic taping on postoperative pain and recovery outcomes in patients undergoing CABG using a randomized, double-blind, placebo- and sham-controlled desig.

2. Objective

The primary aim of this study is to evaluate the effectiveness of elastic therapeutic taping in reducing postoperative pain at rest and during coughing in patients undergoing coronary artery bypass grafting (CABG) via median sternotomy.

The secondary aim is to investigate the effects of elastic therapeutic taping on postoperative recovery outcomes, including oxygen saturation (SpO_2), sleep quality, anxiety levels, functional mobility (Timed Up and Go test), analgesic consumption, fatigue, and length of hospital stay.

3. Study Design

This study was designed as a randomized, double-blind, placebo- and sham-controlled, single-center clinical trial with a parallel-group structure. Participants were randomly allocated in a 1:1:1 ratio into three groups: an elastic therapeutic taping group, a placebo taping group, and a

sham (psychosocial support) group. Sample size calculation was performed a priori using G*Power software (v3.1.9.7). Based on the pooled effect size for postoperative pain reduction after median sternotomy reported by King et al., an effect size of Cohen's d = 0.49 was assumed. With a two-sided $\alpha = 0.05$ and 80% power ($1 - \beta = 0.80$), the minimum required sample size was calculated as 65 participants per group, resulting in a total sample size of 195 patients.

4. Participants

Inclusion Criteria

- Age between 18 and 75 years
- Undergoing elective coronary artery bypass grafting (CABG) via median sternotomy
- Able to understand and communicate effectively
- Willing to provide written informed consent

Exclusion Criteria

- Presence of dermatological conditions or open wounds in the sternotomy area
- History of allergy to elastic therapeutic tape or adhesive materials
- Presence of neurological or psychiatric disorders that may affect pain perception
- Use of medications that significantly alter pain sensitivity
- Participation in another interventional clinical study within the past 30 days
- Acute renal or hepatic failure
- Preoperative mechanical ventilation
- Planned reoperation or revision surgery

5. Randomization and Blinding

Participants were randomly allocated in a 1:1:1 ratio to one of the three study groups using a computer-generated block randomization method (Blockrand package, R software v4.5.2). Allocation concealment was ensured using sealed, opaque, and sequentially numbered envelopes prepared by an independent researcher not involved in participant recruitment or assessment. This study employed a double-blind design. Participants, outcome assessors, and data analysts were blinded to group allocation. The physiotherapist responsible for applying the taping procedures was aware of group assignment but was not involved in outcome assessment or data analysis to preserve blinding integrity. No changes were made to the trial design, eligibility criteria, interventions, or outcome measures after trial commencement.

6. Interventions

Elastic Therapeutic Taping Group

Elastic therapeutic tape (Kinesio® Tex Gold, Kinesio Holding Corporation, USA) was applied bilaterally approximately 3 cm lateral to the sternotomy incision in a craniocaudal direction along the anterior thoracic wall. Applications were performed using standardized kinesio taping techniques with 25–35% tension, with the ends applied without tension and the incision area avoided. All procedures followed a predefined anatomical template. The intervention was applied within 6–8 hours after transfer from the intensive care unit to the ward and maintained for up to 48 hours unless removal was required due to adverse reactions.

Placebo Taping Group

The same tape was applied to the same anatomical regions without stretch and without following therapeutic taping principles. This approach was used to mimic the visual and tactile characteristics of the intervention without providing therapeutic effects. The tape remained in place for up to 48 hours.

Sham (Psychosocial Control) Group

Tape was applied without tension to anatomically unrelated regions (e.g., scapular or paraspinal areas) using a standardized application template and contact duration. This condition was designed to control for contextual and psychosocial effects associated with therapist contact. The tape remained in place for up to 48 hours.

Standard Care

All participants received the same standard postoperative analgesia protocol.

7. Outcome Measures

Primary Outcome

Pain intensity at rest and during coughing (Visual Analog Scale, VAS): Pain will be assessed using a 100 mm visual analog scale, where 0 indicates no pain and 100 indicates the worst imaginable pain. Measurements will be obtained at baseline and at 12, 24, and 48 hours post-intervention.

Secondary Outcomes

Oxygen saturation (SpO_2): Peripheral oxygen saturation will be measured using a pulse oximeter, and values will be calculated as the average of three consecutive measurements.

Sleep quality (VAS): Participants will rate their perceived sleep quality using a visual analog scale.

Anxiety level (VAS): Anxiety will be assessed using a visual analog scale.

Functional mobility (Timed Up and Go Test, TUG): Functional mobility will be evaluated by measuring the time required to complete the TUG test.

Analgesic use: Total analgesic consumption (paracetamol, non-steroidal anti-inflammatory drugs, and tramadol) will be recorded cumulatively during the postoperative period.

Length of hospital stay: Duration from surgery to hospital discharge will be documented.

Fatigue (VAS): Fatigue levels will be assessed using a visual analog scale.

8. Data Collection Timeline

Outcome measures were assessed at predefined time points to evaluate changes over the early postoperative period. Baseline measurements were obtained 6–8 hours after transfer from the intensive care unit to the ward, prior to intervention.

Follow-up assessments were conducted at 12, 24, and 48 hours after the intervention. Pain intensity (at rest and during coughing) and oxygen saturation (SpO_2) were assessed at all time points.

Sleep quality, fatigue, anxiety, and functional mobility (Timed Up and Go test) were assessed at 48 hours. Analgesic consumption was recorded cumulatively over the 48-hour postoperative period, and length of hospital stay was documented at discharge.

9. Statistical Analysis

All statistical analyses were performed according to the intention-to-treat principle using SPSS (v20.0) and R software. Descriptive statistics were used to summarize baseline characteristics. Normality and homogeneity of variance were assessed using the Shapiro–Wilk and Levene's tests, respectively.

The primary outcome (pain intensity at rest and during coughing) was analyzed using a two-way mixed-effects ANOVA (group × time), followed by Bonferroni-adjusted post-hoc comparisons when appropriate. For secondary outcomes, oxygen saturation (SpO_2) was analyzed using the Friedman test. Functional mobility (Timed Up and Go test), sleep quality, fatigue, and anxiety were analyzed using the Kruskal–Wallis test. Length of hospital stay was evaluated as a prespecified secondary outcome and was not included in multiple comparison corrections.

Effect sizes were reported as partial eta squared (η^2) for ANOVA and Cohen's d for pairwise comparisons. A two-tailed p value < 0.05 was considered statistically significant. Missing data were handled using the last observation carried forward (LOCF) method, and sensitivity analyses were performed to confirm the robustness of the findings.

10. Ethics

This study was approved by the Bolu Abant Izzet Baysal University Non-Interventional Clinical Research Ethics Committee (Decision No: 2025/07, Date: 21.01.2025). The study protocol was conducted in accordance with the principles of the Declaration of Helsinki. All participants were informed about the study's purpose and procedures, and written informed consent was obtained from all subjects prior to their inclusion in the trial.
