# Supplementary material for: Effectiveness of elastic therapeutic taping in pain management after median sternotomy: A randomized controlled trial
Source: PLoS One. 2026 Sep 28;21(9):e0359282. doi: 10.1371/journal.pone.0359282 (PMC13618946; doi:10.1371/journal.pone.0359282)
Supplement: Supplementary file 4 [file pone.0359282.s004.pdf]

Başlık

Sternotomi Sonrası Ağrı Yönetiminde Elastik Terapötik Bantlamanın Etkinliği: Koroner Arter Bypass Greft Ameliyatı Geçiren Hastalarda Randomize Kontrollü Çift Kör Bir Çalışma

1.Giriş

Koroner arter baypas greftleme (CABG) sırasında yaygın olarak uygulanan median sternotomi, solunum fonksiyonu, erken mobilizasyon ve genel iyileşme sürecini olumsuz etkileyebilen belirgin postoperatif ağrı ile ilişkilidir. Yetersiz ağrı kontrolü; yüzeyel solunuma, öksürük etkinliğinin azalmasına, pulmoner komplikasyon riskinin artmasına, hastanede kalış süresinin uzamasına ve fonksiyonel iyileşmenin gecikmesine yol açabilir.

Bu nedenle, hasta sonuçlarını iyileştirmek ve rehabilitasyonu hızlandırmak için etkili postoperatif ağrı yönetimi büyük önem taşımaktadır. Farmakolojik analjezi standart yaklaşım olmaya devam etmekle birlikte, bulantı, kusma, sedasyon ve solunum depresyonu gibi yan etkilerle ilişkilidir ve bu durum özellikle kardiyak cerrahi hastalarında iyileşme sürecini daha da karmaşık hale getirebilir. Bu sınırlılıklar, postoperatif ağrı yönetiminde güvenli ve non-farmakolojik tamamlayıcı yaklaşımlara olan ihtiyacı ortaya koymaktadır.

Elastik terapötik bantlama, kas-iskelet sistemi ve rehabilitasyon alanlarında giderek daha fazla kullanılan non-invaziv bir müdahaledir. Bu yöntemin; kutanöz mekanoreseptörlerin uyarılması, lokal dolaşımın iyileştirilmesi, ödemin azaltılması ve proprioseptif girdinin artırılması yoluyla ağrıyı azaltabileceği öne sürülmektedir. Önceki çalışmalar elastik terapötik bantlamanın postoperatif dönemde potansiyel faydalar sağlayabileceğini göstermiş olsa da, mevcut kanıtlar küçük örneklem büyüklükleri, yetersiz körleme ve uygun plasebo veya sham kontrollü tasarımların eksikliği gibi sınırlılıklar içermektedir.

Bu nedenle, median sternotomi sonrası elastik terapötik bantlamanın klinik etkinliğini netleştirmek için iyi tasarlanmış ve yeterli örneklem gücüne sahip randomize kontrollü çalışmalara ihtiyaç vardır. Bu çalışma, CABG uygulanan hastalarda postoperatif ağrı ve iyileşme sonuçları üzerine elastik terapötik bantlamanın etkinliğini, randomize, çift kör, plasebo ve sham kontrollü bir tasarım kullanarak değerlendirmeyi amaçlamaktadır.

2. Amaç

Bu çalışmanın birincil amacı, median sternotomi yoluyla koroner arter baypas greftleme (CABG) uygulanan hastalarda elastik terapötik bantlamanın istirahatte ve öksürme sırasında postoperatif ağrıyı azaltmadaki etkinliğini değerlendirmektir.

İkincil amaç ise elastik terapötik bantlamanın postoperatif iyileşme sonuçları üzerindeki etkilerini incelemektir. Bu kapsamda; oksijen satürasyonu (SpO_2), uyku kalitesi, anksiyete düzeyi, fonksiyonel mobilite (Timed Up and Go testi), analjezik tüketimi, yorgunluk ve hastanede kalış süresi değerlendirilecektir.

3. Çalışma Tasarımı

Bu çalışma, randomize, çift kör, plasebo ve sham kontrollü, tek merkezli, paralel grup yapısına sahip bir klinik çalışma olarak tasarlanmıştır. Katılımcılar, 1:1:1 oranında rastgele üç gruba

atanmıştır: elastik terapötik bantlama grubu, plasebo bantlama grubu ve sham (psikososyal destek) grubu. Örneklem büyüklüğü hesaplaması, G*Power yazılımı (v3.1.9.7) kullanılarak a priori olarak gerçekleştirilmiştir. King ve ark. tarafından median sternotomi sonrası postoperatif ağrı azalmasına ilişkin bildirilen birleşik etki büyüklüğüne dayanarak Cohen's d = 0.49 etki büyüklüğü varsayılmıştır. İki yönlü $\alpha = 0.05$ ve %80 güç ($1 - \beta = 0.80$) ile her grup için gerekli minimum örneklem büyüklüğü 65 katılımcı olarak hesaplanmış, toplam örneklem büyüklüğü 195 hasta olarak belirlenmiştir.

4. Katılımcılar

Dahil Edilme Kriterleri

• 18–75 yaş arasında olmak

• Median sternotomi yoluyla elektif koroner arter baypas greftleme (CABG) ameliyatı geçirecek olmak

• Etkili şekilde anlayabilme ve iletişim kurabilme

• Yazılı bilgilendirilmiş onam vermeye istekli olmak

Dışlanma Kriterleri

• Sternotomi bölgesinde dermatolojik hastalık veya açık yara bulunması

• Elastik terapötik bant veya yapışkan materyallere karşı alerji öyküsü

• Ağrı algısını etkileyebilecek nörolojik veya psikiyatrik hastalık varlığı

• Ağrı duyarlılığını belirgin şekilde değiştiren ilaç kullanımı

• Son 30 gün içinde başka bir girişimsel klinik çalışmaya katılım

• Akut böbrek veya karaciğer yetmezliği

• Preoperatif mekanik ventilasyon uygulanmış olması

• Planlı reoperasyon veya revizyon cerrahisi varlığı

5. Randomizasyon ve Körleme

Katılımcılar, bilgisayar tarafından oluşturulan blok randomizasyon yöntemi (R yazılımında Blockrand paketi v4.5.2) kullanılarak 1:1:1 oranında üç çalışma grubundan birine rastgele atanmıştır. Atama gizliliği, katılımcı alımı veya değerlendirmesinde yer almayan bağımsız bir araştırmacı tarafından hazırlanan, kapalı, opak ve sıralı numaralandırılmış zarflar kullanılarak sağlanmıştır. Bu çalışma çift kör tasarımda yürütülmüştür. Katılımcılar, sonuç değerlendiricileri ve veri analistleri grup atamalarından habersiz tutulmuştur. Bantlama uygulamalarından sorumlu fizyoterapist grup atamasını bilmekle birlikte, körlüğün korunması amacıyla sonuç değerlendirmesi veya veri analizine dahil edilmemiştir. Çalışma başladıktan sonra çalışma tasarımında, dahil edilme kriterlerinde, müdahalelerde veya sonuç ölçütlerinde herhangi bir değişiklik yapılmamıştır.

6. Müdahaleler

Elastik Terapötik Bantlama Grubu

Elastik terapötik bant (Kinesio® Tex Gold, Kinesio Holding Corporation, ABD), sternotomi insizyonunun yaklaşık 3 cm lateraline, anterior torasik duvar boyunca kraniokaudal yönde bilateral olarak uygulanmıştır. Uygulamalar, uç kısımlar gerimsiz olacak şekilde ve insizyon bölgesinden kaçınılarak, %25–35 gerim ile standart kinezyo bantlama teknikleri kullanılarak gerçekleştirilmiştir. Tüm uygulamalar önceden belirlenmiş anatomik bir şablona uygun olarak yapılmıştır. Müdahale, yoğun bakım ünitesinden servise transferden sonraki 6–8 saat içinde uygulanmış ve advers reaksiyon gelişmesi durumunda çıkarılmak üzere en fazla 48 saat süreyle yerinde tutulmuştur.

Plasebo Bantlama Grubu

Aynı bant, aynı anatomik bölgelere herhangi bir gerim uygulanmadan ve terapötik bantlama prensipleri izlenmeden uygulanmıştır. Bu yaklaşım, terapötik etki sağlamadan müdahalenin görsel ve dokunsal özelliklerini taklit etmek amacıyla kullanılmıştır. Bant en fazla 48 saat süreyle yerinde tutulmuştur.

Sham (Psikososyal Kontrol) Grubu

Bant, anatomik olarak ilişkili olmayan bölgelere (örneğin skapular veya paraspinal alanlar), gerim uygulanmadan ve standart bir uygulama şablonu ile belirli bir temas süresi sağlanarak uygulanmıştır. Bu koşul, terapist temasına bağlı bağlamsal ve psikososyal etkileri kontrol etmek amacıyla tasarlanmıştır. Bant en fazla 48 saat süreyle yerinde tutulmuştur.

Standart Bakım

Tüm katılımcılara aynı standart postoperatif analjezi protokolü uygulanmıştır.

7. Sonuç Ölçütleri

Birincil Sonuç

İstirahatte ve öksürme sırasında ağrı şiddeti (Görsel Analog Skala, VAS): Ağrı, 0'ın "ağrı yok" ve 100'ün "hayal edilebilecek en şiddetli ağrı"yı temsil ettiği 100 mm'lik görsel analog skala kullanılarak değerlendirilecektir. Ölçümler başlangıçta ve müdahale sonrası 12., 24. ve 48. saatlerde yapılacaktır.

İkincil Sonuçlar

Oksijen satürasyonu (SpO_2): Periferik oksijen satürasyonu pulse oksimetre ile ölçülecek ve değerler ardışık üç ölçümün ortalaması olarak hesaplanacaktır.

Uyku kalitesi (VAS): Katılımcılar algıladıkları uyku kalitesini görsel analog skala ile değerlendirecektir.

Anksiyete düzeyi (VAS): Anksiyete görsel analog skala ile değerlendirilecektir.

Fonksiyonel mobilite (Timed Up and Go Testi, TUG): Fonksiyonel mobilite, TUG testini tamamlama süresi ölçülerek değerlendirilecektir.

Analjezik kullanımı: Postoperatif dönemde toplam analjezik tüketimi (parasetamol, non-steroid antiinflamatuvar ilaçlar ve tramadol) kümülatif olarak kaydedilecektir.

Hastanede kalış süresi: Cerrahiden taburculuğa kadar geçen süre kaydedilecektir.

Yorgunluk (VAS): Yorgunluk düzeyi görsel analog skala ile değerlendirilecektir.

8. Veri Toplama Zaman Çizelgesi

Sonuç ölçütleri, erken postoperatif dönemdeki değişimleri değerlendirmek amacıyla önceden belirlenmiş zaman noktalarında ölçülmüştür. Başlangıç ölçümleri, yoğun bakım ünitesinden servise transferden 6-8 saat sonra ve müdahale öncesinde alınmıştır.

Takip değerlendirmeleri müdahale sonrası 12., 24. ve 48. saatlerde yapılmıştır. Ağrı şiddeti (istirahatte ve öksürme sırasında) ve oksijen satürasyonu (SpO_2) tüm zaman noktalarında değerlendirilmiştir.

Uyku kalitesi, yorgunluk, anksiyete ve fonksiyonel mobilite (Timed Up and Go testi) 48. saatte değerlendirilmiştir. Analjezik tüketimi postoperatif 48 saat boyunca kümülatif olarak kaydedilmiş, hastanede kalış süresi ise taburculuk sırasında belgelendirilmiştir.

9. İstatistiksel Analiz (Statistical Analysis)

Tüm istatistiksel analizler, niyet edilen tedavi (intention-to-treat) prensibine göre SPSS (v 20.0) ve R yazılımı kullanılarak gerçekleştirilmiştir. Tanımlayıcı istatistikler, başlangıç özelliklerini özetlemek amacıyla kullanılmıştır. Normallik ve varyans homojenliği sırasıyla Shapiro-Wilk ve Levene testleri ile değerlendirilmiştir.

Birincil sonuç (istirahatte ve öksürme sırasında ağrı şiddeti), iki yönlü karma etkili ANOVA (grup × zaman) ile analiz edilmiş, gerekli durumlarda Bonferroni düzeltmeli post-hoc karşılaştırmalar yapılmıştır. İkincil sonuçlar için oksijen satürasyonu (SpO_2) Friedman testi ile analiz edilmiştir. Fonksiyonel mobilite (Timed Up and Go testi), uyku kalitesi, yorgunluk ve anksiyete Kruskal-Wallis testi ile değerlendirilmiştir. Hastanede kalış süresi önceden belirlenmiş ikincil bir sonuç olarak ele alınmış ve çoklu karşılaştırma düzeltmelerine dahil edilmemiştir.

Etki büyüklükleri ANOVA için kısmi eta kare (η^2), ikili karşılaştırmalar için Cohen's d olarak raporlanmıştır. İki yönlü $p < 0.05$ değeri istatistiksel olarak anlamlı kabul edilmiştir. Eksik veriler son gözlemin taşınması (LOCF) yöntemi ile ele alınmış ve bulguların sağlamlığını doğrulamak amacıyla duyarlılık analizleri gerçekleştirilmiştir.

10. Etik

Bu çalışma, Bolu Abant İzzet Baysal Üniversitesi Girişimsel Olmayan Klinik Araştırmalar Etik Kurulu tarafından onaylanmıştır (Karar No: 2025/07, Tarih: 21.01.2025). Çalışma protokolü, Helsinki Bildirgesi ilkelerine uygun olarak yürütülmüştür. Tüm katılımcılar çalışmanın amacı

ve prosedürleri hakkında bilgilendirilmiş ve çalışmaya dahil edilmeden önce tüm katılımcılardan yazılı bilgilendirilmiş onam alınmıştır.
