# Supplementary material for: Effectiveness of elastic therapeutic taping in pain management after median sternotomy: A randomized controlled trial
Source: PLoS One. 2026 Sep 28;21(9):e0359282. doi: 10.1371/journal.pone.0359282 (PMC13618946; doi:10.1371/journal.pone.0359282)
Supplement: Supplementary file 5 [file pone.0359282.s005.docx]

**Peer Review Report**

**Manuscript ID: PONE-D-26-16084**

**Title: Effectiveness of Elastic Therapeutic Taping in Pain Management After Median Sternotomy: A Randomized Controlled Trial**

**Reviewer Specialty: Physical Medicine and Rehabilitation**

**Target Journal: PLOS ONE**

**1. ORIGINALITY**

The study addresses a clinically relevant gap in the literature on non-pharmacological pain management following coronary artery bypass grafting (CABG) via median sternotomy. Prior work in this area, most notably Brockmann & Klein (2018) and Temel Aksu et al. (2023), has been limited by small sample sizes, absence of sham controls, and inadequate blinding. The authors correctly identify these methodological deficiencies and position their trial as the first adequately powered, double-blind, placebo- and sham-controlled RCT examining elastic therapeutic taping (ETT) specifically in the CABG/sternotomy population, with a comprehensive outcome battery that includes functional mobility (TUG), sleep quality, opioid consumption, and length of stay (LOS).

The originality claim is largely credible. The inclusion of both a tension-free placebo arm and an anatomically unrelated sham arm represents a genuine methodological advance in the physical therapy trial literature and is directly responsive to concerns raised by the CONSORT nonpharmacological extension and the CoPPS Statement (references 8 and 9). The research question is therefore both timely and scientifically justified.

**Assessment: Acceptable.** The study presents original research not previously published in this form.

**2. TECHNICAL STANDARD**

**2a. Study Design**

The parallel-group, three-arm RCT design is appropriate for the research question. Computer-generated block randomization (Blockrand, R v4.5.2) with 1:1:1 allocation is correctly described, and the blinding structure, participants and outcome assessors blinded, treating physiotherapist unblinded, is standard and honestly reported. The acknowledgment that single-physiotherapist delivery introduces provider bias (noted under Limitations) reflects appropriate transparency.

However, several methodological concerns require attention:

**Block size disclosure:** The manuscript states that block randomization was used but does not specify the block sizes. In a trial where the treating physiotherapist is unblinded and the same person performs all three interventions, undisclosed block sizes raise the theoretical risk of allocation prediction. This should be clarified or the block sizes confirmed as variable.

**Blinding verification:** No formal assessment of blinding success is reported. In physical intervention trials, participant blinding is notoriously difficult to maintain, and the CONSORT nonpharmacological extension explicitly recommends reporting whether blinding was assessed. The authors should have administered a blinding integrity check (e.g., Bang's Blinding Index) post-intervention and report the results. Its complete absence is a significant omission.

**Intervention standardization:** The ETT application protocol is described adequately in broad terms (Kinesio® Tex Gold, bilateral paraincisional placement 3 cm lateral to sternotomy, craniocaudal direction, 25–35% tension, 5 cm wide tape, ends without tension). However, from a PM&R specialist's perspective, several details critical for reproducibility are missing or underspecified:

- The number of tape strips applied per patient is not stated.
- The total length of each strip is not specified.
- Whether skin preparation (cleaning, shaving) was performed prior to taping is not described.

The tension range of 25–35% is stated but the method used to verify or standardize this tension in clinical practice is not explained. Tension in kinesio taping is highly operator-dependent, and without a described calibration method, reproducibility is questionable.

The frequency of tape reapplication (or confirmation that the tape was applied once for the full 48-hour observation window) is not explicitly stated.

Placebo arm construct validity: The placebo arm (same tape, same location, zero tension) is methodologically sound and well-chosen. The sham arm (tape to scapular/paraspinal region) is appropriate for controlling therapist contact time. The authors should clarify whether the total contact time between the physiotherapist and participants was equalized across all three groups, as differential contact time could introduce attention bias.

**2b. Outcome Measures**

**Primary outcome** VAS: The 100-mm Visual Analog Scale for pain at rest and during coughing is a validated, well-established primary outcome for acute postoperative pain and is appropriate for this population. The choice of time points (baseline, 12h, 24h, 48h) is clinically reasonable given the post-ICU transfer context.

**Secondary outcomes** PM&R perspective: The Timed Up and Go (TUG) test is an appropriate functional mobility measure, validated in cardiac rehabilitation populations (Bellet et al., 2013, reference 16). However, the following should be noted:

- The TUG was assessed only at 48 hours. While the authors state that sleep quality and functional mobility were evaluated only at 48 hours for logistical reasons, no justification is provided for this single-point approach. A baseline TUG value before intervention is clinically important because postoperative deconditioning, pre-existing mobility limitations (common in CABG patients aged 44–75), and ICU-related weakness could confound between-group differences at 48 hours if pre-intervention performance is not controlled for. The absence of a baseline TUG is a significant methodological weakness from a rehabilitation science perspective.
- Sleep quality, fatigue, and anxiety were all measured using 100-mm VAS instruments. While VAS for sleep and anxiety has been validated in postoperative populations (references 13–15), using the same scale type for four different constructs without psychometric differentiation (e.g., a validated sleep-specific instrument such as the Pittsburgh Sleep Quality Index, even in abbreviated form) limits the granularity of findings. For a 48-hour acute postoperative window, VAS-based sleep assessment may be defensible, but this choice should be more explicitly justified.
- SpO₂ is measured but shows no significant between-group differences at any time point (p values 0.62–0.88). While this is an honest null finding, the authors' interpretation that ETT "may lessen guarded breathing and promote more effective ventilation" (Discussion, line 323) is not supported by these data and represents speculative overclaiming.

**2c. Statistical Analysis**

The primary outcome (VAS) was analyzed using two-way mixed-effects ANOVA (group × time) with Bonferroni post-hoc correction, which is appropriate. Effect sizes are reported as partial η² and Cohen's d, consistent with current reporting standards.

The reported group × time interaction (F(6,384) = 4.12, p=0.001, partial η²=0.089) indicates a small-to-medium effect, which is consistent with the a priori power calculation (Cohen's d = 0.49).

Several statistical concerns warrant attention:

**Missing data handling:** Last observation carried forward (LOCF) was used for 7 participants (3.4%) lost to follow-up. While the authors state that complete-case sensitivity analyses yielded similar results, the actual sensitivity analysis results are not reported anywhere in the manuscript. PLOS ONE and current CONSORT standards require that these results be reported, not merely mentioned.

**Analgesic data:** Tramadol is reported as mean ± SD (50 ± 50 mg in the ETT group), and the authors clarify in Table 3 that this includes patients who received 0 mg (i.e., did not require rescue analgesia). A standard deviation equal to the mean in a rescue analgesia variable strongly suggests a highly skewed, zero-inflated distribution. Reporting the proportion of patients who required any tramadol in each group, and using appropriate non-parametric or zero-inflated modeling for this outcome, would be more statistically rigorous than ANOVA-based comparison of means.

**Correlation analysis:** The Spearman correlations reported in Table 4 (e.g., VAS 48h and TUG: rₛ=0.684; VAS 48h and sleep quality: rₛ=−0.712) are strong and clinically plausible. However, these are aggregate correlations across all 195 participants from three different groups who received fundamentally different interventions. Reporting these correlations without stratification by group — or at minimum acknowledging that the correlations conflate treatment effect with natural recovery — is a methodological limitation that should be explicitly discussed.

**3. DATA-BACKED CONCLUSIONS**

- The primary conclusions are largely supported by the data. The reduction in resting VAS (from ~65 to 18 in the ETT group vs. ~38–40 in controls at 48 hours) substantially exceeds the minimal clinically important difference (MCID) for postoperative VAS pain (~15–20 mm, per reference 20), and the statistical significance is robust (p=0.001 with Bonferroni correction). The conclusion that ETT produces clinically meaningful pain reduction is appropriate.
- The opioid-sparing conclusion (reduced tramadol use, p=0.038) is supported statistically, though the distributional issues noted above (zero-inflated, highly skewed data) mean the analytical method used may not be optimal. The direction and magnitude of the finding are plausible and clinically relevant, particularly for an older comorbid population.
- The LOS reduction (median 4 vs. 5 days, p=0.042) is a borderline significant finding with a confidence interval that is not reported. Given that this is a secondary outcome with a p-value near the significance threshold and no adjustment for multiple comparisons, the authors should frame this conclusion more cautiously. The statement that participants in the ETT group were "discharged approximately one day earlier" is accurate but should be accompanied by the full distributional data and an acknowledgment of the provisional nature of this finding.

The following specific conclusions are not well-supported and require revision:

- Overclaimed SpO₂ interpretation: As noted above, the claim that ETT may reduce guarded breathing and improve ventilation (Discussion, lines 323–328) is speculative given null SpO₂ findings across all time points. This should be reframed as a hypothesis for future investigation rather than a supported conclusion.
- Anxiety non-finding used to support mechanism: The authors argue that the absence of between-group anxiety differences confirms that ETT's analgesic effect is neurophysiological rather than expectancy-driven (lines 265–270). This is an overreach. Absence of evidence is not evidence of absence, particularly given that VAS-anxiety was assessed only once at 48 hours. Pre-intervention anxiety data were collected (Table 1) but the trajectory of anxiety over the intervention period is not reported.
- Cost-effectiveness language: The manuscript repeatedly references "cost-effectiveness" (lines 195, 337, 348) and includes what is described as "a simplified cost-effectiveness analysis." However, no actual cost data are presented anywhere in the manuscript. A reduction in LOS is a proxy for cost savings but is not a cost-effectiveness analysis. This language should be removed or replaced with a modest statement that the LOS finding suggests potential economic implications worthy of formal investigation.

**4. RESEARCH ETHICS**

- The ethical framework is appropriately documented. The study received approval from the Non-Interventional Clinical Research Ethics Committee of Bolu Abant Izzet Baysal University (Approval No: 2025/07; 21.01.2025) and was conducted in accordance with the Declaration of Helsinki. Written informed consent was obtained from all participants prior to surgery. Trial registration on ClinicalTrials.gov (NCT06910215, registered 2 April 2025) is reported.
- One concern warrants attention: the trial registration date (2 April 2025) postdates the stated commencement of participant enrollment (22 January 2025). This means the trial was not prospectively registered before enrollment began, which represents a deviation from ICMJE requirements and PLOS ONE policy for clinical trials. The authors should address this discrepancy explicitly. If enrollment began before registration, this should be disclosed as a limitation and the authors should confirm that no changes were made to outcomes or analyses after registration (which they do state at line 108–109, though this statement was made in reference to design changes generally). This is a potentially significant issue that the journal editorial office may need to adjudicate.
- The CE-certification of taping materials is noted, which is appropriate. No serious adverse events were reported, and skin monitoring procedures are described.

**6. REPORTING GUIDELINES AND DATA AVAILABILITY**

The manuscript includes a CONSORT checklist as a supplementary document. The trial report generally follows CONSORT 2010 and the nonpharmacological extension (Boutron et al., 2017, reference 8), with appropriate reporting of randomization, blinding, allocation concealment, and participant flow (Figure 1 is clear and complete).

Gaps in CONSORT adherence:

Blinding integrity assessment is absent, as noted (CONSORT nonpharmacological extension item 11b).

The sensitivity analysis results for missing data handling are mentioned but not reported (CONSORT item 12b).

The trial registration number is provided, but the relationship between the registered protocol and the published outcomes should be explicitly verified and stated, particularly given the post-enrollment registration date.

**Data Availability:** The data availability statement in the submission form reads: "The minimal data set [and accompanying code, where generated] is available at [repository name] via [DOI hyperlink]" This is a placeholder template that has not been completed with actual repository information. In the manuscript body (line 377), the authors state data are available from the corresponding author upon reasonable request. PLOS ONE's data policy requires that data be publicly deposited in an appropriate repository prior to publication, not merely available upon request. This must be rectified before publication can proceed.

**TOP 3 STRENGTHS**

1. Rigorous three-arm control design. The simultaneous inclusion of a tension-free placebo arm and an anatomically unrelated sham arm is a genuine methodological strength that directly addresses the most common criticism of physical intervention RCTs (inadequate blinding and control). This design provides a stronger causal inference platform than any prior ETT trial in the sternotomy literature.

2. Adequate statistical power with pre-specified sample size and effect size. The a priori power calculation, based on a published pooled effect size (King et al., reference 17), yielded 65 participants per group — a sample size that was met in the final analysis. The intention-to-treat approach and transparency about dropouts and their reasons further strengthen the internal validity of the study.

3. Clinically comprehensive outcome battery. The combination of subjective pain (VAS at rest and during coughing), objective functional mobility (TUG), patient-reported sleep quality and fatigue, objective analgesic consumption verified against nursing records, and LOS represents a genuinely multidimensional assessment of postoperative recovery that is relevant to both clinical practice and health systems planning. The strong correlations between these outcomes (Table 4) add convergent validity to the findings.

**TOP 3 WEAKNESSES / AREAS REQUIRING MAJOR REVISION**

1. Retrospective trial registration. The most serious methodological integrity concern is that trial registration occurred on 2 April 2025, while enrollment began on 22 January 2025, a gap of approximately 10 weeks. This violates ICMJE prospective registration requirements and PLOS ONE policy. The authors must provide a transparent explanation, confirm that primary outcomes were not changed after registration, and acknowledge this as a limitation. The journal will need to determine whether this is a disqualifying deviation.

2. Absence of baseline TUG measurement and blinding integrity assessment. The TUG was evaluated only at 48 hours, without a pre-intervention baseline. Given the age range and comorbidity burden of CABG patients, pre-existing differences in functional mobility could substantially confound the 48-hour TUG comparison. Similarly, the complete absence of a blinding integrity check in a physical intervention trial undermines the strength of the double-blinding claim, which is central to the manuscript's methodological novelty argument. Both omissions should be addressed, the latter at minimum through acknowledgment of this limitation and its implications.

3. Incomplete and inadequate data availability compliance. The data availability statement contains unfilled template text (no repository name or DOI), and the in-manuscript statement offers only corresponding-author access on request. This does not meet PLOS ONE's open data requirement. A complete, publicly accessible dataset must be deposited in an appropriate repository (e.g., OSF, Zenodo, Dryad) prior to acceptance, with the DOI provided in the final manuscript.

**CONSOLIDATED LIST OF SPECIFIC QUESTIONS AND COMMENTS FOR THE AUTHORS**

**Study Design and Registration**

The trial registration date (2 April 2025) appears to postdate the enrollment start date (22 January 2025). Please provide a full explanation of this discrepancy. Were primary and secondary outcomes identical in the registration record and the submitted manuscript? Please provide the registered outcomes for direct comparison.

Were block sizes fixed or variable in the randomization sequence? Please specify, as undisclosed fixed block sizes with an unblinded treating physiotherapist could theoretically compromise allocation concealment.

Was therapist contact time equalized across all three groups? Please clarify.

**Intervention Protocol**

Please specify the number of tape strips applied per patient, the length of each strip, and whether skin preparation was performed prior to taping.

How was tension standardized at 25–35%? What method was used to ensure consistent tension delivery across patients?

Was the tape applied once for the entire 48-hour period, or was reapplication performed? If reapplied, please specify when and under what criteria.

**Outcome Measures**

Why was TUG assessed only at 48 hours, without a baseline measure? Pre-intervention functional mobility could confound the 48-hour comparison, particularly in an older comorbid population. Please justify this design choice or, if baseline TUG data were collected, report them.

Was blinding integrity formally assessed (e.g., Bang's Blinding Index)? If so, please report the results. If not, please acknowledge this as a limitation with explicit discussion of how it affects interpretation.

The anxiety and sleep VAS data were collected at baseline (Table 1) but the trajectory of these variables across the 12, 24, and 48-hour time points is not reported. Were these measured at intermediate time points? If so, please report these data.

This review was conducted in accordance with PLOS ONE's peer review guidelines. The reviewer declares no conflict of interest with respect to the submitted work.
