# Supplementary material for: Effectiveness of elastic therapeutic taping in pain management after median sternotomy: A randomized controlled trial
Source: PLoS One. 2026 Sep 28;21(9):e0359282. doi: 10.1371/journal.pone.0359282 (PMC13618946; doi:10.1371/journal.pone.0359282)
Supplement: Supplementary file 6 [file pone.0359282.s007.docx]

01/07/2026

Dear Editor

I reviewed a manuscript entitled "Effectiveness of Elastic Therapeutic Taping in Pain Management After Median Sternotomy: A Randomized Controlled Trial" (PONE-D-26-16084R1) by Dr Alp Özel and colleagues for PLOS One.

Overall, the manuscript has improved substantially compared with the first submission, and the authors have addressed most of the previous concerns. However, I identified several remaining issues that should be corrected before acceptance.

The study is clinically relevant, the randomized three-arm design is a major strength, and the responses to reviewers are generally satisfactory. Nevertheless, several methodological inconsistencies, statistical issues, and reporting details remain.

I would be willing to recommend acceptance after the authors address the remaining reporting and statistical clarification points outlined below.

**Gul Baltaci, PT.Ph.D.Professor, CKTI.**

**Istanbul Atlas University**

**Faculty of Health Sciences**

**Dept of Physiotherapy and Rehabilitaiton**

**Minor revision**

This revision addresses the vast majority of concerns raised during peer review. The manuscript is methodologically stronger, better written, and considerably more transparent than the original submission. The remaining issues are relatively minor and relate primarily to reporting clarity rather than fundamental flaws in the study design or results.

**1. Modified intention-to-treat terminology still needs refinement:** The authors now state that a modified intention-to-treat (mITT) analysis was performed, which is more appropriate than the previous ITT claim. However, the manuscript states:

"including all randomized participants who received the allocated intervention and had evaluable post-intervention outcome data"

This is **not a standard mITT definition**, since patients without outcome data were excluded entirely.

The authors should:

- explicitly define mITT according to CONSORT recommendations;
- explain why no imputation was performed;
- clarify that the analysis is essentially a **complete-case modified ITT**.

**2. Randomization and allocation concealment remain incompletely described**

The revision improves the description of randomization, but **allocation concealment is still insufficiently reported**. The manuscript states:

Group assignments were implemented according to the pre-generated sequence.

This does not explain:

- Were opaque-sealed envelopes used?
- Was allocation concealed electronically?
- Who had access to the randomization list?
- Could recruiters predict upcoming allocations?

Without this information, selection bias cannot be fully assessed.

**3. Blinding remains potentially vulnerable**

The authors acknowledge that blinding success was not assessed.

This is appropriate. However, the manuscript should discuss that:

- active taping was applied around the sternotomy,
- sham taping was applied on the back,

meaning participants may have recognized group allocation.

This limitation deserves slightly stronger emphasis.

**4. Statistical analysis requires additional justification:** The authors justify mixed ANOVA by citing robustness. However, VAS values throughout the Results are reported as median (IQR) rather than means. This creates an inconsistency.

If ANOVA was used, authors should explain:

- whether residuals were normally distributed;
- whether assumptions were checked on model residuals rather than raw data;
- why means are not presented for the primary endpoint.

Alternatively, mixed-effects regression would be statistically stronger.

**5. Hospital stay remains vulnerable to confounding**

The Discussion has become more cautious, which is appreciated.

However, the Results still state:

participants in the ETT group were discharged approximately one day earlier

This wording still implies causality.

Suggested wording:

"Participants receiving ETT had a median hospital stay one day shorter than control groups; however, discharge timing may have been influenced by factors beyond the intervention."

**6. Table 2 inconsistency:** Length of hospital stay was not included in the multiple-comparison adjustment. Yet the response letter states this sentence was removed.

Please ensure the table matches the revised statistical approach.

**7. Sample size justification:** The sample size is based on Cohen's d = 0.49 from King et al. It would strengthen reproducibility if the manuscript briefly explained:

- which outcome from King et al. generated this estimate;
- whether it represents between-group or within-group effect size.

**8. Confidence intervals**

Confidence intervals have now been added for several outcomes, which is excellent. Consider also reporting:

- confidence intervals for LOS
- confidence intervals for sleep quality

to maintain consistency across secondary outcomes.

**9. Correlation analysis**

The correlation analysis appears exploratory. The manuscript should clearly state:

"These analyses were exploratory and hypothesis-generating." Otherwise readers may overinterpret them.

**10. Language:** English has improved considerably.

Only a few small issues remain.

Examples: "with scored recorded" should be "with scores recorded"

"Participant recruitment continued until the predetermined sample size of 195 participants who completed the study was achieved." This sentence could be smoother.

Suggested: "Recruitment continued until the target number of 195 evaluable participants was achieved."

**11. CONSORT flow** Ensure Figure 1 clearly distinguishes:

- randomized
- received intervention
- lost to follow-up
- analyzed (modified ITT)

using standard CONSORT terminology.

**12. Abstract:** The abstract is much improved.

However, "Functional mobility and sleep quality improved." should specify "compared with placebo and sham groups" to avoid ambiguity.
