# Supplementary material for: Effectiveness of elastic therapeutic taping in pain management after median sternotomy: A randomized controlled trial
Source: PLoS One. 2026 Sep 28;21(9):e0359282. doi: 10.1371/journal.pone.0359282 (PMC13618946; doi:10.1371/journal.pone.0359282)
Supplement: Supplementary file 7 [file pone.0359282.s008.docx]

We sincerely thank the Academic Editor and the reviewers for their careful evaluation of our manuscript and for their constructive comments and suggestions. We have carefully considered all comments and revised the manuscript accordingly. Detailed responses to each comment are provided below, and all corresponding changes have been incorporated into the revised manuscript.

Journal Requirements:

When submitting your revision, we need you to address these additional requirements.

1. Please ensure that your manuscript meets PLOS ONE's style requirements, including those for file naming. The PLOS ONE style templates can be found at

[https://journals.plos.org/plosone/s/file?id=wjVg/PLOSOne_formatting_sample_main_body.pdf](https://track.editorialmanager.com/CL0/https:%2F%2Fjournals.plos.org%2Fplosone%2Fs%2Ffile%3Fid=wjVg%2FPLOSOne_formatting_sample_main_body.pdf/1/010f019e8110ae19-24e1fe89-0dee-4e0f-b36d-7ccecb9e4cef-000000/MMFFBCfUJgUJy8UUhkByxpDVAnvU9x31Ey7z3lZ3DYE=258) and [https://journals.plos.org/plosone/s/file?id=ba62/PLOSOne_formatting_sample_title_authors_affiliations.pdf](https://track.editorialmanager.com/CL0/https:%2F%2Fjournals.plos.org%2Fplosone%2Fs%2Ffile%3Fid=ba62%2FPLOSOne_formatting_sample_title_authors_affiliations.pdf/1/010f019e8110ae19-24e1fe89-0dee-4e0f-b36d-7ccecb9e4cef-000000/5ccJS7OM5vCvXz2wfyMpdcx2p-68BCzsc_w3PY24LTk=258)

**Response:** Thank you for this comment. The manuscript has been carefully revised to comply with PLOS ONE formatting and style requirements. The title page, author affiliations, section headings, manuscript formatting, and file naming conventions have been reviewed and updated in accordance with the PLOS ONE style templates.

2. PLOS ONE requires that all clinical trials are registered in an appropriate registry (the WHO list of approved registries is at [https://www.who.int/clinical-trials-registry-platform/network/primary-registries](https://track.editorialmanager.com/CL0/https:%2F%2Fwww.who.int%2Fclinical-trials-registry-platform%2Fnetwork%2Fprimary-registries/1/010f019e8110ae19-24e1fe89-0dee-4e0f-b36d-7ccecb9e4cef-000000/c4SoLZKgaJ4pXzHk10WimVmSWXCcE0RhCBreVEK62qg=258)" [https://www.who.int/clinical-trials-registry-platform/network/primary-registries](https://track.editorialmanager.com/CL0/https:%2F%2Fwww.who.int%2Fclinical-trials-registry-platform%2Fnetwork%2Fprimary-registries/2/010f019e8110ae19-24e1fe89-0dee-4e0f-b36d-7ccecb9e4cef-000000/sn2zhlAZ5nobOiTjBS4n7-KgOpuWuNhNrtfeb3xPFtM=258) and more information on trial registration is at [http://www.icmje.org/about-icmje/faqs/clinical-trials-registration/](http://track.editorialmanager.com/CL0/http:%2F%2Fwww.icmje.org%2Fabout-icmje%2Ffaqs%2Fclinical-trials-registration%2F/1/010f019e8110ae19-24e1fe89-0dee-4e0f-b36d-7ccecb9e4cef-000000/dqxtd9U1_9y5IOmTcP7WYifTFvtr2ReFBqgSgUY3YFc=258)).

**Response:** Thank you for highlighting this important issue. The trial was registered at ClinicalTrials.gov (NCT06910215). Ethical approval was obtained on January 21, 2025, and participant recruitment began on January 22, 2025. The trial record was first released on March 25, 2025 and publicly posted on April 2, 2025. We acknowledge that the registration was retrospective. The delayed registration resulted from administrative procedures during trial setup. Importantly, no changes were made to the study design, eligibility criteria, interventions, outcomes, sample size calculation, or statistical analysis plan after participant enrollment began or after trial registration. This information has now been explicitly added to the Methods section and acknowledged in the Limitations section. We also confirm that all ongoing and related trials for this intervention are registered

3. Please include captions for your Supporting Information files at the end of your manuscript, and update any in-text citations to match accordingly. Please see our Supporting Information guidelines for more information: [http://journals.plos.org/plosone/s/supporting-information](http://track.editorialmanager.com/CL0/http:%2F%2Fjournals.plos.org%2Fplosone%2Fs%2Fsupporting-information/1/010f019e8110ae19-24e1fe89-0dee-4e0f-b36d-7ccecb9e4cef-000000/pcArW9iMyIaOoPH5XGK7CSwZSF3NtxdysZUbPugPoGo=258).

**Response:** Thank you for this comment. Supporting Information captions have now been added at the end of the manuscript in accordance with the PLOS ONE Supporting Information guidelines. The supporting files were labeled sequentially (S1–S6) and their captions were included in the manuscript file.

4. We note that the grant information you provided in the ‘Funding Information’ and ‘Financial Disclosure’ sections do not match.

When you resubmit, please ensure that you provide the correct grant numbers for the awards you received for your study in the ‘Funding Information’ section

**Response:** Thank you for bringing this issue to our attention. We reviewed and standardized the funding information across the manuscript and submission system.

5. Please include your full ethics statement in the ‘Methods’ section of your manuscript file. In your statement, please include the full name of the IRB or ethics committee who approved or waived your study, as well as whether or not you obtained informed written or verbal consent. If consent was waived for your study, please include this information in your statement as well.

**Response:** Thank you for this comment. The ethics statement in the Methods section has been revised and expanded. We now provide the full name of the approving ethics committee (Bolu Abant Izzet Baysal University Non-Interventional Clinical Research Ethics Committee), the approval number and date, and clarification that written informed consent was obtained from all participants before enrollment.

6. If the reviewer comments include a recommendation to cite specific previously published works, please review and evaluate these publications to determine whether they are relevant and should be cited. There is no requirement to cite these works unless the editor has indicated otherwise.

**Response:** Thank you. No specific publications were recommended by the reviewers for citation.

**Review Comments to the Author**

We sincerely thank the reviewer for the valuable feedback. The comment has helped improve the clarity and transparency of the manuscript.

**Reviewer #1:** The trial registration date (2 April 2025) postdates the stated commencement of participant enrollment (22 January 2025). This means the trial was not prospectively registered before enrollment began, which represents a deviation from ICMJE requirements and PLOS ONE policy for clinical trials. The authors should address this discrepancy explicitly. If enrollment began before registration, this should be disclosed as a limitation and the authors should confirm that no changes were made to outcomes or analyses after registration (which they do state at line 108–109, though this statement was made in reference to design changes generally). This is a potentially significant issue that the journal editorial office may need to adjudicate.

**Response:** We thank the reviewer for identifying this important issue. We acknowledge that the study was registered retrospectively. Ethical approval was obtained before recruitment commenced (January 21, 2025), and participant enrollment began on January 22, 2025. The trial record was subsequently released on ClinicalTrials.gov on March 25, 2025 and publicly posted on April 2, 2025. The delay in registration was related to administrative procedures during trial setup. Importantly, no changes were made to the study design, eligibility criteria, interventions, outcomes, sample size calculation, or statistical analysis plan after participant enrollment began or after trial registration. This clarification has now been added to the Methods section and acknowledged as a limitation in the revised manuscript.

**Reviewer #2: GENERAL COMMENTS**

I appreciate the opportunity to review manuscript #PONE-D-26-16084 titled “Effectiveness of Elastic Therapeutic Taping in Pain Management After Median Sternotomy: A Randomized Controlled Trial". I enjoyed reading this.

I have several comments and suggestions detailed in my specific comments that follow, that I believe will strengthen this work. I also note that some improvement in the writing of this manuscript in the English language is needed.

**Response:** We sincerely thank the reviewer for the constructive comments and thoughtful suggestions, which have helped improve the quality and clarity of the manuscript.

SPECIFIC COMMENTS

*Page 3 (Abstract) – In your Results, suggest that you state whether any of the differences you observed were considered to be clinically important.

**Response:** Thank you for this valuable suggestion. We agree that reporting clinical relevance alongside statistical significance improves the interpretation of the findings. Therefore, the Abstract Results section has been revised to indicate that the observed reduction in pain was clinically meaningful and exceeded the minimal clinically important difference reported for postoperative pain.

*Page 3 (Keywords) – Suggest that you add “randomized controlled trial” as a keyword.

**Response:** Thank you for this suggestion. We have added “randomized controlled trial” to the list of keywords.

*Page 4, lines 93-95 (Introduction) – Suggest that you be more cautious with this statement and insert something like “To the best of the investigative team’s knowledge, no adequately powered, double blind…”

**Response:** Thank you for this suggestion. We agree that a more cautious wording is appropriate. Accordingly, the statement has been revised to read: “To the best of the investigators’ knowledge, no adequately powered, double-blind randomized trial has comprehensively evaluated the opioid-sparing and functional recovery effects of sternum-targeted elastic taping following CABG.”

*Page 6, lines 125-126 (Methods, Inclusion and Exclusion Criteria) – Suggest that you tell the reader whether males and/or females were eligible for inclusion in the study.

**Response:** Thank you for this comment. We have clarified the inclusion criteria by explicitly stating that both male and female patients were eligible for participation in the study.

*Page 7, line 164 (Methods, Functional Mobility) – For the TUG test, suggest that you insert the term “single-task” after the word “standardized”.

**Response:** Thank you for this suggestion. We have revised the Methods section to clarify that functional mobility was assessed using the standardized single-task Timed Up and Go (TUG) test.

*Page 8, lines 192-193 (Methods, Statistical Analysis) – For an outcome such as the single-task TUG test this would traditionally be tested using a statistical test for continuous data (ANOVA, etc.) versus the Kruskal-Wallis test. I’m assuming that you used the Kruskal-Wallis test because the data for all three of these outcomes (sleep quality, fatigue, TUG test) were not normally distributed. If so, please make it clear to the reader as to why you used the Kruskal-Wallis test.

**Response:** Thank you for this comment. We used the Kruskal-Wallis test because TUG, sleep quality, and length of hospital stay data did not meet the assumption of normality according to the Shapiro-Wilk test and were therefore summarized as median (interquartile range). To improve clarity, we have revised the Statistical Analysis section to explicitly state the rationale for using the Kruskal-Wallis test for these outcomes.

*Pages 8 and 9 (Methods, Statistical Analysis) – Beyond tests of statistical significance, was any consideration given to what differences might be clinically important? For example, a minimal clinically important difference of 10 to 15 mm (points) on a 0-100 Visual Analog Scale for pain.

**Response:** Thank you for this important comment. Clinical importance was not formally incorporated into the prespecified statistical analysis plan. However, we agree that interpretation of the findings should consider both statistical and clinical significance. Therefore, we have revised the Discussion section to relate the observed pain reduction to the published minimal clinically important difference (MCID) of 9.9 mm for acute postoperative pain measured on a 100-mm visual analog scale. The approximately 20–22 mm reduction observed in the ETT group exceeded this threshold, supporting the clinical relevance of the findings.

*Pages 10-15 (Discussion) - For ease of reading, suggest that you partition your Discussion into the following subsections and address: (1) Overall findings, (2) Implications for research, (3) Implications for practice, and (4) Strengths and potential limitations. You can obviously have additional subsections listed under these.

**Response:** Thank you for this helpful suggestion. To improve readability and facilitate interpretation, we reorganized the Discussion section by introducing clearer subsections addressing the overall findings, implications for practice, and strengths and limitations of the study. We believe this revised structure improves the flow and accessibility of the Discussion

*Page 10, line 238 (Discussion) – Suggest that you insert the term “3-arm” before the word “randomized”.

**Response:** Thank you for this suggestion. We have revised the sentence in the Discussion section to specify that this was a 3-arm randomized controlled study

*Page 10-11 (Discussion, Pain Control and Underlying Mechanisms) – Please tell the reader what minimal clinically important difference you’re referring to here.

**Response:** Thank you for this comment. We have clarified the statement by specifying the minimal clinically important difference (MCID) to which we referred. The Discussion section now states that the approximately 20–22 mm reduction in VAS scores observed in the ETT group exceeded the published MCID of 9.9 mm for acute postoperative pain measured on a 100-mm visual analog scale, thereby supporting the clinical relevance of the findings.

*Page 11, lines 257-259 (Discussion, Control and Underlying Mechanisms) – Since this was a 3-arm study, what two groups are you referring to here?

**Response:** Thank you for this comment. We agree that the original wording was ambiguous in the context of a 3-arm study. We have revised the sentence to explicitly state that no significant differences were observed between the placebo and sham groups, thereby clarifying the comparison being referenced.

*Page 15, lines 352-360 (Limitations and Confounders) – First, at the beginning of this section, suggest that you tell the reader what the major strength(s) of this study was/were. Second, another limitation that should be mentioned is that the results of this study may not be generalizable to other populations.

**Response:** Thank you for this helpful suggestion. We have revised the section to begin with the major strengths of the study, including its three-arm randomized design, placebo and sham control groups, and a priori sample size calculation. We have also added a statement acknowledging that the single-center design may limit the generalizability of the findings to other healthcare settings and patient populations.

END OF REVIEW

**Reviewer #3:** Thank you for the opportunity to review this manuscript entitled *“Effectiveness of Elastic Therapeutic Taping in Pain Management After Median Sternotomy: A Randomized Controlled Trial.”* The topic is clinically relevant, particularly given the need for non-pharmacological, opioid-sparing strategies after cardiac surgery. The randomized design, inclusion of both placebo and sham taping groups, and assessment of pain during coughing as well as at rest are notable strengths. The manuscript suggests potentially meaningful benefits of elastic therapeutic taping on postoperative pain, functional mobility, analgesic consumption, sleep quality, and length of hospital stay. However, several important methodological, statistical, reporting, and interpretation issues need to be addressed before the manuscript can be considered further.

**Response:** We appreciate the reviewer’s detailed and constructive evaluation. All comments have been carefully considered and addressed in the revised manuscript.

I. Major Comments

1. Trial registration timing requires clarification

The manuscript states that recruitment began on January 22, 2025, while the trial was registered on ClinicalTrials.gov on April 2, 2025. This suggests that the trial may have been registered after participant enrollment had already started. The authors should explicitly clarify whether registration was prospective or retrospective. If any participants were enrolled before registration, this must be clearly stated in the Methods and discussed as a limitation.

**Response:** We appreciate the reviewer’s comment. We acknowledge that the trial was registered retrospectively. Ethical approval was obtained on January 21, 2025, participant recruitment started on January 22, 2025, the trial record was first released on March 25, 2025, and publicly posted on April 2, 2025. The delayed registration resulted from administrative procedures during trial setup. No modifications were made to the study design, eligibility criteria, interventions, outcome measures, sample size calculation, or statistical analysis plan after recruitment had started or after registration. To improve transparency, this information has been explicitly incorporated into the Methods section and discussed in the Limitations section of the revised manuscript.

2. The claim of intention-to-treat analysis is not fully supported

The manuscript reports that 203 participants were randomized, but only 195 were included in the final analysis. At the same time, the authors state that analyses were conducted according to the intention-to-treat principle and that no participants were excluded from the analysis. This is internally inconsistent. A true intention-to-treat analysis should include all randomized participants. The authors should clarify exactly which population was analyzed: intention-to-treat, modified intention-to-treat, per-protocol, or complete-case. The handling of the 7–8 participants lost to follow-up should be described more transparently.

**Response:** Thank you for this important comment. We agree that the previous wording was inconsistent. Although 203 participants were randomized, 195 participants who received the allocated intervention and had evaluable post-intervention outcome data were included in the primary analysis. Therefore, the analysis population is more accurately described as a modified intention-to-treat population rather than a full intention-to-treat population. We have revised the manuscript accordingly and removed the statement that “no participants were excluded from the analysis.” We also clarified the number and reasons for loss to follow-up in each group and described the handling of missing data more transparently.

3. Missing data handling needs more detail

The manuscript states that missing data were handled using last observation carried forward (LOCF), with similar results in complete-case sensitivity analyses. However, the extent, pattern, and timing of missing data are not sufficiently described. The authors should provide the number of missing observations per outcome and group, explain why LOCF was chosen, and present sensitivity analysis results either in the main text or supplementary material.

**Response:** Thank you for this comment. We agree that the description of missing data handling required clarification. Seven participants were lost to follow-up after randomization (ETT, n=2; PT, n=3; ST, n=2) and therefore did not contribute post-intervention outcome data. The final analysis was conducted on the 195 participants with available outcome data. We have revised the manuscript to clearly describe the number of participants lost to follow-up in each group and removed statements that could imply formal imputation procedures.

4. Randomization and allocation concealment are insufficiently described

The manuscript mentions a computer-generated block randomization sequence, but does not adequately describe allocation concealment. The authors should state who generated the random sequence, who enrolled participants, who assigned interventions, whether sealed opaque envelopes or a centralized system were used, and whether block sizes were fixed or varied. This is essential for assessing selection bias.

**Response:** Thank you for this valuable comment. We have revised the Methods section to provide additional details regarding the randomization procedure. Specifically, we clarified that a computer-generated block randomization sequence was created using the Blockrand package in R prior to participant recruitment, using fixed blocks of six participants. We also specified that eligible participants were enrolled by the cardiovascular surgery team, that intervention assignments followed the pre-generated allocation sequence, and that participants and outcome assessors remained blinded throughout the study.

5. Blinding should be reported more rigorously

The study is described as double-blind, with participants and outcome assessors blinded. However, the active taping, placebo taping, and sham taping were applied to different anatomical regions or with different tension, which may have been distinguishable to participants. The authors should describe whether blinding success was assessed, for example by asking participants or assessors to guess group allocation. If blinding was not formally assessed, this should be acknowledged as a limitation.

**Response:** Thank you for this comment. Blinding success was not formally assessed in this study. This limitation has now been acknowledged in the Discussion section.

6.The analgesic protocol appears inconsistent with the results

The Methods state that all participants received scheduled intravenous paracetamol 1000 mg, while Table 3 shows significantly lower total paracetamol use in the ETT group compared with control groups. If paracetamol was scheduled equally for all participants, it is unclear why total paracetamol differed substantially between groups. The authors should clarify whether paracetamol was truly scheduled, rescue-based, discontinued early, or adjusted according to clinical status. This point is important because analgesic consumption is a key secondary outcome.

**Response:** Thank you for this comment. Intravenous paracetamol was included in the standard postoperative analgesic protocol; however, total analgesic consumption was not fixed and depended on individual analgesic requirements during the postoperative period. We have clarified this point in the Methods section.

7. VAS threshold for rescue tramadol should be corrected or clarified

The Methods state that rescue tramadol was administered when VAS ≥4. Since pain was measured using a 100-mm VAS, the threshold should presumably be ≥40 mm, unless the authors converted the scale to 0–10 for clinical decision-making. This should be clarified and standardized throughout the manuscript.

**Response:** Thank you for this comment. We agree that the wording was unclear. Pain intensity was assessed using a 100-mm visual analog scale (VAS), and rescue tramadol was administered when the VAS score was ≥40 mm. The Methods section has been revised accordingly for consistency throughout the manuscript.

8. Statistical methods require reconsideration and clearer justification

The authors present many outcomes as medians and interquartile ranges, suggesting non-normal distributions, but the primary repeated pain outcome was analyzed using two-way mixed-effects ANOVA. The authors should justify the use of parametric repeated-measures analysis for VAS data or consider a more appropriate model, such as a mixed-effects model with robust methods or ordinal/nonparametric repeated-measures approaches. The manuscript should also clearly distinguish between mixed-effects ANOVA and mixed-effects modeling, as these terms are not interchangeable.

**Response:** Thank you for this comment. The primary outcome (VAS pain scores) was analyzed using a two-way mixed ANOVA because the study was designed to evaluate group, time, and group-by-time interaction effects across repeated measurements. Although some secondary outcomes did not meet normality assumptions and were therefore analyzed using non-parametric methods, the VAS analysis was performed using a parametric repeated-measures approach. Given the balanced design, equal group sizes, and sample size of 65 participants per group, mixed ANOVA was considered sufficiently robust to moderate departures from normality. To avoid ambiguity, the term “two-way mixed ANOVA” has been used consistently throughout the manuscript.

9. Multiple comparison correction is inconsistently applied

The authors state that length of hospital stay was excluded from multiple-comparison correction because it was a prespecified secondary outcome. Prespecification alone does not necessarily justify exclusion from multiplicity adjustment when multiple secondary outcomes are tested. The authors should provide a clearer statistical rationale and interpret secondary outcomes more cautiously.

**Response:** Thank you for this comment. We agree that prespecification alone does not eliminate concerns regarding multiplicity. Therefore, we have removed this statement and interpreted secondary outcomes, including length of hospital stay, as supportive findings that should be interpreted with appropriate caution.

10.Length of hospital stay may be confounded

The reported one-day reduction in hospital stay is clinically interesting, but discharge after CABG is influenced by many factors beyond pain and mobility, including surgical complications, arrhythmias, wound status, hemodynamic stability, social factors, and institutional discharge policies. The authors should report standardized discharge criteria and consider adjusted analyses for relevant clinical covariates. Without this, the conclusion that ETT shortened hospitalization should be stated more cautiously.

**Response:** Thank you for this comment. We agree that length of hospital stay may be influenced by multiple clinical and non-clinical factors beyond pain and functional recovery. Standard postoperative discharge decisions were made by the treating cardiovascular surgery team according to routine institutional practice. As detailed data on all potential determinants of discharge were not collected, adjusted analyses were not performed. We have revised the Discussion section to interpret the observed reduction in length of stay more cautiously.

11. Cost-effectiveness claims are underdeveloped

The manuscript mentions a “simplified cost-effectiveness analysis,” but no methods, assumptions, cost inputs, or results are presented. Either a transparent economic analysis should be added, or the cost-effectiveness claims should be removed or substantially softened.

**Response:** Thank you for this comment. We agree that the manuscript did not include a formal economic evaluation with predefined cost inputs, assumptions, or cost-effectiveness outcomes. Therefore, we have removed references to a cost-effectiveness analysis and revised the manuscript to limit discussion to the observed reductions in analgesic consumption and length of hospital stay.

12. Clinical significance should be reported more completely

The authors state that pain reduction exceeded the minimal clinically important difference. This is useful, but the manuscript should report between-group differences with confidence intervals at each time point, especially for the primary outcome. Reporting only p-values limits the interpretation of clinical importance.

**Response:** Thank you for this comment. We agree that confidence intervals provide important information regarding the magnitude and precision of treatment effects. We have now reported between-group differences with 95% confidence intervals for the primary pain outcome at each postoperative assessment time point.

13. Adverse events and safety reporting need more detail

The manuscript states that no serious adverse events occurred and that skin reactions were monitored. The authors should provide the number and type of any minor adverse events by group, including redness, itching, irritation, tape intolerance, or early tape removal. If no adverse events occurred at all, this should be explicitly stated.

**Response:** Thank you for this comment. We have revised the manuscript to provide more detailed safety information. No serious adverse events occurred in any group. Minor adverse events were limited to two cases of mild skin redness in the ETT group and one case of mild itching in the placebo group. No adverse events were reported in the sham group. All reactions were transient, did not require medical treatment, and did not result in premature tape removal or study withdrawal.

14. Data availability statement is not compliant with PLOS ONE expectations

The submission form contains a placeholder-style statement indicating that the minimal dataset is available at a repository via DOI, while the manuscript states that data are available from the corresponding author upon reasonable request. This inconsistency must be resolved. PLOS ONE generally expects the minimal dataset underlying the findings to be publicly available unless ethical or legal restrictions apply. The authors should provide a repository link/DOI or a clear justification for restricted access.

**Response:** Thank you for this comment. We have revised the Data Availability Statement to ensure consistency throughout the manuscript and submission materials. The anonymized minimal dataset underlying the findings of this study is now publicly available in the Zenodo repository (https://doi.org/10.5281/zenodo.20922725).

15. Figures require revision

The submitted figures appear cropped or difficult to read, particularly the CONSORT flow diagram and graphs. Figures should be uploaded in higher resolution, with complete legends, readable axis labels, clear group labels, and no cropped content. Figure 3 should clearly distinguish paracetamol and tramadol or explain whether it represents total analgesic dose, as combining medications with different dosing ranges may be misleading.

**Response:** Thank you for your valuable comment. The CONSORT flow diagram and all figures have been regenerated in high resolution (600 dpi) with improved readability, including larger axis labels, clearer group labels, and complete figure legends. In addition, Figure 3 has been revised to present paracetamol and tramadol consumption separately in two panels to avoid potential misinterpretation due to their different dosing ranges.

16. The discussion overstates causality in places

The manuscript attributes improvements to mechanoreceptive stimulation, biomechanical support, trunk stabilization, and proprioceptive mechanisms. While plausible, these mechanisms were not directly measured. The discussion should more clearly distinguish demonstrated clinical outcomes from speculative mechanisms.

**Response:** Thank you for this valuable comment. The Discussion has been revised to more clearly distinguish the observed clinical outcomes from the proposed mechanisms of action. We have softened statements implying causality and explicitly clarified that the neurophysiological and biomechanical mechanisms discussed are biologically plausible but were not directly evaluated in the present study.

17. The manuscript needs careful language editing

There are several grammatical and typographical errors, including “couging,” “withcompletion,” “haspital,” “PT Group),” inconsistent spacing, and awkward phrasing in the Discussion. A full professional language edit is recommended before resubmission.

**Response:** Thank you for your careful review. The manuscript has been thoroughly edited to correct grammatical, typographical, and formatting errors, including those highlighted by the reviewer. We also carefully revised the wording throughout the manuscript to improve clarity, consistency, and overall readability.

II. Minor Comments

1. Please standardize terminology throughout the manuscript: “elastic therapeutic taping,” “Kinesio taping,” “elastic taping,” and “ETT” are used somewhat interchangeably.

**Response:** Thank you for this helpful suggestion. We have standardized the terminology throughout the manuscript. The intervention is now consistently referred to as elastic therapeutic taping (ETT) after its first definition, and inconsistent terms such as "Kinesio taping" and "elastic taping" have been replaced where appropriate.

2. Please ensure consistency in reporting country names: “Turkiye,” “Türkiye,” and “Turkey” should be standardized according to journal style.

**Response:** Thank you for this comment. All occurrences have been revised to "Turkey" for consistency.

3. The Abstract should be more cautious. For example, “shortens hospitalization” may be revised to “was associated with a shorter length of hospital stay.”

**Response:** Thank you for this helpful suggestion. We have revised the Abstract to use more cautious language. Statements implying causality have been replaced with associative wording, including revising "shortens hospitalization" to "was associated with a shorter length of hospital stay.

4. The Methods should specify whether outcomes were assessed by the same blinded assessor throughout or by multiple assessors, and whether inter-rater reliability was considered.

**Response:** Thank you for this important comment. We have clarified the Methods to specify that all outcome assessments were performed by the same physiotherapist throughout the study, while the intervention was administered by a different physiotherapist. Because all measurements were obtained by the same assessor, inter-rater reliability was not applicable.

5. The sample size calculation should explain why Cohen’s d=0.49 was appropriate for a three-arm repeated-measures trial and whether attrition was accounted for.

**Response:** Thank you for this comment. We have revised the sample size calculation to clarify the rationale for the selected effect size and to specify that participant recruitment continued until the target sample size was achieved despite losses during follow-up.

6. Baseline sleep quality, fatigue, and anxiety are reported, but the Methods state that sleep quality and functional mobility were evaluated only at 48 hours. Please clarify which outcomes were measured at baseline and which only after intervention.

**Response:** Thank you for this comment. We have clarified the Methods to specify the assessment time points for each outcome, including baseline and post-intervention measurements, to ensure consistency with the reported results.

7. Please report whether any patients required ICU readmission, reoperation, or had postoperative complications, even if not primary outcomes.

**Response:** Thank you for this valuable comment. We have added a statement to the Results section indicating that no patients required ICU readmission or reoperation, and no major postoperative complications were observed during the study period.

8. Please clarify whether NSAID use was standardized or clinician-dependent.

**Response:** Thank you for this comment. We have clarified in the Methods that NSAID administration was not standardized and was left to the discretion of the treating physician according to routine clinical practice

9. Table formatting should be improved. Some values use brackets while others use parentheses, and superscript notation is not always clear.

**Response:** Thank you for this comment. We have revised the tables to ensure consistent formatting, notation, and presentation throughout the manuscript.

10. Please provide confidence intervals for key effect estimates, including pain scores, TUG, tramadol use, and length of stay.

**Response:** Thank you for this valuable suggestion. We have revised the Results section to include 95% confidence intervals for the primary pain outcomes and key secondary effect estimates, where appropriate.
