# Supplementary material for: Effectiveness of elastic therapeutic taping in pain management after median sternotomy: A randomized controlled trial
Source: PLoS One. 2026 Sep 28;21(9):e0359282. doi: 10.1371/journal.pone.0359282 (PMC13618946; doi:10.1371/journal.pone.0359282)
Supplement: Supplementary file 8 [file pone.0359282.s009.docx]

We sincerely thank the Academic Editor and the reviewers for their careful evaluation of our manuscript and for their constructive comments and valuable suggestions. We have carefully considered all comments and revised the manuscript accordingly. Detailed responses to each comment are provided below, and all corresponding changes have been incorporated into the revised manuscript.

Additional Editor Comments:

Dear Authors,
Your revised manuscript on elastic therapeutic taping after median sternotomy has undergone peer review. The evaluation identified several methodological and reporting issues that require attention before the work can be reconsidered. First, the analysis of the primary pain outcome is not described consistently throughout the text. You should state plainly which method provides the primary between-group comparison and how that choice fits the way the data are presented. Second, the manuscript departs from the missing-data handling and analysis plan set out in the registered protocol, and these departures should be reconciled and justified. In addition, concealment of the randomization sequence and the way blinding was maintained are not sufficiently described in the main text. As the manuscript now stands, the risk of selection and assessment bias cannot be judged. Several interpretations in the discussion, in particular the causal reading of the hospital-stay difference, go beyond what the design supports and require more cautious wording that acknowledges confounding. The repeated and conflicting passages in the text should also be resolved. The decision is major revision. These issues arise not from the design itself but from the consistency of the analysis and reporting. Once they have been addressed, a revised version may be submitted.
Sincerely,

**Response:** We thank the Academic Editor for the thoughtful and constructive comments. We have carefully revised the manuscript to address all of the issues raised. Specifically, we clarified the statistical approach for the primary outcome, reconciled and justified the differences between the registered protocol and the final analysis, expanded the description of randomization and blinding procedures, revised the discussion to avoid causal interpretation of the hospital length of stay findings, and corrected repeated or inconsistent statements throughout the manuscript. Detailed responses to each comment are provided below.

Review Comments to the Author

**Reviewer #4:** Thank you very much for your valuable work. Please address all the comments in detail.

Abstract
1. Please provide a detailed statistical analysis.

**Response:** Thank you for this valuable comment. We have revised the statistical analysis in the Abstract to provide greater methodological detail and ensure consistency with the Methods section.

2. In lines 42-44, the observed between-group reduction in pain exceeded the reported minimal clinically important difference for acute postoperative pain, indicating a clinically meaningful treatment effect. Please specify the MCID value.

**Response:** Thank you for this valuable comment. The MCID value (9.9 mm) has now been specified in the Abstract.

3. Add the statistical value for the TUG and sleep quality.

**Response:** Thank you for this valuable comment. The statistical significance for the TUG test and sleep quality outcomes has been added to the Abstract.

4. Include the clinical trial registry number at the end of the abstract.

**Response:** Thank you for this valuable comment. The clinical trial registry number (ClinicalTrials.gov: NCT06910215) has been added at the end of the Abstract.

Introduction
5. Define ETT and elaborate on its physiological and biomechanical function.

**Response:** Thank you for this valuable comment. The Introduction has been revised to define ETT more clearly and to briefly describe its proposed physiological and biomechanical mechanisms of action.

6. Explain additional therapeutic management for coronary artery bypass grafting (CABG) patients following immediate postoperative care and prior to discharge.

**Response:** Thank you for this valuable comment. We have expanded the Methods section to describe the standard postoperative management provided to all participants from the immediate postoperative period until hospital discharge.

7. In line 74, add the word 'objective' or 'aim' of the study.

**Response:** Thank you for this valuable comment. We have revised the final paragraph of the Introduction to explicitly state the objective of the study.

Materials and methods
8. Specify the study setting and period in accordance with the study design.

**Response:** Thank you for this valuable comment. The study setting and study period have been explicitly added to the Study design section of the Methods.

9. Provide details on the randomization sequence and blinding in a separate heading.

**Response:** Thank you for this valuable comment. We have created a separate subsection entitled “Randomization and blinding” and moved the relevant methodological details to this section for improved clarity.

10. Include a link to the clinical trial registration details after the registry number.

**Response:** Thank you for this valuable comment. A direct link to the ClinicalTrials.gov registration record has been added after the trial registration number.

11. In line 115, expand on the baseline assessment.

**Response:** Thank you for this valuable comment. The description of the baseline assessment has been expanded to clarify the timing and the outcome measures assessed before the intervention.

12. Clarify the inclusion criteria.

**Response:** Thank you for this valuable comment. The inclusion criteria have been clarified to provide a more complete description of participant eligibility and to ensure consistency with the registered study protocol.

13. Explain the duration of taping applications for all three groups.

**Response:** Thank you for this valuable comment. The duration of the taping application has been clarified for all three groups in the Methods section.

14. Add images of all taping procedures in accordance with ethical guidelines.

**Response:** Thank you for this valuable suggestion. Representative photographs of the elastic therapeutic taping, placebo taping, and sham taping procedures have been added as Figure 2. In addition, the Ethics section has been revised to clarify that written informed consent for publication of the clinical photographs was obtained from all participants whose images are included in the manuscript.

15. In line 185, elaborate on the length of stay (LOS).

**Response:** Thank you for this valuable comment. The description of length of hospital stay (LOS) has been expanded to clarify its definition and the routine clinical discharge process.

16. Include validity and reliability information for all outcome parameters and interventions, citing recent references.

**Response:** Thank you for this valuable comment. We have expanded the Methods section to provide validity and reliability information for the patient-reported and functional outcome measures. We also clarified the measurement procedures for SpO₂, analgesic consumption, LOS, and the standardization of the taping interventions, with additional references where appropriate.

Results and Discussion
17. Separate the results and discussion into distinct headings.

**Response:** Thank you for this valuable suggestion. We appreciate this recommendation. However, the manuscript has been prepared in accordance with the PLOS ONE manuscript formatting guidelines, which present the Results and discussion section as a combined heading. Therefore, we have retained the current structure to ensure consistency with the journal's required format.

18. Table 1 separates the numbers of male and female participants but omits surgical data when information is missing.

**Response:** Thank you for this comment. The variables presented in Table 1 correspond to the predefined baseline characteristics collected for this randomized controlled trial. Detailed intraoperative surgical variables were not predefined study variables and therefore were not collected.

19. Please add the p-value in Table 1.

**Response:** Thank you for this valuable suggestion. P values have now been added to Table 1 for all baseline comparisons.

20. In line 362, the statement that effective early pain control may limit central sensitization and reduce the risk of chronic pain is supported. Please add a reference for the above sentence.

**Response:** Thank you for this valuable suggestion. We have added appropriate references to support the statement regarding the potential role of effective early pain control in limiting central sensitization and reducing the risk of chronic postoperative pain.

21. In lines 369-371, the marked reduction in total analgesic and tramadol consumption in the ETT group confirms that the intervention not only alters subjective pain perception but also reduces objective medication requirements [6]. How do the authors ascertain that pain reduction is solely due to the elastic taping method?

**Response:** Thank you for this important comment. We agree that the original wording was too definitive. Accordingly, we revised the Discussion by replacing *"confirms"* with *"suggests"* and modified the text to avoid implying that the observed reduction in analgesic consumption was solely attributable to ETT.

22. In lines 417-418, the claim that ETT may indirectly improve sleep by reducing pain and stabilizing the chest wall needs a supporting reference.

**Response:** Thank you for this valuable suggestion. We have revised the statement and added two appropriate references to support the relationship between postoperative pain reduction and improved sleep quality.

**Reviewer #5:** This revision addresses the vast majority of concerns raised during peer review. The manuscript is methodologically stronger, better written, and considerably more transparent than the original submission. The remaining issues are relatively minor and relate primarily to reporting clarity rather than fundamental flaws in the study design or results.

1. Modified intention-to-treat terminology still needs refinement: The authors now state that a modified intention-to-treat (mITT) analysis was performed, which is more appropriate than the previous ITT claim. However, the manuscript states:
"including all randomized participants who received the allocated intervention and had evaluable post-intervention outcome data"
This is not a standard mITT definition, since patients without outcome data were excluded entirely.
The authors should:
• explicitly define mITT according to CONSORT recommendations;
• explain why no imputation was performed;
• clarify that the analysis is essentially a complete-case modified ITT.

**Response:** Thank you for this important comment. We have revised the Statistical Analysis section to clarify the definition of the modified intention-to-treat approach, stated that no imputation of missing outcome data was performed, and specified that the primary analysis represents a complete-case modified intention-to-treat analysis.

2. Randomization and allocation concealment remain incompletely described
The revision improves the description of randomization, but allocation concealment is still insufficiently reported. The manuscript states:
Group assignments were implemented according to the pre-generated sequence.
This does not explain:
• Were opaque-sealed envelopes used?
• Was allocation concealed electronically?
• Who had access to the randomization list?
• Could recruiters predict upcoming allocations?
Without this information, selection bias cannot be fully assessed.

**Response:** Thank you for this important comment. We have revised the Randomization and blinding section to provide additional details on the allocation procedure. We clarified that the computer-generated randomization sequence was generated before participant recruitment by the corresponding author. The sequence was not accessible to the cardiovascular surgery team responsible for participant enrollment. Group allocation was assigned only after participant enrollment according to the pre-generated randomization sequence. The physiotherapist responsible for tape application was informed of the assigned group immediately before tape application

3. Blinding remains potentially vulnerable
The authors acknowledge that blinding success was not assessed.
This is appropriate. However, the manuscript should discuss that:
• active taping was applied around the sternotomy,
• sham taping was applied on the back,
meaning participants may have recognized group allocation.
This limitation deserves slightly stronger emphasis.

**Response:** Thank you for this valuable comment. We have revised the Limitations section accordingly. We now acknowledge that blinding success was not formally assessed. We also note that the different taping locations may have allowed some participants to infer their group allocation.

4. Statistical analysis requires additional justification: The authors justify mixed ANOVA by citing robustness. However, VAS values throughout the Results are reported as median (IQR) rather than means. This creates an inconsistency.
If ANOVA was used, authors should explain:
• whether residuals were normally distributed;
• whether assumptions were checked on model residuals rather than raw data;
• why means are not presented for the primary endpoint.
Alternatively, mixed-effects regression would be statistically stronger.

**Response:** Thank you for this valuable comment. We agree that assessment of model residuals could provide additional information regarding model assumptions. To improve consistency between the statistical analysis and data presentation, the primary outcome is now presented as mean ± standard deviation, consistent with the use of the two-way mixed ANOVA. We also clarified the rationale for using the mixed ANOVA in the Statistical Analysis section.

5. Hospital stay remains vulnerable to confounding
The Discussion has become more cautious, which is appreciated.
However, the Results still state:
participants in the ETT group were discharged approximately one day earlier
This wording still implies causality.
Suggested wording:
"Participants receiving ETT had a median hospital stay one day shorter than control groups; however, discharge timing may have been influenced by factors beyond the intervention."

**Response:** Thank you for this valuable comment. We have revised the wording in the Results section to avoid implying a causal relationship.

6. Table 2 inconsistency: Length of hospital stay was not included in the multiple-comparison adjustment. Yet the response letter states this sentence was removed.
Please ensure the table matches the revised statistical approach.

**Response:** Thank you for identifying this inconsistency. The statement has been removed from Table 2.

7. Sample size justification: The sample size is based on Cohen's d = 0.49 from King et al. It would strengthen reproducibility if the manuscript briefly explained:
• which outcome from King et al. generated this estimate;
• whether it represents between-group or within-group effect size.

**Response:** Thank you for this helpful comment. We have clarified that the expected effect size (0.49) was based on the between-group standardized mean difference for postoperative pain reported by King et al.

8. Confidence intervals
Confidence intervals have now been added for several outcomes, which is excellent. Consider also reporting:
• confidence intervals for LOS
• confidence intervals for sleep quality
to maintain consistency across secondary outcomes.

**Response:** Thank you for this valuable suggestion. Confidence intervals were reported for the primary outcomes. As sleep quality and length of hospital stay were analyzed using non-parametric methods and are presented as median (IQR), confidence intervals were not added to maintain consistency with the statistical approach used

9. Correlation analysis
The correlation analysis appears exploratory. The manuscript should clearly state:
"These analyses were exploratory and hypothesis-generating." Otherwise readers may overinterpret them.

**Response:** Thank you for this valuable comment. We have clarified in both the Results and Discussion sections that the correlation analyses were exploratory and hypothesis-generating

10. Language: English has improved considerably.
Only a few small issues remain.
Examples: "with scored recorded" should be "with scores recorded"

"Participant recruitment continued until the predetermined sample size of 195 participants who completed the study was achieved." This sentence could be smoother.
Suggested: "Recruitment continued until the target number of 195 evaluable participants was achieved."

**Response:** Thank you for your helpful suggestions. The identified language issues have been corrected, and the manuscript has been carefully proofread.

11. CONSORT flow Ensure Figure 1 clearly distinguishes:
• randomized
• received intervention
• lost to follow-up
• analyzed (modified ITT)
using standard CONSORT terminology.

**Response:** Thank you for this helpful suggestion. Figure 1 has been revised using standard CONSORT terminology.

12. Abstract: The abstract is much improved.
However, "Functional mobility and sleep quality improved." should specify "compared with placebo and sham groups" to avoid ambiguity.

**Response:** Thank you for this helpful suggestion. The Abstract has been revised to clarify that improvements in functional mobility and sleep quality were observed compared with the placebo and sham groups.

**Reviewer #6:** Based on the reviewed revision manuscript, the article is fully suitable for publication in terms of scientific depth, statistical accuracy, methodological strength, transparency, and compliance with the open data policy. The authors have responded to all major and minor criticisms (mITT conversion, statistical justifications, removal of speculative statements, quality of visuals) from the editor and the three reviewers in a highly professional and satisfactory manner. The only potential risk facing the article, the fact that the study was recorded retrospectively, is explicitly acknowledged as a limitation in the revision text, and since the data is transparently disclosed in Zenodo, it will not pose an obstacle. It is anticipated that the journal editorial board will approve this revision and the article will be accepted and published. This is a highly successful revision manuscript with a very high chance of publication.

**Response:** We sincerely thank the reviewer for the positive evaluation of our manuscript and for recognizing the improvements made during the revision process. We greatly appreciate your encouraging comments.
