# Supplementary material for: Effectiveness of elastic therapeutic taping in pain management after median sternotomy: A randomized controlled trial
Source: PLoS One. 2026 Sep 28;21(9):e0359282. doi: 10.1371/journal.pone.0359282 (PMC13618946; doi:10.1371/journal.pone.0359282)
Supplement: Supplementary file 9 [file pone.0359282.s010.docx]

We sincerely thank the Academic Editor and the reviewers for their careful evaluation of our revised manuscript and for their constructive comments. We greatly appreciate their recognition of the improvements made during the previous revision rounds.

Response to the Academic Editor

On the methodological side, the single point to complete in the main text is the allocation concealment method. The text does not state whether sequentially numbered opaque sealed envelopes, centralized allocation, or another method was used. This information is needed so that the reader can assess the risk of selection bias, and it should be added in concrete terms.

The abstract still reports the primary outcome as median with interquartile range, whereas the main text and tables use mean with standard deviation. The resting and coughing pain values in the abstract should be brought into line with that format. A passage appears almost word for word twice in the discussion, and this repetition should be removed. Internally conflicting statements should also be corrected, for example the mismatch between the publication-consent statement and the presence of patient photographs. Finally, a language edit across the manuscript to remove a few conversational expressions would be appropriate

**Response:** We thank the Academic Editor for the careful assessment and constructive guidance. We have now described the allocation concealment method in concrete terms in the Randomization and blinding section. The pain values in the Abstract have been changed from median (IQR) to mean ± SD to ensure consistency with Table 2. We removed the duplicated passage from the Discussion and corrected the Consent for publication statement regarding the clinical photographs. We also revised the manuscript to replace conversational expressions with more formal scientific language.

Review Comments to the Author
Reviewer #5:

1. Statistical reporting is much more consistent: The manuscript now clearly states that:
• the primary endpoint was analyzed using a prespecified two-way mixed ANOVA
• secondary outcomes were analyzed using appropriate nonparametric methods
• Bonferroni correction was applied
• effect sizes are reported
• confidence intervals are reported for primary outcomes
• mITT is explicitly described as a complete-case modified intention-to-treat analysis

**Response:** Thank you for your positive assessment.

2. Randomization and blinding: The Randomization section is now much clearer. It specifies
• computer-generated block randomization
• sequence generated before recruitment
• recruitment team had no access
• treating physiotherapist separated from outcome assessment
• assessor blinded
These additions substantially reduce concerns about selection bias.

**Response:** Thank you for your positive assessment.

3. Discussion is much more balanced: Previously several statements implied causality. Now wording such as "suggests", "may", "potential explanation" is used appropriately. This is exactly what PLOS editors expect.

**Response:** Thank you for your positive assessment.

4. CONSORT compliance: The manuscript now includes
• trial registration
• flow diagram revisions
• clearer inclusion criteria
• intervention description
• photographs
• safety monitoring
• adverse events
• data availability
This considerably improves transparency.

**Response:** Thank you for your positive assessment.

5.Duplicate paragraph: There is one obvious duplication. The section: Clinical implications and safety appears twice almost word-for-word. Pages 23–24 contain repeated text. This duplication should be removed before acceptance.

**Response:** Thank you for noting this duplication. The repeated text has been removed.

6.One inconsistency still exists: In the Abstract pain values are still reported as median (IQR) for the primary outcome although the manuscript now states that the primary analysis uses mixed ANOVA and Table 2 reports mean ± SD. For consistency, the Abstract should also report: mean ± SD instead of median (IQR). This is probably the only remaining statistical inconsistency.

**Response:** Thank you for noting this inconsistency. The pain values in the Abstract have been revised to mean ± SD in accordance with Table 2.

7.Allocation concealment: The response letter explains allocation concealment well. However, the manuscript still never explicitly states whether allocation was performed using sequential opaque envelopes or centralized allocation or another concealment method. Instead it says group allocation was assigned according to the pre-generated sequence This is improved but still not ideal according to CONSORT. If the actual method was opaque envelopes, it should be explicitly stated. Otherwise reviewers may ask again.

**Response:** Thank you for highlighting this point. The corresponding author retained the sequence and disclosed the assignment to the treating physiotherapist only after participant enrollment had been completed. We have clarified the allocation concealment method in the Randomization and blinding section.

8.One sentence remains awkward: Page 20: "The significant decrease in pain that is caused by movement..."
A smoother wording would be "The significant reduction in movement-evoked pain suggests..." This is purely stylistic.

**Response:** Thank you for this suggestion. The sentence has been revised as recommended.

9.English polishing: The English is greatly improved. I still noticed occasional phrases such as wasn't optimally set up which sound conversational for a scientific manuscript. For example: "mechanical placement that wasn't optimally set up..." could become "suboptimal tape placement" which is more scientific.

**Response:** Thank you for this suggestion. The phrase has been revised using more formal scientific language.

10.Correlation section: You correctly state exploratory and hypothesis-generating which addresses the reviewer. Excellent improvement.

**Response:** Thank you for your positive assessment.

11.Discussion: The Discussion is now one of the strongest parts. It
• compares previous studies
• explains mechanisms cautiously
• discusses opioid reduction
• explains LOS carefully
• acknowledges limitations

**Response:** Thank you for your positive assessment.
