# Supplementary material for: Effectiveness of elastic therapeutic taping in pain management after median sternotomy: A randomized controlled trial
Source: PLoS One. 2026 Sep 28;21(9):e0359282. doi: 10.1371/journal.pone.0359282 (PMC13618946; doi:10.1371/journal.pone.0359282)
Supplement: Peer review history

# Peer review: Decision Letter 0

**DOI:** 10.1371/journal.pone.0359282.r001

**Authors:** Burak Katipoğlu

31 May 2026

Dear Dr. Özel,

Thank you for submitting your manuscript to PLOS ONE. After careful consideration, we feel that it has merit but does not fully meet PLOS ONE’s publication criteria as it currently stands. Therefore, we invite you to submit a revised version of the manuscript that addresses the points raised during the review process.

Please submit your revised manuscript by Jul 15 2026 11:59PM. If you will need more time than this to complete your revisions, please reply to this message or contact the journal office at plosone@plos.org. When you're ready to submit your revision, log on to https://www.editorialmanager.com/pone/ and select the 'Submissions Needing Revision' folder to locate your manuscript file.

- A letter that responds to each point raised by the academic editor and reviewer(s). You should upload this letter as a separate file labeled 'Response to Reviewers'.
- A marked-up copy of your manuscript that highlights changes made to the original version. You should upload this as a separate file labeled 'Revised Manuscript with Track Changes'.
- An unmarked version of your revised paper without tracked changes. You should upload this as a separate file labeled 'Manuscript'.

If you would like to make changes to your financial disclosure, please include your updated statement in your cover letter. Guidelines for resubmitting your figure files are available below the reviewer comments at the end of this letter.

If applicable, we recommend that you deposit your laboratory protocols in protocols.io to enhance the reproducibility of your results. Protocols.io assigns your protocol its own identifier (DOI) so that it can be cited independently in the future. For instructions see: https://journals.plos.org/plosone/s/submission-guidelines#loc-laboratory-protocols. Additionally, PLOS ONE offers an option for publishing peer-reviewed Lab Protocol articles, which describe protocols hosted on protocols.io. Read more information on sharing protocols at https://plos.org/protocols?utm_medium=editorial-email&utm_source=authorletters&utm_campaign=protocols.

As the corresponding author, your ORCID iD is verified in the submission system and will appear in the published article. PLOS supports the use of ORCID, and we encourage all coauthors to register for an ORCID iD and use it as well. Please encourage your coauthors to verify their ORCID iD within the submission system before final acceptance, as unverified ORCID iDs will not appear in the published article. *Only*  the individual author can complete the verification step; PLOS staff *cannot*  verify ORCID iDs on behalf of authors.

We look forward to receiving your revised manuscript.

Kind regards,

Burak Katipoğlu

Academic Editor

PLOS One

Journal Requirements:

When submitting your revision, we need you to address these additional requirements.

1. Please ensure that your manuscript meets PLOS ONE's style requirements, including those for file naming. The PLOS ONE style templates can be found at

https://journals.plos.org/plosone/s/file?id=wjVg/PLOSOne_formatting_sample_main_body.pdf and

https://journals.plos.org/plosone/s/file?id=ba62/PLOSOne_formatting_sample_title_authors_affiliations.pdf

2. PLOS ONE requires that all clinical trials are registered in an appropriate registry (the WHO list of approved registries is at https://www.who.int/clinical-trials-registry-platform/network/primary-registries" https://www.who.int/clinical-trials-registry-platform/network/primary-registries and more information on trial registration is at http://www.icmje.org/about-icmje/faqs/clinical-trials-registration/).

Please state the name of the registry and the registration number (e.g. ISRCTN or ClinicalTrials.gov) in the submission data and on the title page of your manuscript.

a) Please provide the complete date range for participant recruitment and follow-up in the methods section of your manuscript.

b) If you have not yet registered your trial in an appropriate registry, we now require you to do so and will need confirmation of the trial registry number before we can pass your paper to the next stage of review. Please include in the Methods section of your paper your reasons for not registering this study before enrolment of participants started. Please confirm that all related trials are registered by stating: “The authors confirm that all ongoing and related trials for this drug/intervention are registered”.

Please see http://journals.plos.org/plosone/s/submission-guidelines#loc-clinical-trials for our policies on clinical trials.

3. Please include captions for your Supporting Information files at the end of your manuscript, and update any in-text citations to match accordingly. Please see our Supporting Information guidelines for more information: http://journals.plos.org/plosone/s/supporting-information.

4. We note that the grant information you provided in the ‘Funding Information’ and ‘Financial Disclosure’ sections do not match.

When you resubmit, please ensure that you provide the correct grant numbers for the awards you received for your study in the ‘Funding Information’ section

5. Please include your full ethics statement in the ‘Methods’ section of your manuscript file. In your statement, please include the full name of the IRB or ethics committee who approved or waived your study, as well as whether or not you obtained informed written or verbal consent. If consent was waived for your study, please include this information in your statement as well.

6. If the reviewer comments include a recommendation to cite specific previously published works, please review and evaluate these publications to determine whether they are relevant and should be cited. There is no requirement to cite these works unless the editor has indicated otherwise.

[Note: HTML markup is below. Please do not edit.]

Reviewers' comments:

Reviewer's Responses to Questions

**Comments to the Author**

1. Is the manuscript technically sound, and do the data support the conclusions?

Reviewer #1: Yes

Reviewer #2: Yes

Reviewer #3: Partly

**********

2. Has the statistical analysis been performed appropriately and rigorously?

Reviewer #1: Yes

Reviewer #2: Yes

Reviewer #3: No

**********

3. Have the authors made all data underlying the findings in their manuscript fully available?

The PLOS Data policy

Reviewer #1: Yes

Reviewer #2: Yes

Reviewer #3: No

**********

4. Is the manuscript presented in an intelligible fashion and written in standard English?

Reviewer #1: Yes

Reviewer #2: No

Reviewer #3: No

**********

Reviewer #1: The trial registration date (2 April 2025) postdates the stated commencement of participant enrollment (22 January 2025). This means the trial was not prospectively registered before enrollment began, which represents a deviation from ICMJE requirements and PLOS ONE policy for clinical trials. The authors should address this discrepancy explicitly. If enrollment began before registration, this should be disclosed as a limitation and the authors should confirm that no changes were made to outcomes or analyses after registration (which they do state at line 108–109, though this statement was made in reference to design changes generally). This is a potentially significant issue that the journal editorial office may need to adjudicate.

Reviewer #2: GENERAL COMMENTS

I appreciate the opportunity to review manuscript #PONE-D-26-16084 titled “Effectiveness of Elastic Therapeutic Taping in Pain Management After Median Sternotomy: A Randomized Controlled Trial". I enjoyed reading this.

I have several comments and suggestions detailed in my specific comments that follow, that I believe will strengthen this work. I also note that some improvement in the writing of this manuscript in the English language is needed.

SPECIFIC COMMENTS

*Page 3 (Abstract) – In your Results, suggest that you state whether any of the differences you observed were considered to be clinically important.

*Page 3 (Keywords) – Suggest that you add “randomized controlled trial” as a keyword.

*Page 4, lines 93-95 (Introduction) – Suggest that you be more cautious with this statement and insert something like “To the best of the investigative team’s knowledge, no adequately powered, double blind…”

*Page 6, lines 125-126 (Methods, Inclusion and Exclusion Criteria) – Suggest that you tell the reader whether males and/or females were eligible for inclusion in the study.

*Page 7, line 164 (Methods, Functional Mobility) – For the TUG test, suggest that you insert the term “single-task” after the word “standardized”.

*Page 8, lines 192-193 (Methods, Statistical Analysis) – For an outcome such as the single-task TUG test this would traditionally be tested using a statistical test for continuous data (ANOVA, etc.) versus the Kruskal-Wallis test. I’m assuming that you used the Kruskal-Wallis test because the data for all three of these outcomes (sleep quality, fatigue, TUG test) were not normally distributed. If so, please make it clear to the reader as to why you used the Kruskal-Wallis test.

*Pages 8 and 9 (Methods, Statistical Analysis) – Beyond tests of statistical significance, was any consideration given to what differences might be clinically important? For example, a minimal clinically important difference of 10 to 15 mm (points) on a 0-100 Visual Analog Scale for pain.

*Pages 10-15 (Discussion) - For ease of reading, suggest that you partition your Discussion into the following subsections and address: (1) Overall findings, (2) Implications for research, (3) Implications for practice, and (4) Strengths and potential limitations. You can obviously have additional subsections listed under these.

*Page 10, line 238 (Discussion) – Suggest that you insert the term “3-arm” before the word “randomized”.

*Page 10-11 (Discussion, Pain Control and Underlying Mechanisms) – Please tell the reader what minimal clinically important difference you’re referring to here.

*Page 11, lines 257-259 (Discussion, Control and Underlying Mechanisms) – Since this was a 3-arm study, what two groups are you referring to here?

*Page 15, lines 352-360 (Limitations and Confounders) – First, at the beginning of this section, suggest that you tell the reader what the major strength(s) of this study was/were. Second, another limitation that should be mentioned is that the results of this study may not be generalizable to other populations.

END OF REVIEW

Reviewer #3: Thank you for the opportunity to review this manuscript entitled *“Effectiveness of Elastic Therapeutic Taping in Pain Management After Median Sternotomy: A Randomized Controlled Trial.”* The topic is clinically relevant, particularly given the need for non-pharmacological, opioid-sparing strategies after cardiac surgery. The randomized design, inclusion of both placebo and sham taping groups, and assessment of pain during coughing as well as at rest are notable strengths. The manuscript suggests potentially meaningful benefits of elastic therapeutic taping on postoperative pain, functional mobility, analgesic consumption, sleep quality, and length of hospital stay. However, several important methodological, statistical, reporting, and interpretation issues need to be addressed before the manuscript can be considered further.

I. Major Comments

1. Trial registration timing requires clarification

The manuscript states that recruitment began on January 22, 2025, while the trial was registered on ClinicalTrials.gov on April 2, 2025. This suggests that the trial may have been registered after participant enrollment had already started. The authors should explicitly clarify whether registration was prospective or retrospective. If any participants were enrolled before registration, this must be clearly stated in the Methods and discussed as a limitation.

2. The claim of intention-to-treat analysis is not fully supported

The manuscript reports that 203 participants were randomized, but only 195 were included in the final analysis. At the same time, the authors state that analyses were conducted according to the intention-to-treat principle and that no participants were excluded from the analysis. This is internally inconsistent. A true intention-to-treat analysis should include all randomized participants. The authors should clarify exactly which population was analyzed: intention-to-treat, modified intention-to-treat, per-protocol, or complete-case. The handling of the 7–8 participants lost to follow-up should be described more transparently.

3. Missing data handling needs more detail

The manuscript states that missing data were handled using last observation carried forward (LOCF), with similar results in complete-case sensitivity analyses. However, the extent, pattern, and timing of missing data are not sufficiently described. The authors should provide the number of missing observations per outcome and group, explain why LOCF was chosen, and present sensitivity analysis results either in the main text or supplementary material.

4. Randomization and allocation concealment are insufficiently described

The manuscript mentions a computer-generated block randomization sequence, but does not adequately describe allocation concealment. The authors should state who generated the random sequence, who enrolled participants, who assigned interventions, whether sealed opaque envelopes or a centralized system were used, and whether block sizes were fixed or varied. This is essential for assessing selection bias.

5. Blinding should be reported more rigorously

The study is described as double-blind, with participants and outcome assessors blinded. However, the active taping, placebo taping, and sham taping were applied to different anatomical regions or with different tension, which may have been distinguishable to participants. The authors should describe whether blinding success was assessed, for example by asking participants or assessors to guess group allocation. If blinding was not formally assessed, this should be acknowledged as a limitation.

6.The analgesic protocol appears inconsistent with the results

The Methods state that all participants received scheduled intravenous paracetamol 1000 mg, while Table 3 shows significantly lower total paracetamol use in the ETT group compared with control groups. If paracetamol was scheduled equally for all participants, it is unclear why total paracetamol differed substantially between groups. The authors should clarify whether paracetamol was truly scheduled, rescue-based, discontinued early, or adjusted according to clinical status. This point is important because analgesic consumption is a key secondary outcome.

7. VAS threshold for rescue tramadol should be corrected or clarified

The Methods state that rescue tramadol was administered when VAS ≥4. Since pain was measured using a 100-mm VAS, the threshold should presumably be ≥40 mm, unless the authors converted the scale to 0–10 for clinical decision-making. This should be clarified and standardized throughout the manuscript.

8. Statistical methods require reconsideration and clearer justification

The authors present many outcomes as medians and interquartile ranges, suggesting non-normal distributions, but the primary repeated pain outcome was analyzed using two-way mixed-effects ANOVA. The authors should justify the use of parametric repeated-measures analysis for VAS data or consider a more appropriate model, such as a mixed-effects model with robust methods or ordinal/nonparametric repeated-measures approaches. The manuscript should also clearly distinguish between mixed-effects ANOVA and mixed-effects modeling, as these terms are not interchangeable.

9. Multiple comparison correction is inconsistently applied

The authors state that length of hospital stay was excluded from multiple-comparison correction because it was a prespecified secondary outcome. Prespecification alone does not necessarily justify exclusion from multiplicity adjustment when multiple secondary outcomes are tested. The authors should provide a clearer statistical rationale and interpret secondary outcomes more cautiously.

10.Length of hospital stay may be confounded

The reported one-day reduction in hospital stay is clinically interesting, but discharge after CABG is influenced by many factors beyond pain and mobility, including surgical complications, arrhythmias, wound status, hemodynamic stability, social factors, and institutional discharge policies. The authors should report standardized discharge criteria and consider adjusted analyses for relevant clinical covariates. Without this, the conclusion that ETT shortened hospitalization should be stated more cautiously.

11. Cost-effectiveness claims are underdeveloped

The manuscript mentions a “simplified cost-effectiveness analysis,” but no methods, assumptions, cost inputs, or results are presented. Either a transparent economic analysis should be added, or the cost-effectiveness claims should be removed or substantially softened.

12. Clinical significance should be reported more completely

The authors state that pain reduction exceeded the minimal clinically important difference. This is useful, but the manuscript should report between-group differences with confidence intervals at each time point, especially for the primary outcome. Reporting only p-values limits the interpretation of clinical importance.

13. Adverse events and safety reporting need more detail

The manuscript states that no serious adverse events occurred and that skin reactions were monitored. The authors should provide the number and type of any minor adverse events by group, including redness, itching, irritation, tape intolerance, or early tape removal. If no adverse events occurred at all, this should be explicitly stated.

14. Data availability statement is not compliant with PLOS ONE expectations

The submission form contains a placeholder-style statement indicating that the minimal dataset is available at a repository via DOI, while the manuscript states that data are available from the corresponding author upon reasonable request. This inconsistency must be resolved. PLOS ONE generally expects the minimal dataset underlying the findings to be publicly available unless ethical or legal restrictions apply. The authors should provide a repository link/DOI or a clear justification for restricted access.

15. Figures require revision

The submitted figures appear cropped or difficult to read, particularly the CONSORT flow diagram and graphs. Figures should be uploaded in higher resolution, with complete legends, readable axis labels, clear group labels, and no cropped content. Figure 3 should clearly distinguish paracetamol and tramadol or explain whether it represents total analgesic dose, as combining medications with different dosing ranges may be misleading.

16. The discussion overstates causality in places

The manuscript attributes improvements to mechanoreceptive stimulation, biomechanical support, trunk stabilization, and proprioceptive mechanisms. While plausible, these mechanisms were not directly measured. The discussion should more clearly distinguish demonstrated clinical outcomes from speculative mechanisms.

17. The manuscript needs careful language editing

There are several grammatical and typographical errors, including “couging,” “withcompletion,” “haspital,” “PT Group),” inconsistent spacing, and awkward phrasing in the Discussion. A full professional language edit is recommended before resubmission.

II. Minor Comments

1. Please standardize terminology throughout the manuscript: “elastic therapeutic taping,” “Kinesio taping,” “elastic taping,” and “ETT” are used somewhat interchangeably.

2. Please ensure consistency in reporting country names: “Turkiye,” “Türkiye,” and “Turkey” should be standardized according to journal style.

3. The Abstract should be more cautious. For example, “shortens hospitalization” may be revised to “was associated with a shorter length of hospital stay.”

4. The Methods should specify whether outcomes were assessed by the same blinded assessor throughout or by multiple assessors, and whether inter-rater reliability was considered.

5. The sample size calculation should explain why Cohen’s d=0.49 was appropriate for a three-arm repeated-measures trial and whether attrition was accounted for.

6. Baseline sleep quality, fatigue, and anxiety are reported, but the Methods state that sleep quality and functional mobility were evaluated only at 48 hours. Please clarify which outcomes were measured at baseline and which only after intervention.

7. Please report whether any patients required ICU readmission, reoperation, or had postoperative complications, even if not primary outcomes.

8. Please clarify whether NSAID use was standardized or clinician-dependent.

9. Table formatting should be improved. Some values use brackets while others use parentheses, and superscript notation is not always clear.

10. Please provide confidence intervals for key effect estimates, including pain scores, TUG, tramadol use, and length of stay.

**********

what does this mean?). If published, this will include your full peer review and any attached files.

If you choose “no”, your identity will remain anonymous but your review may still be made public.

**Do you want your identity to be public for this peer review?**  For information about this choice, including consent withdrawal, please see our Privacy Policy

Reviewer #1: No

Reviewer #2: No

Reviewer #3: **Yes:**  Marwa Abass

**********

[NOTE: If reviewer comments were submitted as an attachment file, they will be attached to this email and accessible via the submission site. Please log into your account, locate the manuscript record, and check for the action link "View Attachments". If this link does not appear, there are no attachment files.]

To ensure your figures meet our technical requirements, please review our figure guidelines: https://journals.plos.org/plosone/s/figures

You may also use PLOS’s free figure tool, NAAS, to help you prepare publication quality figures: https://journals.plos.org/plosone/s/figures#loc-tools-for-figure-preparation.

NAAS will assess whether your figures meet our technical requirements by comparing each figure against our figure specifications.

Attachment Submitted filename: Review Report.docx

# Author comment: Author response to Decision Letter 1

**DOI:** 10.1371/journal.pone.0359282.r002

26 Jun 2026

We sincerely thank the Academic Editor and the reviewers for their careful evaluation of our manuscript and for their constructive comments and suggestions. We have carefully considered all comments and revised the manuscript accordingly. Detailed responses to each comment are provided below, and all corresponding changes have been incorporated into the revised manuscript.

Journal Requirements:

When submitting your revision, we need you to address these additional requirements.

1. Please ensure that your manuscript meets PLOS ONE's style requirements, including those for file naming. The PLOS ONE style templates can be found at

https://journals.plos.org/plosone/s/file?id=wjVg/PLOSOne_formatting_sample_main_body.pdf and https://journals.plos.org/plosone/s/file?id=ba62/PLOSOne_formatting_sample_title_authors_affiliations.pdf

Response: Thank you for this comment. The manuscript has been carefully revised to comply with PLOS ONE formatting and style requirements. The title page, author affiliations, section headings, manuscript formatting, and file naming conventions have been reviewed and updated in accordance with the PLOS ONE style templates.

2. PLOS ONE requires that all clinical trials are registered in an appropriate registry (the WHO list of approved registries is at https://www.who.int/clinical-trials-registry-platform/network/primary-registries" https://www.who.int/clinical-trials-registry-platform/network/primary-registries and more information on trial registration is at http://www.icmje.org/about-icmje/faqs/clinical-trials-registration/).

Response: Thank you for highlighting this important issue. The trial was registered at ClinicalTrials.gov (NCT06910215). Ethical approval was obtained on January 21, 2025, and participant recruitment began on January 22, 2025. The trial record was first released on March 25, 2025 and publicly posted on April 2, 2025. We acknowledge that the registration was retrospective. The delayed registration resulted from administrative procedures during trial setup. Importantly, no changes were made to the study design, eligibility criteria, interventions, outcomes, sample size calculation, or statistical analysis plan after participant enrollment began or after trial registration. This information has now been explicitly added to the Methods section and acknowledged in the Limitations section. We also confirm that all ongoing and related trials for this intervention are registered

3. Please include captions for your Supporting Information files at the end of your manuscript, and update any in-text citations to match accordingly. Please see our Supporting Information guidelines for more information: http://journals.plos.org/plosone/s/supporting-information.

Response: Thank you for this comment. Supporting Information captions have now been added at the end of the manuscript in accordance with the PLOS ONE Supporting Information guidelines. The supporting files were labeled sequentially (S1–S6) and their captions were included in the manuscript file.

4. We note that the grant information you provided in the ‘Funding Information’ and ‘Financial Disclosure’ sections do not match.

When you resubmit, please ensure that you provide the correct grant numbers for the awards you received for your study in the ‘Funding Information’ section

Response: Thank you for bringing this issue to our attention. We reviewed and standardized the funding information across the manuscript and submission system.

5. Please include your full ethics statement in the ‘Methods’ section of your manuscript file. In your statement, please include the full name of the IRB or ethics committee who approved or waived your study, as well as whether or not you obtained informed written or verbal consent. If consent was waived for your study, please include this information in your statement as well.

Response: Thank you for this comment. The ethics statement in the Methods section has been revised and expanded. We now provide the full name of the approving ethics committee (Bolu Abant Izzet Baysal University Non-Interventional Clinical Research Ethics Committee), the approval number and date, and clarification that written informed consent was obtained from all participants before enrollment.

6. If the reviewer comments include a recommendation to cite specific previously published works, please review and evaluate these publications to determine whether they are relevant and should be cited. There is no requirement to cite these works unless the editor has indicated otherwise.

Response: Thank you. No specific publications were recommended by the reviewers for citation.

Review Comments to the Author

We sincerely thank the reviewer for the valuable feedback. The comment has helped improve the clarity and transparency of the manuscript.

Reviewer #1: The trial registration date (2 April 2025) postdates the stated commencement of participant enrollment (22 January 2025). This means the trial was not prospectively registered before enrollment began, which represents a deviation from ICMJE requirements and PLOS ONE policy for clinical trials. The authors should address this discrepancy explicitly. If enrollment began before registration, this should be disclosed as a limitation and the authors should confirm that no changes were made to outcomes or analyses after registration (which they do state at line 108–109, though this statement was made in reference to design changes generally). This is a potentially significant issue that the journal editorial office may need to adjudicate.

Response: We thank the reviewer for identifying this important issue. We acknowledge that the study was registered retrospectively. Ethical approval was obtained before recruitment commenced (January 21, 2025), and participant enrollment began on January 22, 2025. The trial record was subsequently released on ClinicalTrials.gov on March 25, 2025 and publicly posted on April 2, 2025. The delay in registration was related to administrative procedures during trial setup. Importantly, no changes were made to the study design, eligibility criteria, interventions, outcomes, sample size calculation, or statistical analysis plan after participant enrollment began or after trial registration. This clarification has now been added to the Methods section and acknowledged as a limitation in the revised manuscript.

Reviewer #2: GENERAL COMMENTS

I appreciate the opportunity to review manuscript #PONE-D-26-16084 titled “Effectiveness of Elastic Therapeutic Taping in Pain Management After Median Sternotomy: A Randomized Controlled Trial". I enjoyed reading this.

I have several comments and suggestions detailed in my specific comments that follow, that I believe will strengthen this work. I also note that some improvement in the writing of this manuscript in the English language is needed.

Response: We sincerely thank the reviewer for the constructive comments and thoughtful suggestions, which have helped improve the quality and clarity of the manuscript.

SPECIFIC COMMENTS

*Page 3 (Abstract) – In your Results, suggest that you state whether any of the differences you observed were considered to be clinically important.

Response: Thank you for this valuable suggestion. We agree that reporting clinical relevance alongside statistical significance improves the interpretation of the findings. Therefore, the Abstract Results section has been revised to indicate that the observed reduction in pain was clinically meaningful and exceeded the minimal clinically important difference reported for postoperative pain.

*Page 3 (Keywords) – Suggest that you add “randomized controlled trial” as a keyword.

Response: Thank you for this suggestion. We have added “randomized controlled trial” to the list of keywords.

*Page 4, lines 93-95 (Introduction) – Suggest that you be more cautious with this statement and insert something like “To the best of the investigative team’s knowledge, no adequately powered, double blind…”

Response: Thank you for this suggestion. We agree that a more cautious wording is appropriate. Accordingly, the statement has been revised to read: “To the best of the investigators’ knowledge, no adequately powered, double-blind randomized trial has comprehensively evaluated the opioid-sparing and functional recovery effects of sternum-targeted elastic taping following CABG.”

*Page 6, lines 125-126 (Methods, Inclusion and Exclusion Criteria) – Suggest that you tell the reader whether males and/or females were eligible for inclusion in the study.

Response: Thank you for this comment. We have clarified the inclusion criteria by explicitly stating that both male and female patients were eligible for participation in the study.

*Page 7, line 164 (Methods, Functional Mobility) – For the TUG test, suggest that you insert the term “single-task” after the word “standardized”.

Response: Thank you for this suggestion. We have revised the Methods section to clarify that functional mobility was assessed using the standardized single-task Timed Up and Go (TUG) test.

*Page 8, lines 192-193 (Methods, Statistical Analysis) – For an outcome such as the single-task TUG test this would traditionally be tested using a statistical test for continuous data (ANOVA, etc.) versus the Kruskal-Wallis test. I’m assuming that you used the Kruskal-Wallis test because the data for all three of these outcomes (sleep quality, fatigue, TUG test) were not normally distributed. If so, please make it clear to the reader as to why you used the Kruskal-Wallis test.

Response: Thank you for this comment. We used the Kruskal-Wallis test because TUG, sleep quality, and length of hospital stay data did not meet the assumption of normality according to the Shapiro-Wilk test and were therefore summarized as median (interquartile range). To improve clarity, we have revised the Statistical Analysis section to explicitly state the rationale for using the Kruskal-Wallis test for these outcomes.

*Pages 8 and 9 (Methods, Statistical Analysis) – Beyond tests of statistical significance, was any consideration given to what differences might be clinically important? For example, a minimal clinically important difference of 10 to 15 mm (points) on a 0-100 Visual Analog Scale for pain.

Response: Thank you for this important comment. Clinical importance was not formally incorporated into the prespecified statistical analysis plan. However, we agree that interpretation of the findings should consider both statistical and clinical significance. Therefore, we have revised the Discussion section to relate the observed pain reduction to the published minimal clinically important difference (MCID) of 9.9 mm for acute postoperative pain measured on a 100-mm visual analog scale. The approximately 20–22 mm reduction observed in the ETT group exceeded this threshold, supporting the clinical relevance of the findings.

*Pages 10-15 (Discussion) - For ease of reading, suggest that you partition your Discussion into the following subsections and address: (1) Overall findings, (2) Implications for research, (3) Implications for practice, and (4) Strengths and potential limitations. You can obviously have additional subsections listed under these.

Response: Thank you for this helpful suggestion. To improve readability and facilitate interpretation, we reorganized the Discussion section by introducing clearer subsections addressing the overall findings, implications for practice, and strengths and limitations of the study. We believe this revised structure improves the flow and accessibility of the Discussion

*Page 10, line 238 (Discussion) – Suggest that you insert the term “3-arm” before the word “randomized”.

Response: Thank you for this suggestion. We have revised the sentence in the Discussion section to specify that this was a 3-arm randomized controlled study

*Page 10-11 (Discussion, Pain Control and Underlying Mechanisms) – Please tell the reader what minimal clinically important difference you’re referring to here.

Response: Thank you for this comment. We have clarified the statement by specifying the minimal clinically important difference (MCID) to which we referred. The Discussion section now states that the approximately 20–22 mm reduction in VAS scores observed in the ETT group exceeded the published MCID of 9.9 mm for acute postoperative pain measured on a 100-mm visual analog scale, thereby supporting the clinical relevance of the findings.

*Page 11, lines 257-259 (Discussion, Control and Underlying Mechanisms) – Since this was a 3-arm study, what two groups are you referring to here?

Response: Thank you for this comment. We agree that the original wording was ambiguous in the context of a 3-arm study. We have revised the sentence to explicitly state that no significant differences were observed between the placebo and sham groups, thereby clarifying the comparison being referenced.

*Page 15, lines 352-360 (Limitations and Confounders) – First, at the beginning of this section, suggest that you tell the reader what the major strength(s) of this study was/were. Second, another limitation that should be mentioned is that the results of this study may not be generalizable to other populations.

Response: Thank you for this helpful suggestion. We have revised the section to begin with the major strengths of the study, including its three-arm randomized design, placebo and sham control groups, and a priori sample size calculation. We have also added a statement acknowledging that the single-center design may limit the generalizability of the findings to other healthcare settings and patient populations.

END OF REVIEW

Reviewer #3: Thank you for the opportunity to review this manuscript entitled *“Effectiveness of Elastic Therapeutic Taping in Pain Management After Median Sternotomy: A Randomized Controlled Trial.”* The topic is clinically relevant, particularly given the need for non-pharmacological, opioid-sparing strategies after cardiac surgery. The randomized design, inclusion of both placebo and sham taping groups, and assessment of pain during coughing as well as at rest are notable strengths. The manuscript suggests potentially meaningful benefits of elastic therapeutic taping on postoperative pain, functional mobility, analgesic consumption, sleep quality, and length of hospital stay. However, several important methodological, statistical, reporting, and interpretation issues need to be addressed before the manuscript can be considered further.

Response: We appreciate the reviewer’s detailed and constructive evaluation. All comments have been carefully considered and addressed in the revised manuscript.

I. Major Comments

1. Trial registration timing requires clarification

The manuscript states that recruitment began on January 22, 2025, while the trial was registered on ClinicalTrials.gov on April 2, 2025. This suggests that the trial may have been registered after participant enrollment had already started. The authors should explicitly clarify whether registration was prospective or retrospective. If any participants were enrolled before registration, this must be clearly stated in the Methods and discussed as a limitation.

Response: We appreciate the reviewer’s comment. We acknowledge that the trial was registered retrospectively. Ethical approval was obtained on January 21, 2025, participant recruitment started on January 22, 2025, the trial record was first released on March 25, 2025, and publicly posted on April 2, 2025. The delayed registration resulted from administrative procedures during trial setup. No modifications were made to the study design, eligibility criteria, interventions, outcome measures, sample size calculation, or statistical analysis plan after recruitment had started or after registration. To improve transparency, this information has been explicitly incorporated into the Methods section and discussed in the Limitations section of the revised manuscript.

2. The claim of intention-to-treat analysis is not fully supported

The manuscript reports that 203 participants were randomized, but only 195 were included in the final analysis. At the same time, the authors state that analyses were conducted according to the intention-to-treat principle and that no participants were excluded from the analysis. This is internally inconsistent. A true intention-to-treat analysis should include all randomized participants. The authors should clarify exactly which population was analyzed: intention-to-treat, modified intention-to-treat, per-protocol, or complete-case. The handling of the 7–8 participants lost to follow-up should be described more transparently.

Response: Thank you for this important comment. We agree that the previous wording was inconsistent. Although 203 participants were randomized, 195 participants who received the allocated intervention and had evaluable post-intervention outcome data were included in the primary analysis. Therefore, the analysis population is more accurately described as a modified intention-to-treat population rather than a full intention-to-treat population. We have revised the manuscript accordingly and removed the statement that “no participants were excluded from the analysis.” We also clarified the number and reasons for loss to follow-up in each group and described the handling of missing data more transparently.

3. Missing data handling needs more detail

The manuscript states that missing data were handled using last observation carried forward (LOCF), with similar results in complete-case sensitivity analyses. However, the extent, pattern, and timing of missing data are not sufficiently described. The authors should provide the number of missing observations per outcome and group, explain why LOCF was chosen, and present sensitivity analysis results either in the main text or supplementary material.

Response: Thank you for this comment. We agree that the description of missing data handling required clarification. Seven participants were lost to follow-up after randomization (ETT, n=2; PT, n=3; ST, n=2) and therefore did not contribute post-intervention outcome data. The final analysis was conducted on the 195 participants with available outcome data. We have revised the manuscript to clearly describe the number of participants lost to follow-up in each group and removed statements that could imply formal imputation procedures.

4. Randomization and allocation concealment are insufficiently described

The manuscript mentions a computer-generated block randomization sequence, but does not adequately describe allocation concealment. The authors should state who generated the random sequence, who enrolled participants, who assigned interventions, whether sealed opaque envelopes or a centralized system were used, and whether block sizes were fixed or varied. This is essential for assessing selection bias.

Response: Thank you for this valuable comment. We have revised the Methods section to provide additional details regarding the randomization procedure. Specifically, we clarified that a computer-generated block randomization sequence was created using the Blockrand package in R prior to participant recruitment, using fixed blocks of six participants. We also specified that eligible participants were enrolled by the cardiovascular surgery team, that intervention assignments followed the pre-generated allocation sequence, and that participants and outcome assessors remained blinded throughout the study.

5. Blinding should be reported more rigorously

The study is described as double-blind, with participants and outcome assessors blinded. However, the active taping, placebo taping, and sham taping were applied to different anatomical regions or with different tension, which may have been distinguishable to participants. The authors should describe whether blinding success was assessed, for example by asking participants or assessors to guess group allocation. If blinding was not formally assessed, this should be acknowledged as a limitation.

Response: Thank you for this comment. Blinding success was not formally assessed in this study. This limitation has now been acknowledged in the Discussion section.

6.The analgesic protocol appears inconsistent with the results

The Methods state that all participants received scheduled intravenous paracetamol 1000 mg, while Table 3 shows significantly lower total paracetamol use in the ETT group compared with control groups. If paracetamol was scheduled equally for all participants, it is unclear why total paracetamol differed substantially between groups. The authors should clarify whether paracetamol was truly scheduled, rescue-based, discontinued early, or adjusted according to clinical status. This point is important because analgesic consumption is a key secondary outcome.

Response: Thank you for this comment. Intravenous paracetamol was included in the standard postoperative analgesic protocol; however, total analgesic consumption was not fixed and depended on individual analgesic requirements during the postoperative period. We have clarified this point in the Methods section.

7. VAS threshold for rescue tramadol should be corrected or clarified

The Methods state that rescue tramadol was administered when VAS ≥4. Since pain was measured using a 100-mm VAS, the threshold should presumably be ≥40 mm, unless the authors converted the scale to 0–10 for clinical decision-making. This should be clarified and standardized throughout the manuscript.

Response: Thank you for this comment. We agree that the wording was unclear. Pain intensity was assessed using a 100-mm visual analog scale (VAS), and rescue tramadol was administered when the VAS score was ≥40 mm. The Methods section has been revised accordingly for consistency throughout the manuscript.

8. Statistical methods require reconsideration and clearer justification

The authors present many outcomes as medians and interquartile ranges, suggesting non-normal distributions, but the primary repeated pain outcome was analyzed using two-way mixed-effects ANOVA. The authors should justify the use of parametric repeated-measures analysis for VAS data or consider a more appropriate model, such as a mixed-effects model with robust methods or ordinal/nonparametric repeated-measures approaches. The manuscript should also clearly distinguish between mixed-effects ANOVA and mixed-effects modeling, as these terms are not interchangeable.

Response: Thank you for this comment. The primary outcome (VAS pain scores) was analyzed using a two-way mixed ANOVA because the study was designed to evaluate group, time, and group-by-time interaction effects across repeated measurements. Although some secondary outcomes did not meet normality assumptions and were therefore analyzed using non-parametric methods, the VAS analysis was performed using a parametric repeated-measures approach. Given the balanced design, equal group sizes, and sample size of 65 participants per group, mixed ANOVA was considered sufficiently robust to moderate departures from normality. To avoid ambiguity, the term “two-way mixed ANOVA” has been used consistently throughout the manuscript.

9. Multiple comparison correction is inconsistently applied

The authors state that length of hospital stay was excluded from multiple-comparison correction because it was a prespecified secondary outcome. Prespecification alone does not necessarily justify exclusion from multiplicity adjustment when multiple secondary outcomes are tested. The authors should provide a clearer statistical rationale and interpret secondary outcomes more cautiously.

Response: Thank you for this comment. We agree that prespecification alone does not eliminate concerns regarding multiplicity. Therefore, we have removed this statement and interpreted secondary outcomes, including length of hospital stay, as supportive findings that should be interpreted with appropriate caution.

10.Length of hospital stay may be confounded

The reported one-day reduction in hospital stay is clinically interesting, but discharge after CABG is influenced by many factors beyond pain and mobility, including surgical complications, arrhythmias, wound status, hemodynamic stability, social factors, and institutional discharge policies. The authors should report standardized discharge criteria and consider adjusted analyses for relevant clinical covariates. Without this, the conclusion that ETT shortened hospitalization should be stated more cautiously.

Response: Thank you for this comment. We agree that length of hospital stay may be influenced by multiple clinical and non-clinical factors beyond pain and functional recovery. Standard postoperative discharge decisions were made by the treating cardiovascular surgery team according to routine institutional practice. As detailed data on all potential determinants of discharge were not collected, adjusted analyses were not performed. We have revised the Discussion section to interpret the observed reduction in length of stay more cautiously.

11. Cost-effectiveness claims are underdeveloped

The manuscript mentions a “simplified cost-effectiveness analysis,” but no methods, assumptions, cost inputs, or results are presented. Either a transparent economic analysis should be added, or the cost-effectiveness claims should be removed or substantially softened.

Response: Thank you for this comment. We agree that the manuscript did not include a formal economic evaluation with predefined cost inputs, assumptions, or cost-effectiveness outcomes. Therefore, we have removed references to a cost-effectiveness analysis and revised the manuscript to limit discussion to the observed reductions in analgesic consumption and length of hospital stay.

12. Clinical significance should be reported more completely

The authors state that pain reduction exceeded the minimal clinically important difference. This is useful, but the manuscript should report between-group differences with confidence intervals at each time point, especially for the primary outcome. Reporting only p-values limits the interpretation of clinical importance.

Response: Thank you for this comment. We agree that confidence intervals provide important information regarding the magnitude and precision of treatment effects. We have now reported between-group differences with 95% confidence intervals for the primary pain outcome at each postoperative assessment time point.

13. Adverse events and safety reporting need more detail

The manuscript states that no serious adverse events occurred and that skin reactions were monitored. The authors should provide the number and type of any minor adverse events by group, including redness, itching, irritation, tape intolerance, or early tape removal. If no adverse events occurred at all, this should be explicitly stated.

Response: Thank you for this comment. We have revised the manuscript to provide more detailed safety information. No serious adverse events occurred in any group. Minor adverse events were limited to two cases of mild skin redness in the ETT group and one case of mild itching in the placebo group. No adverse events were reported in the sham group. All reactions were transient, did not require medical treatment, and did not result in premature tape removal or study withdrawal.

14. Data availability statement is not compliant with PLOS ONE expectations

The submission form contains a placeholder-style statement indicating that the minimal dataset is available at a repository via DOI, while the manuscript states that data are available from the corresponding author upon reasonable request. This inconsistency must be resolved. PLOS ONE generally expects the minimal dataset underlying the findings to be publicly available unless ethical or legal restrictions apply. The authors should provide a repository link/DOI or a clear justification for restricted access.

Response: Thank you for this comment. We have revised the Data Availability Statement to ensure consistency throughout the manuscript and submission materials. The anonymized minimal dataset underlying the findings of this study is now publicly available in the Zenodo repository (https://doi.org/10.5281/zenodo.20922725).

15. Figures require revision

The submitted figures appear cropped or difficult to read, particularly the CONSORT flow diagram and graphs. Figures should be uploaded in higher resolution, with complete legends, readable axis labels, clear group labels, and no cropped content. Figure 3 should clearly distinguish paracetamol and tramadol or explain whether it represents total analgesic dose, as combining medications with different dosing ranges may be misleading.

Response: Thank you for your valuable comment. The CONSORT flow diagram and all figures have been regenerated in high resolution (600 dpi) with improved readability, including larger axis labels, clearer group labels, and complete figure legends. In addition, Figure 3 has been revised to present paracetamol and tramadol consumption separately in two panels to avoid potential misinterpretation due to their different dosing ranges.

16. The discussion overstates causality in places

The manuscript attributes improvements to mechanoreceptive stimulation, biomechanical support, trunk stabilization, and proprioceptive mechanisms. While plausible, these mechanisms were not directly measured. The discussion should more clearly distinguish demonstrated clinical outcomes from speculative mechanisms.

Response: Thank you for this valuable comment. The Discussion has been revised to more clearly distinguish the observed clinical outcomes from the proposed mechanisms of action. We have softened statements implying causality and explicitly clarified that the neurophysiological and biomechanical mechanisms discussed are biologically plausible but were not directly evaluated in the present study.

17. The manuscript needs careful language editing

There are several grammatical and typographical errors, including “couging,” “withcompletion,” “haspital,” “PT Group),” inconsistent spacing, and awkward phrasing in the Discussion. A full professional language edit is recommended before resubmission.

Response: Thank you for your careful review. The manuscript has been thoroughly edited to correct grammatical, typographical, and formatting errors, including those highlighted by the reviewer. We also carefully revised the wording throughout the manuscript to improve clarity, consistency, and overall readability.

II. Minor Comments

1. Please standardize terminology throughout the manuscript: “elastic therapeutic taping,” “Kinesio taping,” “elastic taping,” and “ETT” are used somewhat interchangeably.

Response: Thank you for this helpful suggestion. We have standardized the terminology throughout the manuscript. The intervention is now consistently referred to as elastic therapeutic taping (ETT) after its first definition, and inconsistent terms such as "Kinesio taping" and "elastic taping" have been replaced where appropriate.

2. Please ensure consistency in reporting country names: “Turkiye,” “Türkiye,” and “Turkey” should be standardized according to journal style.

Response: Thank you for this comment. All occurrences have been revised to "Turkey" for consistency.

3. The Abstract should be more cautious. For example, “shortens hospitalization” may be revised to “was associated with a shorter length of hospital stay.”

Response: Thank you for this helpful suggestion. We have revised the Abstract to use more cautious language. Statements implying causality have been replaced with associative wording, including revising "shortens hospitalization" to "was associated with a shorter length of hospital stay.

4. The Methods should specify whether outcomes were assessed by the same blinded assessor throughout or by multiple assessors, and whether inter-rater reliability was considered.

Response: Thank you for this important comment. We have clarified the Methods to specify that all outcome assessments were performed by the same physiotherapist throughout the study, while the intervention was administered by a different physiotherapist. Because all measurements were obtained by the same assessor, inter-rater reliability was not applicable.

5. The sample size calculation should explain why Cohen’s d=0.49 was appropriate for a three-arm repeated-measures trial and whether attrition was accounted for.

Response: Thank you for this comment. We have revised the sample size calculation to clarify the rationale for the selected effect size and to specify that participant recruitment continued until the target sample size was achieved despite losses during follow-up.

6. Baseline sleep quality, fatigue, and anxiety are reported, but the Methods state that sleep quality and functional mobility were evaluated only at 48 hours. Please clarify which outcomes were measured at baseline and which only after intervention.

Response: Thank you for this comment. We have clarified the Methods to specify the assessment time points for each outcome, including baseline and post-intervention measurements, to ensure consistency with the reported results.

7. Please report whether any patients required ICU readmission, reoperation, or had postoperative complications, even if not primary outcomes.

Response: Thank you for this valuable comment. We have added a statement to the Results section indicating that no patients required ICU readmission or reoperation, and no major postoperative complications were observed during the study period.

8. Please clarify whether NSAID use was standardized or clinician-dependent.

Response: Thank you for this comment. We have clarified in the Methods that NSAID administration was not standardized and was left to the discretion of the treating physician according to routine clinical practice

9. Table formatting should be improved. Some values use brackets while others use parentheses, and superscript notation is not always clear.

Response: Thank you for this comment. We have revised the tables to ensure consistent formatting, notation, and presentation throughout the manuscript.

10. Please provide confidence intervals for key effect estimates, including pain scores, TUG, tramadol use, and length of stay.

Response: Thank you for this valuable suggestion. We have revised the Results section to include 95% confidence intervals for the primary pain outcomes and key secondary effect estimates, where appropriate.

Attachment Submitted filename: Response to Reviewers.docx

# Peer review: Decision Letter 1

**DOI:** 10.1371/journal.pone.0359282.r003

**Authors:** Burak Katipoğlu

8 Jul 2026

Dear Dr. Özel,

Thank you for submitting your manuscript to PLOS ONE. After careful consideration, we feel that it has merit but does not fully meet PLOS ONE’s publication criteria as it currently stands. Therefore, we invite you to submit a revised version of the manuscript that addresses the points raised during the review process.

Please submit your revised manuscript by Aug 22 2026 11:59PM. If you will need more time than this to complete your revisions, please reply to this message or contact the journal office at plosone@plos.org. When you're ready to submit your revision, log on to https://www.editorialmanager.com/pone/ and select the 'Submissions Needing Revision' folder to locate your manuscript file.

- A letter that responds to each point raised by the academic editor and reviewer(s). You should upload this letter as a separate file labeled 'Response to Reviewers'.
- A marked-up copy of your manuscript that highlights changes made to the original version. You should upload this as a separate file labeled 'Revised Manuscript with Track Changes'.
- An unmarked version of your revised paper without tracked changes. You should upload this as a separate file labeled 'Manuscript'.

If you would like to make changes to your financial disclosure, please include your updated statement in your cover letter. Guidelines for resubmitting your figure files are available below the reviewer comments at the end of this letter.

If applicable, we recommend that you deposit your laboratory protocols in protocols.io to enhance the reproducibility of your results. Protocols.io assigns your protocol its own identifier (DOI) so that it can be cited independently in the future. For instructions see: https://journals.plos.org/plosone/s/submission-guidelines#loc-laboratory-protocols. Additionally, PLOS ONE offers an option for publishing peer-reviewed Lab Protocol articles, which describe protocols hosted on protocols.io. Read more information on sharing protocols at https://plos.org/protocols?utm_medium=editorial-email&utm_source=authorletters&utm_campaign=protocols.

As the corresponding author, your ORCID iD is verified in the submission system and will appear in the published article. PLOS supports the use of ORCID, and we encourage all coauthors to register for an ORCID iD and use it as well. Please encourage your coauthors to verify their ORCID iD within the submission system before final acceptance, as unverified ORCID iDs will not appear in the published article. *Only*  the individual author can complete the verification step; PLOS staff *cannot*  verify ORCID iDs on behalf of authors.

We look forward to receiving your revised manuscript.

Kind regards,

Burak Katipoğlu

Academic Editor

PLOS One

Journal Requirements:

If the reviewer comments include a recommendation to cite specific previously published works, please review and evaluate these publications to determine whether they are relevant and should be cited. There is no requirement to cite these works unless the editor has indicated otherwise.

Additional Editor Comments:

Dear Authors,

Your revised manuscript on elastic therapeutic taping after median sternotomy has undergone peer review. The evaluation identified several methodological and reporting issues that require attention before the work can be reconsidered. First, the analysis of the primary pain outcome is not described consistently throughout the text. You should state plainly which method provides the primary between-group comparison and how that choice fits the way the data are presented. Second, the manuscript departs from the missing-data handling and analysis plan set out in the registered protocol, and these departures should be reconciled and justified. In addition, concealment of the randomization sequence and the way blinding was maintained are not sufficiently described in the main text. As the manuscript now stands, the risk of selection and assessment bias cannot be judged. Several interpretations in the discussion, in particular the causal reading of the hospital-stay difference, go beyond what the design supports and require more cautious wording that acknowledges confounding. The repeated and conflicting passages in the text should also be resolved. The decision is major revision. These issues arise not from the design itself but from the consistency of the analysis and reporting. Once they have been addressed, a revised version may be submitted.

Sincerely,

[Note: HTML markup is below. Please do not edit.]

Reviewers' comments:

Reviewer's Responses to Questions

**Comments to the Author**

Reviewer #4: (No Response)

Reviewer #5: All comments have been addressed

Reviewer #6: All comments have been addressed

**********

2. Is the manuscript technically sound, and do the data support the conclusions?

Reviewer #4: Partly

Reviewer #5: Yes

Reviewer #6: Yes

**********

3. Has the statistical analysis been performed appropriately and rigorously?

Reviewer #4: Yes

Reviewer #5: No

Reviewer #6: Yes

**********

4. Have the authors made all data underlying the findings in their manuscript fully available?

The PLOS Data policy

Reviewer #4: Yes

Reviewer #5: No

Reviewer #6: Yes

**********

5. Is the manuscript presented in an intelligible fashion and written in standard English?

Reviewer #4: No

Reviewer #5: Yes

Reviewer #6: Yes

**********

Reviewer #4: Thank you very much for your valuable work. Please address all the comments in detail.

Abstract

1. Please provide a detailed statistical analysis.

2. In lines 42-44, the observed between-group reduction in pain exceeded the reported minimal clinically important difference for acute postoperative pain, indicating a clinically meaningful treatment effect. Please specify the MCID value.

3. Add the statistical value for the TUG and sleep quality.

4. Include the clinical trial registry number at the end of the abstract.

Introduction

5. Define ETT and elaborate on its physiological and biomechanical function.

6. Explain additional therapeutic management for coronary artery bypass grafting (CABG) patients following immediate postoperative care and prior to discharge.

7. In line 74, add the word 'objective' or 'aim' of the study.

Materials and methods

8. Specify the study setting and period in accordance with the study design.

9. Provide details on the randomization sequence and blinding in a separate heading.

10. Include a link to the clinical trial registration details after the registry number.

11. In line 115, expand on the baseline assessment.

12. Clarify the inclusion criteria.

13. Explain the duration of taping applications for all three groups.

14. Add images of all taping procedures in accordance with ethical guidelines.

15. In line 185, elaborate on the length of stay (LOS).

16. Include validity and reliability information for all outcome parameters and interventions, citing recent references.

Results and Discussion

17. Separate the results and discussion into distinct headings.

18. Table 1 separates the numbers of male and female participants but omits surgical data when information is missing.

19. Please add the p-value in Table 1.

20. In line 362, the statement that effective early pain control may limit central sensitization and reduce the risk of chronic pain is supported. Please add a reference for the above sentence.

21. In lines 369-371, the marked reduction in total analgesic and tramadol consumption in the ETT group confirms that the intervention not only alters subjective pain perception but also reduces objective medication requirements [6]. How do the authors ascertain that pain reduction is solely due to the elastic taping method?

22. In lines 417-418, the claim that ETT may indirectly improve sleep by reducing pain and stabilizing the chest wall needs a supporting reference.

Reviewer #5: This revision addresses the vast majority of concerns raised during peer review. The manuscript is methodologically stronger, better written, and considerably more transparent than the original submission. The remaining issues are relatively minor and relate primarily to reporting clarity rather than fundamental flaws in the study design or results.

1. Modified intention-to-treat terminology still needs refinement: The authors now state that a modified intention-to-treat (mITT) analysis was performed, which is more appropriate than the previous ITT claim. However, the manuscript states:

"including all randomized participants who received the allocated intervention and had evaluable post-intervention outcome data"

This is not a standard mITT definition, since patients without outcome data were excluded entirely.

The authors should:

• explicitly define mITT according to CONSORT recommendations;

• explain why no imputation was performed;

• clarify that the analysis is essentially a complete-case modified ITT.

2. Randomization and allocation concealment remain incompletely described

The revision improves the description of randomization, but allocation concealment is still insufficiently reported. The manuscript states:

Group assignments were implemented according to the pre-generated sequence.

This does not explain:

• Were opaque-sealed envelopes used?

• Was allocation concealed electronically?

• Who had access to the randomization list?

• Could recruiters predict upcoming allocations?

Without this information, selection bias cannot be fully assessed.

3. Blinding remains potentially vulnerable

The authors acknowledge that blinding success was not assessed.

This is appropriate. However, the manuscript should discuss that:

• active taping was applied around the sternotomy,

• sham taping was applied on the back,

meaning participants may have recognized group allocation.

This limitation deserves slightly stronger emphasis.

4. Statistical analysis requires additional justification: The authors justify mixed ANOVA by citing robustness. However, VAS values throughout the Results are reported as median (IQR) rather than means. This creates an inconsistency.

If ANOVA was used, authors should explain:

• whether residuals were normally distributed;

• whether assumptions were checked on model residuals rather than raw data;

• why means are not presented for the primary endpoint.

Alternatively, mixed-effects regression would be statistically stronger.

5. Hospital stay remains vulnerable to confounding

The Discussion has become more cautious, which is appreciated.

However, the Results still state:

participants in the ETT group were discharged approximately one day earlier

This wording still implies causality.

Suggested wording:

"Participants receiving ETT had a median hospital stay one day shorter than control groups; however, discharge timing may have been influenced by factors beyond the intervention."

6. Table 2 inconsistency: Length of hospital stay was not included in the multiple-comparison adjustment. Yet the response letter states this sentence was removed.

Please ensure the table matches the revised statistical approach.

7. Sample size justification: The sample size is based on Cohen's d = 0.49 from King et al. It would strengthen reproducibility if the manuscript briefly explained:

• which outcome from King et al. generated this estimate;

• whether it represents between-group or within-group effect size.

8. Confidence intervals

Confidence intervals have now been added for several outcomes, which is excellent. Consider also reporting:

• confidence intervals for LOS

• confidence intervals for sleep quality

to maintain consistency across secondary outcomes.

9. Correlation analysis

The correlation analysis appears exploratory. The manuscript should clearly state:

"These analyses were exploratory and hypothesis-generating." Otherwise readers may overinterpret them.

10. Language: English has improved considerably.

Only a few small issues remain.

Examples: "with scored recorded" should be "with scores recorded"

"Participant recruitment continued until the predetermined sample size of 195 participants who completed the study was achieved." This sentence could be smoother.

Suggested: "Recruitment continued until the target number of 195 evaluable participants was achieved."

11. CONSORT flow Ensure Figure 1 clearly distinguishes:

• randomized

• received intervention

• lost to follow-up

• analyzed (modified ITT)

using standard CONSORT terminology.

12. Abstract: The abstract is much improved.

However, "Functional mobility and sleep quality improved." should specify "compared with placebo and sham groups" to avoid ambiguity.

Reviewer #6: Based on the reviewed revision manuscript, the article is fully suitable for publication in terms of scientific depth, statistical accuracy, methodological strength, transparency, and compliance with the open data policy. The authors have responded to all major and minor criticisms (mITT conversion, statistical justifications, removal of speculative statements, quality of visuals) from the editor and the three reviewers in a highly professional and satisfactory manner. The only potential risk facing the article, the fact that the study was recorded retrospectively, is explicitly acknowledged as a limitation in the revision text, and since the data is transparently disclosed in Zenodo, it will not pose an obstacle. It is anticipated that the journal editorial board will approve this revision and the article will be accepted and published. This is a highly successful revision manuscript with a very high chance of publication.

**********

what does this mean?). If published, this will include your full peer review and any attached files.

If you choose “no”, your identity will remain anonymous but your review may still be made public.

**Do you want your identity to be public for this peer review?**  For information about this choice, including consent withdrawal, please see our Privacy Policy

Reviewer #4: No

Reviewer #5: No

Reviewer #6: **Yes:**  Esedullah AKARAS

**********

[NOTE: If reviewer comments were submitted as an attachment file, they will be attached to this email and accessible via the submission site. Please log into your account, locate the manuscript record, and check for the action link "View Attachments". If this link does not appear, there are no attachment files.]

To ensure your figures meet our technical requirements, please review our figure guidelines: https://journals.plos.org/plosone/s/figures

You may also use PLOS’s free figure tool, NAAS, to help you prepare publication quality figures: https://journals.plos.org/plosone/s/figures#loc-tools-for-figure-preparation.

NAAS will assess whether your figures meet our technical requirements by comparing each figure against our figure specifications.

Attachment Submitted filename: PONE-D-26-16084R1.docx

# Author comment: Author response to Decision Letter 2

**DOI:** 10.1371/journal.pone.0359282.r004

10 Jul 2026

We sincerely thank the Academic Editor and the reviewers for their careful evaluation of our manuscript and for their constructive comments and valuable suggestions. We have carefully considered all comments and revised the manuscript accordingly. Detailed responses to each comment are provided below, and all corresponding changes have been incorporated into the revised manuscript.

Additional Editor Comments:

Dear Authors,

Your revised manuscript on elastic therapeutic taping after median sternotomy has undergone peer review. The evaluation identified several methodological and reporting issues that require attention before the work can be reconsidered. First, the analysis of the primary pain outcome is not described consistently throughout the text. You should state plainly which method provides the primary between-group comparison and how that choice fits the way the data are presented. Second, the manuscript departs from the missing-data handling and analysis plan set out in the registered protocol, and these departures should be reconciled and justified. In addition, concealment of the randomization sequence and the way blinding was maintained are not sufficiently described in the main text. As the manuscript now stands, the risk of selection and assessment bias cannot be judged. Several interpretations in the discussion, in particular the causal reading of the hospital-stay difference, go beyond what the design supports and require more cautious wording that acknowledges confounding. The repeated and conflicting passages in the text should also be resolved. The decision is major revision. These issues arise not from the design itself but from the consistency of the analysis and reporting. Once they have been addressed, a revised version may be submitted.

Sincerely,

Response: We thank the Academic Editor for the thoughtful and constructive comments. We have carefully revised the manuscript to address all of the issues raised. Specifically, we clarified the statistical approach for the primary outcome, reconciled and justified the differences between the registered protocol and the final analysis, expanded the description of randomization and blinding procedures, revised the discussion to avoid causal interpretation of the hospital length of stay findings, and corrected repeated or inconsistent statements throughout the manuscript. Detailed responses to each comment are provided below.

Review Comments to the Author

Reviewer #4: Thank you very much for your valuable work. Please address all the comments in detail.

Abstract

1. Please provide a detailed statistical analysis.

Response: Thank you for this valuable comment. We have revised the statistical analysis in the Abstract to provide greater methodological detail and ensure consistency with the Methods section.

2. In lines 42-44, the observed between-group reduction in pain exceeded the reported minimal clinically important difference for acute postoperative pain, indicating a clinically meaningful treatment effect. Please specify the MCID value.

Response: Thank you for this valuable comment. The MCID value (9.9 mm) has now been specified in the Abstract.

3. Add the statistical value for the TUG and sleep quality.

Response: Thank you for this valuable comment. The statistical significance for the TUG test and sleep quality outcomes has been added to the Abstract.

4. Include the clinical trial registry number at the end of the abstract.

Response: Thank you for this valuable comment. The clinical trial registry number (ClinicalTrials.gov: NCT06910215) has been added at the end of the Abstract.

Introduction

5. Define ETT and elaborate on its physiological and biomechanical function.

Response: Thank you for this valuable comment. The Introduction has been revised to define ETT more clearly and to briefly describe its proposed physiological and biomechanical mechanisms of action.

6. Explain additional therapeutic management for coronary artery bypass grafting (CABG) patients following immediate postoperative care and prior to discharge.

Response: Thank you for this valuable comment. We have expanded the Methods section to describe the standard postoperative management provided to all participants from the immediate postoperative period until hospital discharge.

7. In line 74, add the word 'objective' or 'aim' of the study.

Response: Thank you for this valuable comment. We have revised the final paragraph of the Introduction to explicitly state the objective of the study.

Materials and methods

8. Specify the study setting and period in accordance with the study design.

Response: Thank you for this valuable comment. The study setting and study period have been explicitly added to the Study design section of the Methods.

9. Provide details on the randomization sequence and blinding in a separate heading.

Response: Thank you for this valuable comment. We have created a separate subsection entitled “Randomization and blinding” and moved the relevant methodological details to this section for improved clarity.

10. Include a link to the clinical trial registration details after the registry number.

Response: Thank you for this valuable comment. A direct link to the ClinicalTrials.gov registration record has been added after the trial registration number.

11. In line 115, expand on the baseline assessment.

Response: Thank you for this valuable comment. The description of the baseline assessment has been expanded to clarify the timing and the outcome measures assessed before the intervention.

12. Clarify the inclusion criteria.

Response: Thank you for this valuable comment. The inclusion criteria have been clarified to provide a more complete description of participant eligibility and to ensure consistency with the registered study protocol.

13. Explain the duration of taping applications for all three groups.

Response: Thank you for this valuable comment. The duration of the taping application has been clarified for all three groups in the Methods section.

14. Add images of all taping procedures in accordance with ethical guidelines.

Response: Thank you for this valuable suggestion. Representative photographs of the elastic therapeutic taping, placebo taping, and sham taping procedures have been added as Figure 2. In addition, the Ethics section has been revised to clarify that written informed consent for publication of the clinical photographs was obtained from all participants whose images are included in the manuscript.

15. In line 185, elaborate on the length of stay (LOS).

Response: Thank you for this valuable comment. The description of length of hospital stay (LOS) has been expanded to clarify its definition and the routine clinical discharge process.

16. Include validity and reliability information for all outcome parameters and interventions, citing recent references.

Response: Thank you for this valuable comment. We have expanded the Methods section to provide validity and reliability information for the patient-reported and functional outcome measures. We also clarified the measurement procedures for SpO₂, analgesic consumption, LOS, and the standardization of the taping interventions, with additional references where appropriate.

Results and Discussion

17. Separate the results and discussion into distinct headings.

Response: Thank you for this valuable suggestion. We appreciate this recommendation. However, the manuscript has been prepared in accordance with the PLOS ONE manuscript formatting guidelines, which present the Results and discussion section as a combined heading. Therefore, we have retained the current structure to ensure consistency with the journal's required format.

18. Table 1 separates the numbers of male and female participants but omits surgical data when information is missing.

Response: Thank you for this comment. The variables presented in Table 1 correspond to the predefined baseline characteristics collected for this randomized controlled trial. Detailed intraoperative surgical variables were not predefined study variables and therefore were not collected.

19. Please add the p-value in Table 1.

Response: Thank you for this valuable suggestion. P values have now been added to Table 1 for all baseline comparisons.

20. In line 362, the statement that effective early pain control may limit central sensitization and reduce the risk of chronic pain is supported. Please add a reference for the above sentence.

Response: Thank you for this valuable suggestion. We have added appropriate references to support the statement regarding the potential role of effective early pain control in limiting central sensitization and reducing the risk of chronic postoperative pain.

21. In lines 369-371, the marked reduction in total analgesic and tramadol consumption in the ETT group confirms that the intervention not only alters subjective pain perception but also reduces objective medication requirements [6]. How do the authors ascertain that pain reduction is solely due to the elastic taping method?

Response: Thank you for this important comment. We agree that the original wording was too definitive. Accordingly, we revised the Discussion by replacing "confirms" with "suggests" and modified the text to avoid implying that the observed reduction in analgesic consumption was solely attributable to ETT.

22. In lines 417-418, the claim that ETT may indirectly improve sleep by reducing pain and stabilizing the chest wall needs a supporting reference.

Response: Thank you for this valuable suggestion. We have revised the statement and added two appropriate references to support the relationship between postoperative pain reduction and improved sleep quality.

Reviewer #5: This revision addresses the vast majority of concerns raised during peer review. The manuscript is methodologically stronger, better written, and considerably more transparent than the original submission. The remaining issues are relatively minor and relate primarily to reporting clarity rather than fundamental flaws in the study design or results.

1. Modified intention-to-treat terminology still needs refinement: The authors now state that a modified intention-to-treat (mITT) analysis was performed, which is more appropriate than the previous ITT claim. However, the manuscript states:

"including all randomized participants who received the allocated intervention and had evaluable post-intervention outcome data"

This is not a standard mITT definition, since patients without outcome data were excluded entirely.

The authors should:

• explicitly define mITT according to CONSORT recommendations;

• explain why no imputation was performed;

• clarify that the analysis is essentially a complete-case modified ITT.

Response: Thank you for this important comment. We have revised the Statistical Analysis section to clarify the definition of the modified intention-to-treat approach, stated that no imputation of missing outcome data was performed, and specified that the primary analysis represents a complete-case modified intention-to-treat analysis.

2. Randomization and allocation concealment remain incompletely described

The revision improves the description of randomization, but allocation concealment is still insufficiently reported. The manuscript states:

Group assignments were implemented according to the pre-generated sequence.

This does not explain:

• Were opaque-sealed envelopes used?

• Was allocation concealed electronically?

• Who had access to the randomization list?

• Could recruiters predict upcoming allocations?

Without this information, selection bias cannot be fully assessed.

Response: Thank you for this important comment. We have revised the Randomization and blinding section to provide additional details on the allocation procedure. We clarified that the computer-generated randomization sequence was generated before participant recruitment by the corresponding author. The sequence was not accessible to the cardiovascular surgery team responsible for participant enrollment. Group allocation was assigned only after participant enrollment according to the pre-generated randomization sequence. The physiotherapist responsible for tape application was informed of the assigned group immediately before tape application

3. Blinding remains potentially vulnerable

The authors acknowledge that blinding success was not assessed.

This is appropriate. However, the manuscript should discuss that:

• active taping was applied around the sternotomy,

• sham taping was applied on the back,

meaning participants may have recognized group allocation.

This limitation deserves slightly stronger emphasis.

Response: Thank you for this valuable comment. We have revised the Limitations section accordingly. We now acknowledge that blinding success was not formally assessed. We also note that the different taping locations may have allowed some participants to infer their group allocation.

4. Statistical analysis requires additional justification: The authors justify mixed ANOVA by citing robustness. However, VAS values throughout the Results are reported as median (IQR) rather than means. This creates an inconsistency.

If ANOVA was used, authors should explain:

• whether residuals were normally distributed;

• whether assumptions were checked on model residuals rather than raw data;

• why means are not presented for the primary endpoint.

Alternatively, mixed-effects regression would be statistically stronger.

Response: Thank you for this valuable comment. We agree that assessment of model residuals could provide additional information regarding model assumptions. To improve consistency between the statistical analysis and data presentation, the primary outcome is now presented as mean ± standard deviation, consistent with the use of the two-way mixed ANOVA. We also clarified the rationale for using the mixed ANOVA in the Statistical Analysis section.

5. Hospital stay remains vulnerable to confounding

The Discussion has become more cautious, which is appreciated.

However, the Results still state:

participants in the ETT group were discharged approximately one day earlier

This wording still implies causality.

Suggested wording:

"Participants receiving ETT had a median hospital stay one day shorter than control groups; however, discharge timing may have been influenced by factors beyond the intervention."

Response: Thank you for this valuable comment. We have revised the wording in the Results section to avoid implying a causal relationship.

6. Table 2 inconsistency: Length of hospital stay was not included in the multiple-comparison adjustment. Yet the response letter states this sentence was removed.

Please ensure the table matches the revised statistical approach.

Response: Thank you for identifying this inconsistency. The statement has been removed from Table 2.

7. Sample size justification: The sample size is based on Cohen's d = 0.49 from King et al. It would strengthen reproducibility if the manuscript briefly explained:

• which outcome from King et al. generated this estimate;

• whether it represents between-group or within-group effect size.

Response: Thank you for this helpful comment. We have clarified that the expected effect size (0.49) was based on the between-group standardized mean difference for postoperative pain reported by King et al.

8. Confidence intervals

Confidence intervals have now been added for several outcomes, which is excellent. Consider also reporting:

• confidence intervals for LOS

• confidence intervals for sleep quality

to maintain consistency across secondary outcomes.

Response: Thank you for this valuable suggestion. Confidence intervals were reported for the primary outcomes. As sleep quality and length of hospital stay were analyzed using non-parametric methods and are presented as median (IQR), confidence intervals were not added to maintain consistency with the statistical approach used

9. Correlation analysis

The correlation analysis appears exploratory. The manuscript should clearly state:

"These analyses were exploratory and hypothesis-generating." Otherwise readers may overinterpret them.

Response: Thank you for this valuable comment. We have clarified in both the Results and Discussion sections that the correlation analyses were exploratory and hypothesis-generating

10. Language: English has improved considerably.

Only a few small issues remain.

Examples: "with scored recorded" should be "with scores recorded"

"Participant recruitment continued until the predetermined sample size of 195 participants who completed the study was achieved." This sentence could be smoother.

Suggested: "Recruitment continued until the target number of 195 evaluable participants was achieved."

Response: Thank you for your helpful suggestions. The identified language issues have been corrected, and the manuscript has been carefully proofread.

11. CONSORT flow Ensure Figure 1 clearly distinguishes:

• randomized

• received intervention

• lost to follow-up

• analyzed (modified ITT)

using standard CONSORT terminology.

Response: Thank you for this helpful suggestion. Figure 1 has been revised using standard CONSORT terminology.

12. Abstract: The abstract is much improved.

However, "Functional mobility and sleep quality improved." should specify "compared with placebo and sham groups" to avoid ambiguity.

Response: Thank you for this helpful suggestion. The Abstract has been revised to clarify that improvements in functional mobility and sleep quality were observed compared with the placebo and sham groups.

Reviewer #6: Based on the reviewed revision manuscript, the article is fully suitable for publication in terms of scientific depth, statistical accuracy, methodological strength, transparency, and compliance with the open data policy. The authors have responded to all major and minor criticisms (mITT conversion, statistical justifications, removal of speculative statements, quality of visuals) from the editor and the three reviewers in a highly professional and satisfactory manner. The only potential risk facing the article, the fact that the study was recorded retrospectively, is explicitly acknowledged as a limitation in the revision text, and since the data is transparently disclosed in Zenodo, it will not pose an obstacle. It is anticipated that the journal editorial board will approve this revision and the article will be accepted and published. This is a highly successful revision manuscript with a very high chance of publication.

Response: We sincerely thank the reviewer for the positive evaluation of our manuscript and for recognizing the improvements made during the revision process. We greatly appreciate your encouraging comments.

Attachment Submitted filename: Response_to_Reviewers_auresp_2.docx

# Peer review: Decision Letter 2

**DOI:** 10.1371/journal.pone.0359282.r005

**Authors:** Burak Katipoğlu

12 Aug 2026

Dear Dr. Özel,

Thank you for submitting your manuscript to PLOS ONE. After careful consideration, we feel that it has merit but does not fully meet PLOS ONE’s publication criteria as it currently stands. Therefore, we invite you to submit a revised version of the manuscript that addresses the points raised during the review process.

Please submit your revised manuscript by Sep 26 2026 11:59PM. If you will need more time than this to complete your revisions, please reply to this message or contact the journal office at plosone@plos.org. When you're ready to submit your revision, log on to https://www.editorialmanager.com/pone/ and select the 'Submissions Needing Revision' folder to locate your manuscript file.

- A letter that responds to each point raised by the academic editor and reviewer(s). You should upload this letter as a separate file labeled 'Response to Reviewers'.
- A marked-up copy of your manuscript that highlights changes made to the original version. You should upload this as a separate file labeled 'Revised Manuscript with Track Changes'.
- An unmarked version of your revised paper without tracked changes. You should upload this as a separate file labeled 'Manuscript'.

If you would like to make changes to your financial disclosure, please include your updated statement in your cover letter. Guidelines for resubmitting your figure files are available below the reviewer comments at the end of this letter.

If applicable, we recommend that you deposit your laboratory protocols in protocols.io to enhance the reproducibility of your results. Protocols.io assigns your protocol its own identifier (DOI) so that it can be cited independently in the future. For instructions see: https://journals.plos.org/plosone/s/submission-guidelines#loc-laboratory-protocols. Additionally, PLOS ONE offers an option for publishing peer-reviewed Lab Protocol articles, which describe protocols hosted on protocols.io. Read more information on sharing protocols at https://plos.org/protocols?utm_medium=editorial-email&utm_source=authorletters&utm_campaign=protocols.

As the corresponding author, your ORCID iD is verified in the submission system and will appear in the published article. PLOS supports the use of ORCID, and we encourage all coauthors to register for an ORCID iD and use it as well. Please encourage your coauthors to verify their ORCID iD within the submission system before final acceptance, as unverified ORCID iDs will not appear in the published article. *Only*  the individual author can complete the verification step; PLOS staff *cannot*  verify ORCID iDs on behalf of authors.

We look forward to receiving your revised manuscript.

Kind regards,

Burak Katipoğlu

Academic Editor

PLOS One

Journal Requirements:

If the reviewer comments include a recommendation to cite specific previously published works, please review and evaluate these publications to determine whether they are relevant and should be cited. There is no requirement to cite these works unless the editor has indicated otherwise.

Please review your reference list to ensure that it is complete and correct. If you have cited papers that have been retracted, please include the rationale for doing so in the manuscript text, or remove these references and replace them with relevant current references. Any changes to the reference list should be mentioned in the rebuttal letter that accompanies your revised manuscript. If you need to cite a retracted article, indicate the article’s retracted status in the References list and also include a citation and full reference for the retraction notice.

Additional Editor Comments (if provided):

Dear Authors,

On the methodological side, the single point to complete in the main text is the allocation concealment method. The text does not state whether sequentially numbered opaque sealed envelopes, centralized allocation, or another method was used. This information is needed so that the reader can assess the risk of selection bias, and it should be added in concrete terms.

The abstract still reports the primary outcome as median with interquartile range, whereas the main text and tables use mean with standard deviation. The resting and coughing pain values in the abstract should be brought into line with that format. A passage appears almost word for word twice in the discussion, and this repetition should be removed. Internally conflicting statements should also be corrected, for example the mismatch between the publication-consent statement and the presence of patient photographs. Finally, a language edit across the manuscript to remove a few conversational expressions would be appropriate.

Our decision is minor revision.

Sincerely,

[Note: HTML markup is below. Please do not edit.]

Reviewers' comments:

Reviewer's Responses to Questions

**Comments to the Author**

Reviewer #1: All comments have been addressed

Reviewer #4: All comments have been addressed

Reviewer #5: All comments have been addressed

Reviewer #6: All comments have been addressed

**********

2. Is the manuscript technically sound, and do the data support the conclusions?

Reviewer #1: Yes

Reviewer #4: Yes

Reviewer #5: Yes

Reviewer #6: Yes

**********

3. Has the statistical analysis been performed appropriately and rigorously?

Reviewer #1: Yes

Reviewer #4: Yes

Reviewer #5: Yes

Reviewer #6: Yes

**********

4. Have the authors made all data underlying the findings in their manuscript fully available?

The PLOS Data policy

Reviewer #1: Yes

Reviewer #4: Yes

Reviewer #5: Yes

Reviewer #6: Yes

**********

5. Is the manuscript presented in an intelligible fashion and written in standard English?

Reviewer #1: Yes

Reviewer #4: Yes

Reviewer #5: Yes

Reviewer #6: Yes

**********

Reviewer #1: he study design is robust, the methodology is sound, and the results are presented clearly. I am pleased to recommend acceptance of this manuscript for publication in its current form.

Reviewer #4: Thank you for addressing all the comments. I hope this article proves beneficial for practicing therapists using the elastic taping method and maximizes the benefits for post-operative CABG patients. Best wishes.

Reviewer #5: 1. Statistical reporting is much more consistent: The manuscript now clearly states that:

• the primary endpoint was analyzed using a prespecified two-way mixed ANOVA

• secondary outcomes were analyzed using appropriate nonparametric methods

• Bonferroni correction was applied

• effect sizes are reported

• confidence intervals are reported for primary outcomes

• mITT is explicitly described as a complete-case modified intention-to-treat analysis

2. Randomization and blinding: The Randomization section is now much clearer. It specifies

• computer-generated block randomization

• sequence generated before recruitment

• recruitment team had no access

• treating physiotherapist separated from outcome assessment

• assessor blinded

These additions substantially reduce concerns about selection bias.

3. Discussion is much more balanced: Previously several statements implied causality. Now wording such as

"suggests", "may", "potential explanation" is used appropriately. This is exactly what PLOS editors expect.

4. CONSORT compliance: The manuscript now includes

• trial registration

• flow diagram revisions

• clearer inclusion criteria

• intervention description

• photographs

• safety monitoring

• adverse events

• data availability

This considerably improves transparency.

5.Duplicate paragraph: There is one obvious duplication. The section: Clinical implications and safety appears twice almost word-for-word. Pages 23–24 contain repeated text. This duplication should be removed before acceptance.

6.One inconsistency still exists: In the Abstract pain values are still reported as median (IQR) for the primary outcome although the manuscript now states that the primary analysis uses mixed ANOVA and Table 2 reports mean ± SD. For consistency, the Abstract should also report: mean ± SD instead of median (IQR). This is probably the only remaining statistical inconsistency.

7.Allocation concealment: The response letter explains allocation concealment well. However, the manuscript still never explicitly states whether allocation was performed using sequential opaque envelopes or centralized allocation or another concealment method. Instead it says group allocation was assigned according to the pre-generated sequence This is improved but still not ideal according to CONSORT. If the actual method was opaque envelopes, it should be explicitly stated. Otherwise reviewers may ask again.

8.One sentence remains awkward: Page 20: "The significant decrease in pain that is caused by movement..."

A smoother wording would be "The significant reduction in movement-evoked pain suggests..." This is purely stylistic.

9.English polishing: The English is greatly improved. I still noticed occasional phrases such as wasn't optimally set up which sound conversational for a scientific manuscript. For example:

"mechanical placement that wasn't optimally set up..." could become "suboptimal tape placement" which is more scientific.

10.Correlation section: You correctly state exploratory and hypothesis-generating which addresses the reviewer. Excellent improvement.

11.Discussion: The Discussion is now one of the strongest parts. It

• compares previous studies

• explains mechanisms cautiously

• discusses opioid reduction

• explains LOS carefully

• acknowledges limitations

Reviewer #6: All necessary arrangements have been made. I believe the article is ready for publication. Best wishes

**********

what does this mean?). If published, this will include your full peer review and any attached files.

If you choose “no”, your identity will remain anonymous but your review may still be made public.

**Do you want your identity to be public for this peer review?**  For information about this choice, including consent withdrawal, please see our Privacy Policy

Reviewer #1: No

Reviewer #4: **Yes:**  SHAHUL HAMEED PAKKIR MOHAMED

Reviewer #5: No

Reviewer #6: **Yes:**  Esedullah AKARAS

**********

[NOTE: If reviewer comments were submitted as an attachment file, they will be attached to this email and accessible via the submission site. Please log into your account, locate the manuscript record, and check for the action link "View Attachments". If this link does not appear, there are no attachment files.]

To ensure your figures meet our technical requirements, please review our figure guidelines: https://journals.plos.org/plosone/s/figures

You may also use PLOS’s free figure tool, NAAS, to help you prepare publication quality figures: https://journals.plos.org/plosone/s/figures#loc-tools-for-figure-preparation.

NAAS will assess whether your figures meet our technical requirements by comparing each figure against our figure specifications.

# Author comment: Author response to Decision Letter 3

**DOI:** 10.1371/journal.pone.0359282.r006

14 Aug 2026

We sincerely thank the Academic Editor and the reviewers for their careful evaluation of our revised manuscript and for their constructive comments. We greatly appreciate their recognition of the improvements made during the previous revision rounds.

Response to the Academic Editor

On the methodological side, the single point to complete in the main text is the allocation concealment method. The text does not state whether sequentially numbered opaque sealed envelopes, centralized allocation, or another method was used. This information is needed so that the reader can assess the risk of selection bias, and it should be added in concrete terms.

The abstract still reports the primary outcome as median with interquartile range, whereas the main text and tables use mean with standard deviation. The resting and coughing pain values in the abstract should be brought into line with that format. A passage appears almost word for word twice in the discussion, and this repetition should be removed. Internally conflicting statements should also be corrected, for example the mismatch between the publication-consent statement and the presence of patient photographs. Finally, a language edit across the manuscript to remove a few conversational expressions would be appropriate

Response: We thank the Academic Editor for the careful assessment and constructive guidance. We have now described the allocation concealment method in concrete terms in the Randomization and blinding section. The pain values in the Abstract have been changed from median (IQR) to mean ± SD to ensure consistency with Table 2. We removed the duplicated passage from the Discussion and corrected the Consent for publication statement regarding the clinical photographs. We also revised the manuscript to replace conversational expressions with more formal scientific language.

Review Comments to the Author

Reviewer #5:

1. Statistical reporting is much more consistent: The manuscript now clearly states that:

• the primary endpoint was analyzed using a prespecified two-way mixed ANOVA

• secondary outcomes were analyzed using appropriate nonparametric methods

• Bonferroni correction was applied

• effect sizes are reported

• confidence intervals are reported for primary outcomes

• mITT is explicitly described as a complete-case modified intention-to-treat analysis

Response: Thank you for your positive assessment.

2. Randomization and blinding: The Randomization section is now much clearer. It specifies

• computer-generated block randomization

• sequence generated before recruitment

• recruitment team had no access

• treating physiotherapist separated from outcome assessment

• assessor blinded

These additions substantially reduce concerns about selection bias.

Response: Thank you for your positive assessment.

3. Discussion is much more balanced: Previously several statements implied causality. Now wording such as "suggests", "may", "potential explanation" is used appropriately. This is exactly what PLOS editors expect.

Response: Thank you for your positive assessment.

4. CONSORT compliance: The manuscript now includes

• trial registration

• flow diagram revisions

• clearer inclusion criteria

• intervention description

• photographs

• safety monitoring

• adverse events

• data availability

This considerably improves transparency.

Response: Thank you for your positive assessment.

5.Duplicate paragraph: There is one obvious duplication. The section: Clinical implications and safety appears twice almost word-for-word. Pages 23–24 contain repeated text. This duplication should be removed before acceptance.

Response: Thank you for noting this duplication. The repeated text has been removed.

6.One inconsistency still exists: In the Abstract pain values are still reported as median (IQR) for the primary outcome although the manuscript now states that the primary analysis uses mixed ANOVA and Table 2 reports mean ± SD. For consistency, the Abstract should also report: mean ± SD instead of median (IQR). This is probably the only remaining statistical inconsistency.

Response: Thank you for noting this inconsistency. The pain values in the Abstract have been revised to mean ± SD in accordance with Table 2.

7.Allocation concealment: The response letter explains allocation concealment well. However, the manuscript still never explicitly states whether allocation was performed using sequential opaque envelopes or centralized allocation or another concealment method. Instead it says group allocation was assigned according to the pre-generated sequence This is improved but still not ideal according to CONSORT. If the actual method was opaque envelopes, it should be explicitly stated. Otherwise reviewers may ask again.

Response: Thank you for highlighting this point. The corresponding author retained the sequence and disclosed the assignment to the treating physiotherapist only after participant enrollment had been completed. We have clarified the allocation concealment method in the Randomization and blinding section.

8.One sentence remains awkward: Page 20: "The significant decrease in pain that is caused by movement..."

A smoother wording would be "The significant reduction in movement-evoked pain suggests..." This is purely stylistic.

Response: Thank you for this suggestion. The sentence has been revised as recommended.

9.English polishing: The English is greatly improved. I still noticed occasional phrases such as wasn't optimally set up which sound conversational for a scientific manuscript. For example: "mechanical placement that wasn't optimally set up..." could become "suboptimal tape placement" which is more scientific.

Response: Thank you for this suggestion. The phrase has been revised using more formal scientific language.

10.Correlation section: You correctly state exploratory and hypothesis-generating which addresses the reviewer. Excellent improvement.

Response: Thank you for your positive assessment.

11.Discussion: The Discussion is now one of the strongest parts. It

• compares previous studies

• explains mechanisms cautiously

• discusses opioid reduction

• explains LOS carefully

• acknowledges limitations

Response: Thank you for your positive assessment.

Attachment Submitted filename: Response_to_Reviewers_auresp_3.docx

# Peer review: Decision Letter 3

**DOI:** 10.1371/journal.pone.0359282.r007

**Authors:** Burak Katipoğlu

13 Sep 2026

Effectiveness of Elastic Therapeutic Taping in Pain Management After Median Sternotomy: A Randomized Controlled Trial

PONE-D-26-16084R3

Dear Dr. Özel,

We’re pleased to inform you that your manuscript has been judged scientifically suitable for publication and will be formally accepted for publication once it meets all outstanding technical requirements.

Within one week, you’ll receive an e-mail detailing the required amendments. When these have been addressed, you’ll receive a formal acceptance letter and your manuscript will be scheduled for publication.

An invoice will be generated when your article is formally accepted. Please note, if your institution has a publishing partnership with PLOS and your article meets the relevant criteria, all or part of your publication costs will be covered. Please make sure your user information is up-to-date by logging into Editorial Manager at Editorial Manager® and clicking the ‘Update My Information' link at the top of the page. For questions related to billing, please contact billing support.

If your institution or institutions have a press office, please notify them about your upcoming paper to help maximize its impact. If they’ll be preparing press materials, please inform our press team as soon as possible -- no later than 48 hours after receiving the formal acceptance. Your manuscript will remain under strict press embargo until 2 pm Eastern Time on the date of publication. For more information, please contact onepress@plos.org.

Kind regards,

Burak Katipoğlu

Academic Editor

PLOS One

Additional Editor Comments (optional):

Dear Authors,

Your constructive engagement throughout the revision process moved the manuscript forward considerably.

The manuscript is accepted. I believe your study will contribute to the field, and I congratulate you on this careful work.

Sincerely,

Reviewers' comments:

Reviewer's Responses to Questions

**Comments to the Author**

Reviewer #2: All comments have been addressed

**********

2. Is the manuscript technically sound, and do the data support the conclusions?

Reviewer #2: Yes

**********

3. Has the statistical analysis been performed appropriately and rigorously?

Reviewer #2: Yes

**********

4. Have the authors made all data underlying the findings in their manuscript fully available?

The PLOS Data policy

Reviewer #2: Yes

**********

5. Is the manuscript presented in an intelligible fashion and written in standard English?

Reviewer #2: Yes

**********

Reviewer #2: I appreciate the opportunity to review manuscript #PONE-D-26-16084R3 titled “Effectiveness of Elastic Therapeutic Taping in Pain Management After Median Sternotomy: A Randomized Controlled Trial". I have no further comments or suggestions for improvement.

**********

what does this mean?). If published, this will include your full peer review and any attached files.

If you choose “no”, your identity will remain anonymous but your review may still be made public.

**Do you want your identity to be public for this peer review?**  For information about this choice, including consent withdrawal, please see our Privacy Policy

Reviewer #2: No

**********

# Editor report: Acceptance letter

**DOI:** 10.1371/journal.pone.0359282.r008

**Authors:** Burak Katipoğlu

PONE-D-26-16084R3

PLOS One

Dear Dr. Özel,

I'm pleased to inform you that your manuscript has been deemed suitable for publication in PLOS One. Congratulations! Your manuscript is now being handed over to our production team.

At this stage, our production department will prepare your paper for publication. This includes ensuring the following:

* All references, tables, and figures are properly cited

* All relevant supporting information is included in the manuscript submission,

* There are no issues that prevent the paper from being properly typeset

You will receive further instructions from the production team, including instructions on how to review your proof when it is ready. Please keep in mind that we are working through a large volume of accepted articles, so please give us a few days to review your paper and let you know the next and final steps.

Lastly, if your institution or institutions have a press office, please let them know about your upcoming paper now to help maximize its impact. If they'll be preparing press materials, please inform our press team within the next 48 hours. Your manuscript will remain under strict press embargo until 2 pm Eastern Time on the date of publication. For more information, please contact onepress@plos.org.

You will receive an invoice from PLOS for your publication fee after your manuscript has reached the completed accept phase. If you receive an email requesting payment before acceptance or for any other service, this may be a phishing scheme. Learn how to identify phishing emails and protect your accounts at https://explore.plos.org/phishing.

If we can help with anything else, please email us at customercare@plos.org.

Thank you for submitting your work to PLOS One and supporting open access.

Kind regards,

PLOS One Editorial Office Staff

on behalf of

Dr. Burak Katipoğlu

Academic Editor

PLOS One
